# Multicomponent Synthesis of New Fluorescent Boron Complexes Derived from 3-Hydroxy-1-phenyl-1*H*-pyrazole-4-carbaldehyde

**DOI:** 10.3390/molecules29143432

**Published:** 2024-07-22

**Authors:** Viktorija Savickienė, Aurimas Bieliauskas, Sergey Belyakov, Eglė Arbačiauskienė, Algirdas Šačkus

**Affiliations:** 1 Department of Organic Chemistry, Kaunas University of Technology, Radvilėnų pl. 19, LT-50254 Kaunas, Lithuania; viktorija.dargyte@ktu.lt; 2 Institute of Synthetic Chemistry, Kaunas University of Technology, K. Baršausko g. 59, LT-51423 Kaunas, Lithuania; aurimas.bieliauskas@ktu.lt; 3 Latvian Institute of Organic Synthesis, Aizkraukles 21, LV-1006 Riga, Latvia; serg@osi.lv

**Keywords:** pyrazoles, iminoboronates, fluorescence, aggregation-induced emission enhancement

## Abstract

Novel fluorescent pyrazole-containing boron (III) complexes were synthesized employing a one-pot three-component reaction of 3-hydroxy-1-phenyl-1*H*-pyrazole-4-carbaldehyde, 2-aminobenzenecarboxylic acids, and boronic acids. The structures of the novel heterocyclic compounds were confirmed using ^1^H-, ^13^C-, ^15^N-, ^19^F-, and ^11^B-NMR, IR spectroscopy, HRMS, and single-crystal X-ray diffraction data. The photophysical properties of the obtained iminoboronates were investigated using spectroscopic techniques, such as UV–vis and fluorescence spectroscopies. Compounds display main UV–vis absorption maxima in the blue region, and fluorescence emission maxima are observed in the green region of the visible spectrum. It was revealed that compounds exhibit fluorescence quantum yield up to 4.3% in different solvents and demonstrate an aggregation-induced emission enhancement effect in mixed THF–water solutions.

## 1. Introduction

Heterocyclic systems possessing a pyrazole ring have found diverse applications in various fields, including pharmaceuticals [1,2], agrochemicals [3,4], and functional materials [5,6]. Among other heterocycles, pyrazole-ring-containing compounds often exhibit anticancer [7,8], anti-inflammatory [9], and antidiabetic activities [10]. Moreover, it is a common structural motif in a variety of drug substances. Anti-inflammatory celecoxib [11], antipsychotic 3-cyano-*N*-(1,3-diphenyl-1*H*-pyrazol-5-yl)benzamide (CDPPB) [12], antiobesity rimonabant [13], and analgesic difenamizole [14] can be highlighted as notable examples. Investigations toward agrochemical compounds have also led to the discovery of pyrazole-containing fungicides [15], insecticides [16], and herbicides [17]. In material sciences, pyrazole compounds have emerged as chemosensors [18], biological imaging agents [19], and metal–organic frameworks (MOFs) [20].

Over the years, many methods have been used to synthesize pyrazole derivatives, including ring formation via the cyclocondensation of hydrazines, with carbonyl systems and dipolar cycloadditions being the most relevant [21,22]. Another approach to synthesizing pyrazole derivatives is related to the functionalization of pyrazole ring, and a number of reported synthetic late-stage pyrazole functionalizations rely on (pseudo)halogenation and following transition-metal catalysis or direct C-H functionalization [23,24]. A multicomponent reaction (MCR) approach has also been successfully applied to synthesize pyrazole-containing heterocyclic systems [25,26]. Multicomponent reactions can be highlighted as one of the most valuable tools used by researchers to synthesize heterocyclic compounds, as they meet the principles of sustainable chemistry, are cost- and time-effective, and are atom- and bond-economic [27,28]. Recent examples of multicomponent reactions for synthesizing pyrazole-containing compounds include the synthesis of biheterocyclic pyrazole-linked thiazole or imidazole derivatives from appropriate aryl glyoxal, aryl thioamide, and pyrazolones [29] or the formation of stable pyrazole amides via a Ugi four-component reaction [30]. A multicomponent synthesis approach was applied to obtain multisubstituted pyrazole derivatives from alkynes, nitriles, and titanium imido complexes [31]. We have also reported the synthesis and structural elucidation of several series of novel biheterocyclic pyrazole-containing compounds by employing different types of multicomponent reactions, starting from 3-alkoxy-1*H*-pyrazole-4-carbaldehydes [32].

The boron (III)-containing complexes, especially the tetracoordinated species, have emerged as organic fluorophores. The boron-dipyrromethene (BODIPY) family of dyes have proved their superior characteristics as they exhibit excellent optical properties, are compatible with a wide range of reaction conditions, easy to functionalize, stable as solids and solutions [33,34,35,36]. The wide application of BODIPY dyes includes cell imaging [37,38], photodynamic therapy [39,40,41], metal ion detection [42,43]. As one of the alternatives for the BODIPYs, also the condensed complexes of boronic acids and salicylidenehydrazones (BASHYs) have been reported [44,45,46,47,48,49,50,51]. Compounds display low luminescence in organic solvents but demonstrate a remarkable aggregation-induced emission enhancement (AIEE) effect. BASHYs have been widely investigated and proven to be interesting as highly fluorescent dyes [44,46,51] or multivalent cancer-cell-targeting drug conjugates [47,48]. However, the synthesis and investigation of boron complexes using heterocyclic carbaldehydes with carbaldehyde moiety adjacent to the hydroxy group instead of salicylic counterparts is still limited.

In continuation of our interest in synthesizing and investigating the properties of various pyrazole derivatives [52,53,54,55,56,57], herein, we report an efficient one-pot multicomponent synthesis of novel pyrazole ring-containing iminoboronates, starting from 3-hydroxy-1-phenyl-1*H*-pyrazole-4-carbaldehyde, and investigate the photophysical properties of the obtained boronic compounds.

## 2. Results and Discussion

### 2.1. Chemistry

The synthesis of iminoboronates is usually achieved via a one-pot, three-component reaction of salicylaldehydes and anthranilic acids condensing to corresponding imines and double condensation with boronic acids. The reaction requires no catalyst, and usually, target products are formed while refluxing the reaction mixture in methanol [45], ethanol [46], acetonitrile [44,47,48], carbon tetrachloride [49], or water [50] media.

**Scheme 1:**
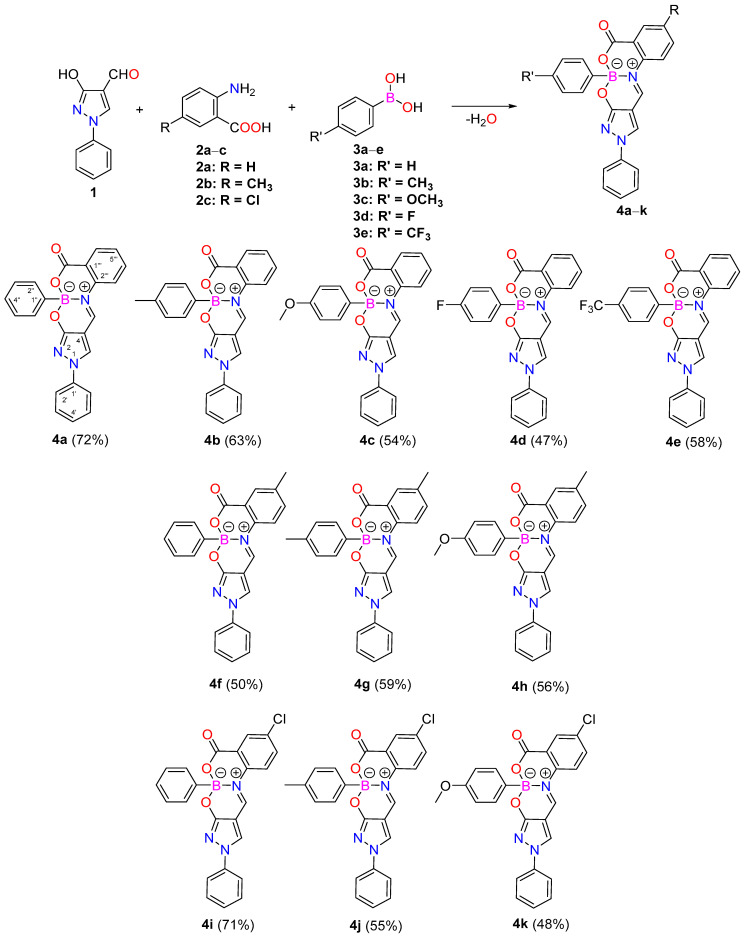
Synthesis of compounds **4a**–**k**. Reagents and conditions: EtOH, 70 °C, 48 h.

The synthesis of the pyrazole-containing organoboron compounds **4a**–**k** was accomplished by starting from 3-hydroxy-1-phenyl-1*H*-pyrazole-4-carbaldehyde (**1**) [58], 2-aminobenzenecarboxylic acids (**2a**–**c**), and boronic acids (**3a**–**d**), and employing an MCR approach (Scheme 1). Refluxing the reaction mixture in ethanol for 48 h, cooling, filtrating, and washing the formed solid provided the target products **4a**–**k**. Efforts to obtain a higher yields by stirring the reaction mixture under MW irradiation [45] or switching the reaction solvent to methanol [47], acetonitrile [44,47,48], or carbon tetrachloride [49] did not improve the results. The scope of the reaction was evaluated using 2-aminobenzenecarboxylic acids substituted with 5-chloro or 5-methyl moieties as well as a variety of boronic acids containing both electron-donating (methyl, methoxy) and electron-withdrawing (fluoro, trifluoromethyl) substituents. The substituents of the reacting materials did not influence the result of the reaction outcome, and the products were formed in a 47–72% yield. All the compounds were obtained as yellow solids stable at ambient conditions. Attempts to obtain an analogous boron complex by employing 4-(diethylamino)phenylboronic acid were unsuccessful, as only unreacted starting materials could be observed in the reaction mixture. The complexes bore a chiral boron center and were obtained as a racemic mixture [45].

### 2.2. NMR Spectroscopic Investigations

**Figure 1:**
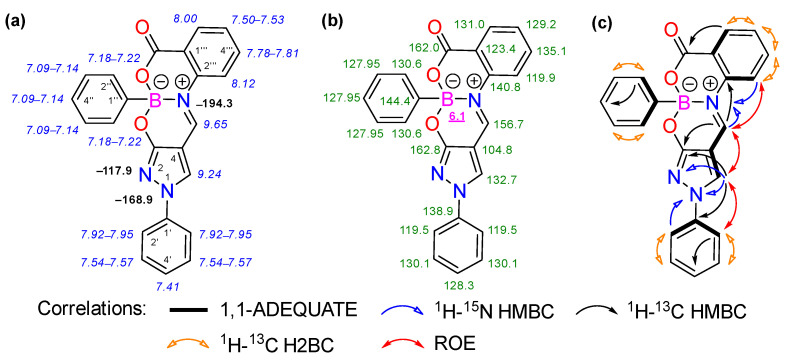
(**a**) ^1^H NMR (italics) and ^15^N NMR (bold) chemical shifts [ppm, ref. TMS (^1^H) and CH_3_NO_2_ (^15^N)] of compound **4a** in DMSO-*d*_6_; (**b**) ^13^C NMR (regular) and ^11^B NMR (underlined) chemical shifts [ppm; ref. TMS] of compound **4a** in DMSO-*d*_6_; (**c**) relevant 1,1-ADEQUATE, ^1^H-^13^C HMBC, ^1^H-^15^N HMBC, ^1^H-^13^C H2BC, and ROE correlations.

The structure of compounds **4a**–**k** was unambiguously assigned based on the HRMS, IR, and NMR spectral data analysis. We have carried out detailed NMR studies for the obtained novel compounds to fully map ^1^H, ^13^C, ^15^N, ^19^F, and ^11^B signals. A detailed analysis of the representative compound **4a** is given in Figure 1a–c. The formation of the N → B coordination bond was evidenced via NMR spectroscopy. The ^11^B NMR spectrum of **4a** displays one broad resonance signal at δ 6.1 ppm, which agrees with the data of other tetracoordinated boron-atom-bearing Schiff bases [44,47]. In addition, the remaining key information for structure elucidation was obtained using two-dimensional NMR spectroscopy techniques such as ^1^H-^1^H ROESY, ^1^H-^13^C HMBC, ^1^H-^13^C H2BC, ^1^H-^15^N HMBC, and 1,1-ADEQUATE (Figure 1c). For instance, the most downfield methine proton in the ^1^H NMR spectrum (singlet, δ 9.65 ppm) was assigned to the new (–CH=N^+^–) bond from the iminoboronate moiety, while another distinct methine signal (singlet, δ 9.24 ppm) was assigned to the pyrazole 5-H proton from the 1*H*-pyrazol-4-yl moiety.

An unambiguous assignment of the aforementioned methine protons was easily achieved from the long-range HMBC correlation data. The pyrazole 5-H proton solely showed ^1^H-^15^N HMBC correlations with the neighboring N-1 “pyrrole-like” (δ −168.9 ppm) and N-2 “pyridine-like” (δ −117.9 ppm) nitrogen atoms. While the methine proton from the iminoboronate moiety correlated with the neighboring nitrogen atom (δ −194.3 ppm) only. These findings were further supported by the distinct long-range ^1^H-^15^N HMBC and ^1^H-^13^C HMBC correlations between the methine protons (phenyl 2′(6′)-H and 3‴-H) from the neighboring aryl moieties and the corresponding carbon or nitrogen atoms.

The ^1^H-^1^H ROESY spectrum of **4a** further elucidated the connectivities based on through-space correlations. For example, distinct ROEs were observed between the pyrazole ring proton 5-H, the phenyl group 2′(6′)-H protons (δ 7.92–7.95 ppm), and the most downfield methine proton from the iminoboronate moiety. The aforementioned methine proton also correlated with the 3‴-H proton (δ 8.12 ppm), confirming their proximity in space. Having successfully identified all the main ^1^H spin systems, the remaining protonated and quaternary carbons were easily assigned from the ^1^H-^13^C H2BC and 1,1-ADEQUATE spectral data.

The newly formed iminoboronates **4a**–**k**, which contain a pyrazole ring, consist of three nitrogen atoms. The chemical shifts of the N-1 “pyrrole-like” and N-2 “pyridine-like” nitrogen atoms from the 1*H*-pyrazol-4-yl moiety were in the ranges from δ −168.0 to −169.3 ppm and from δ −117.3 to −117.9 ppm, respectively. The nitrogen atom in compounds **4a**–**d**,**f**–**h**, from the iminoboronate moiety (–CH=N^+^–), resonated from δ −192.9 to −194.3 ppm, while in the case of compounds **4e**,**i**–**k**, which contained 4″-CF_3_ or 5‴-Cl groups, it resonated slightly upfield in the range from δ −196.8 to −197.9 ppm. The ^11^B NMR spectra of **4a**–**k** exhibited a single broad resonance signal in the range of δ 5.6 to 6.5 ppm. Data analysis showed that the chemical shift values were highly consistent within compounds **4a**–**k**, thus validating the shifts for each position.

### 2.3. Single-Crystal X-ray Diffraction Analysis

**Figure 2:**
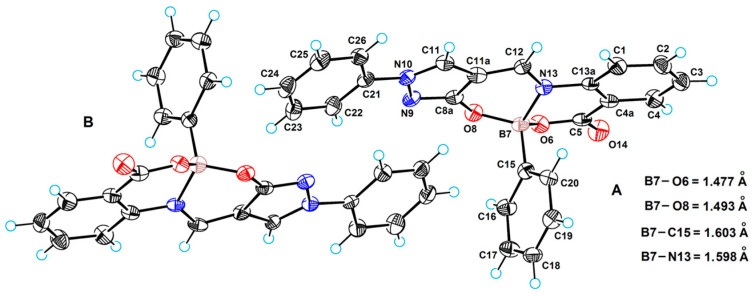
ORTEP diagram of structure **4a** with bond lengths characterizing the tetrahedron of boron (molecules **A** and **B** are connected to each other via a pseudoinversion center).

Suitable crystals of **4a** for X-ray diffraction analysis were obtained from tetrahydrofuran. The asymmetric unit of **4a** contains two independent molecules, **A** and **B** (Figure 2), that are connected to each other via a pseudoinversion center, the coordinates of which, although close to the crystallographic point (¾, ¼, ¼), are still different from it (the real coordinates are 0.7250, 0.2508, and 0.2698). Therefore, it is not possible to increase the symmetry of the crystal structure. Thus, this structure belongs to the triclinic crystal system. The configuration of the asymmetric boron atoms in molecules **A** and **B** are *S*- and *R*-, respectively. Thus, the substance represents a true racemate. 

The heterocyclic system is almost planar; only one boron atom deviates from the plane of the remaining atoms of this system (the deviations are 0.573(7) Å for **A** and 0.588(7) Å for **B**). The plane of the phenyl substituent in position 10 makes a dihedral angle with the plane of the heterocycle equal to 17.3(4)° (in **A**) and 16.1(4)° (in **B**). The phenyl ring in position 7 is perpendicular to the heterocycle; the dihedral angles are 89.7(4)° in **A** and 88.8(4)° in **B**. 

**Figure 3:**
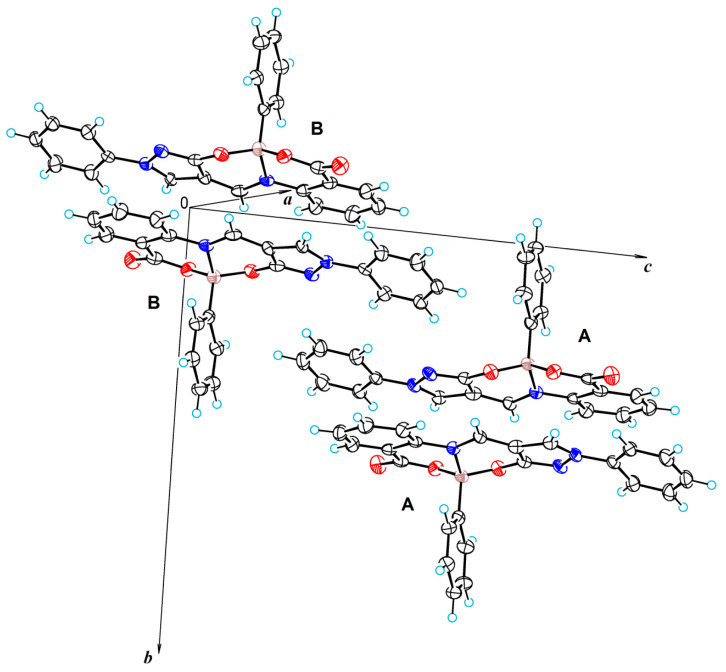
Molecular packing in crystal structure **4a**. Both molecules **A** (as well as molecules **B**) are connected by a crystallographic center of inversion; molecules **B** and **A** are connected by a pseudoinversion center.

There is only a slight π–π interaction between molecules **A** and **B**, with the shortest atom–atom contact being 3.330(6) Å (between N10 and C13). At the same time, molecule **A** forms a strong π–π stacking interaction with the neighboring molecule **A** in the crystal structure. The distance between the least-squares planes of the heterocyclic systems equals 3.159(6) Å. In turn, the **B** molecule in the crystal of the compound also binds to the neighboring **B** molecule through the π–π stacking interaction; in this case, the distance between the least-squares planes of the heterocyclic systems equals 3.278(6) Å. Figure 3 illustrates molecular packing in the unit cell.

### 2.4. Optical Investigations

**Figure 4:**
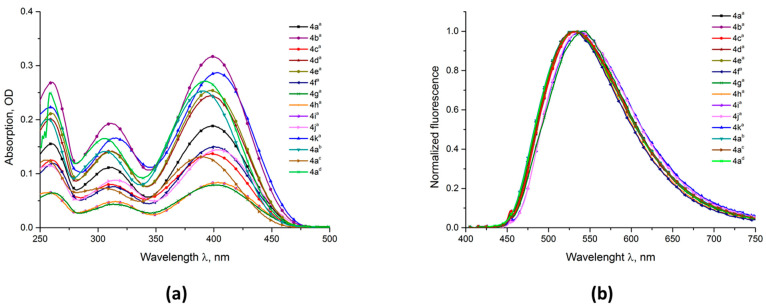
(**a**) UV–vis absorption spectra of compounds **4a**–**k** in different solvents: ^a^ THF, ^b^ MeOH, ^c^ ACN, and ^d^ DMF; (**b**) fluorescence emission spectra (λ_ex_ = 400 nm) of compounds **4a**–**h** in different solvents: ^a^ THF, ^b^ MeOH, ^c^ ACN, and ^d^ DMF.

The UV–vis absorption spectra of compounds **4a**–**k** were first recorded in THF (Figure 4a, Table 1, entries 1–11). The compounds possess the main absorption band in the visible region, at 397–404 nm, and two less intense bands are present in the ultraviolet range, at 309–315 and 258–262 nm. The substituents attached at various positions of the organoboron compounds **4a**–**k** have a negligible effect on the position of the absorption bands.

**Figure 5:**
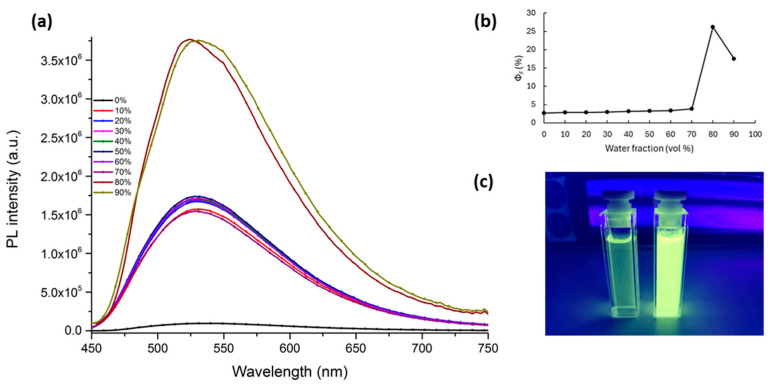
(**a**) Fluorescence emission spectra (λ_ex_ = 440 nm) of compound **4a** in mixed THF–water solutions with different water fractions (0–90%); (**b**) the relationship between the quantum yield of **4a** and the water fraction in mixed THF–water solutions; (**c**) fluorescence photograph of compound **4a** in THF (**left**) and mixed THF–water (20/80) solution (**right**) under 365 nm wavelength UV light.

The fluorescence emissions of compounds **4a**–**k** were first recorded in THF (Figure 4b, Table 1, entries 1–11). The emission maxima are observed at 531–543 nm, and the Stokes shifts are in a range of 128–142 nm. Again, different substituents in the molecular structure of compounds **4a**–**k** do not influence the positions of emission maxima. 4″,5‴-Dimethyl-substituted compound **4g**, and 5‴-chloro-substituted analogues **4i**–**k**, however, show slightly batochromic shifts compared to the other compounds. The dyes display a low quantum yield, ranging from 0.1 to 4.3%, which is in accordance with previously reported observations of similar boron complexes [44]. As iminoboronates are known to possess an AIEE effect [45], we further recorded the emission spectra of compounds **4a**–**k** in mixed THF–water solutions with different water contents (0–90%). As shown in Figure 5a–c, the emission intensity and quantum yield (2.7%) of compound **4a** are lower in the pure THF solution. As the water fraction increases to 80%, the emission intensity increases, and the quantum yield reaches the maximum value of 26.2%, which is about 10-fold that in the pure THF solution. Further increases in the water fraction has a negative effect on the quantum yield. This observation is in agreement with the AIEE phenomenon: when water is added, the compound precipitates, forming regular particles, which leads to enhanced fluorescence intensity and amorphous particles, thus leading to decreased fluorescence intensity [59]. Compounds **4b**–**k** displayed similar behavior (Figures S1–S10).

The optical properties of compound **4a** were further investigated in additional solvents, such as polar protic (MeOH) and polar aprotic (ACN and DMF). As can be observed from UV–vis spectroscopy data, the absorption bands of compound **4a** are slightly blue-shifted in more polar solvents (MeOH, ACN, DMF) in comparison to THF (Table 1, entries 12–14, Figure 4a). Fluorescence emissions of compound **4a** in MeOH, ACN and DMF possess maxima at 531 nm, and the Stokes shifts are 140, 141, and 138 nm, respectively, which are slightly larger comparing to one in THF solution (133 nm) (Table 1, entry 1, Figure 4b). Compound **4a** displays low quantum yields of 3.1, 1.6, and 1.5 in MeOH, ACN, and DMF, respectively (Table 1, entries 12–14).

## 3. Materials and Methods

### 3.1. General

All the starting materials were purchased from commercial suppliers and used as received. Flash column chromatography was performed on silica gel (60 Å; 230–400 µm; supplied by Sigma-Aldrich; Merck KGaA, Darmstadt, Germany). The reaction progress was monitored by TLC analysis on Macherey-Nagel™ ALUGRAM^®^ Xtra SIL G/UV254 plates (Macherey-Nagel GmbH & Co. KG, Düren, Germany) which were visualized by UV light (254 and 365 nm wavelengths). 

Melting points were determined on a Büchi M-565 melting point apparatus and were not corrected. IR spectra of neat samples were recorded on a Bruker Vertex 70v FT-IR spectrometer (Bruker Optik GmbH, Ettlingen, Germany), and the results were reported as the frequency of absorption (cm^−1^). Mass spectra were obtained using a Shimadzu LCMS-2020 (ESI^+^) spectrometer (Shimadzu Corporation, Kyoto, Japan). High-resolution mass spectra were measured on a Bruker MicrOTOF-Q III (ESI^+^) apparatus (Bruker Daltonik GmbH, Bremen, Germany).

^1^H, ^13^C and ^15^N NMR spectra were recorded in DMSO-*d*_6_ solutions at 25 °C on a Bruker Avance III 700 (700 MHz for ^1^H, 176 MHz for ^13^C, and 71 MHz for ^15^N) spectrometer (Bruker BioSpin GmbH, Rheinstetten, Germany) equipped with a 5-mm TCI ^1^H-^13^C/^15^N/D z-gradient cryoprobe. The chemical shifts (δ), were expressed relative to tetramethylsilane (TMS) in ppm. The ^15^N NMR spectra were referenced to neat, external nitromethane (using a coaxial capillary). ^19^F NMR spectra (376 MHz) and ^11^B NMR spectra (128 MHz) were obtained on a Bruker Avance III 400 instrument (Bruker BioSpin AG, Faellanden, Switzerland); with an absolute referencing via *Ξ* ratio was used. The ^1^H, ^13^C and ^15^N NMR resonances were completely and unambiguously assigned using a combination of standard NMR spectroscopic techniques [60], including DEPT, COSY, TOCSY, ROESY, NOESY, gs-HSQC, gs-HMBC, H2BC, LR-HSQMBC and 1,1-ADEQUATE [61].

Single crystals were investigated at 160 K on a Rigaku, XtaLAB Synergy, Dualflex, HyPix diffractometer (Rigaku Corporation, Tokyo, Japan) using monochromated Cu-Kα radiation (λ = 1.54184 Å). The crystal structure of **4a** was solved by direct methods [62] and refined by full-matrix least squares [63]. All nonhydrogen atoms were refined in anisotropical approximation. Hydrogen atoms were refined by riding model with *U*iso(H) = 1.2*U*eq(C). Crystal data for **4a**: triclinic: *a* = 8.0614(2), *b* = 14.5439(5), *c* = 16.3846(7) Å, α = 87.613(3)°, β = 77.887(3)°, γ = 80.845(3)°; *V* = 1854.2(1) Å^3^, *Z* = 4, μ = 0.766 mm^−1^, *D*calc = 1.408 g·cm^−3^; space group is *P*$\bar{1}$. For further details, see crystallographic data for **4a** deposited at the Cambridge Crystallographic Data Centre as Supplementary Publications Number CCDC 2313164. Copies of the data can be obtained, free of charge, on application to CCDC, 12 Union Road, Cambridge CB2 1EZ, UK.

The UV–vis spectra were recorded on a Shimadzu 2600 UV/vis (Shimadzu Corporation, Japan). The fluorescence spectra were recorded on an FL920 fluorescence spectrometer from Edinburgh Instruments (Edinburgh Analytical Instruments Limited, Edinburgh, UK). The PL quantum yields were measured from dilute solutions by an absolute method using the Edinburgh Instruments integrating sphere excited with a Xe lamp. It was ensured that the optical densities of the sample solutions were below 0.1 to avoid reabsorption effects. All the optical measurements were performed at room temperature under ambient conditions.

The following abbreviation is used in reporting the NMR data: Pz, pyrazole. The ^1^H, ^13^C, and ^11^B NMR spectra, as well as the HRMS data of the new compounds, are provided in Figures S11–S56 of the Supplementary Materials.

### 3.2. Synthetic Procedures

General procedure for the compound **4a**–**k** synthesis:

To a mixture of 3-hydroxy-1-phenyl-1*H*-pyrazole-4-carbaldehyde (**1**) (188 mg, 1 mmol) in EtOH (5 mL), appropriate anthranilic acid **2** (1 mmol) and phenylboronic acid **3** (1 mmol) were added. The reaction mixture was stirred at 70 °C for 48 h. After completing the reaction, as determined via TLC, the reaction mixture was cooled to room temperature. Then, the reaction mixture was filtered, and the obtained solid was washed with ethanol and acetone to acquire pure products **4a**–**k**.

**[2-({[3-(hydroxy-κ*O*)-1-phenyl-1*H*-pyrazol-4-yl]methylidene}amino-κ*N*)benzoato(2-)-κ*O*](phenyl)boron (4a)**. Yellow solid; yield 72% (282 mg); m.p. 324–325 °C. IR (v_max_, cm^−1^): 3127 (CH_arom._), 1696, 1627, 1582, 1404 (C=O, C=N, C=C), 1175, 1026 (C–O), 758, 686 (C=C benzene). ^1^H NMR (700 MHz, DMSO-*d*_6_): *δ*_H_ ppm 7.09–7.14 (m, 3H, 3″,4″,5″-H), 7.18–7.22 (m, 2H, 2″,6″-H), 7.41 (t, *J* = 7.31 Hz, m, 1H, 4′-H), 7.50–7.53 (m, 1H, 5‴-H), 7.54–7.57 (m, 2H, 3′,5′-H), 7.78–7.81 (m, 1H, 4‴-H), 7.92–7.95 (m, 2H, 2′,6′-H), 8.00 (dd, *J* = 7.8, 1.5 Hz, 1H, 6‴-H), 8.12 (d, *J* = 8.1 Hz, 1H, 3‴-H), 9.24 (s, 1H, 5-H), 9.65 (s, 1H, CHN). ^13^C NMR (176 MHz, DMSO-*d*_6_): *δ*_C_ ppm 104.8 (C-4), 119.5 (C-2′,6′), 119.9 (C-3‴), 123.4 (C-1‴), 127.9 (C-3″,5″), 128.0 (C-4″), 128.3 (C-4′), 129.2 (C-5‴), 130.1 (C-3′,5′), 130.6 (C-2″,6″), 131.0 (C-6‴), 132.7 (C-5), 135.1 (C-4‴), 138.8 (C-1′), 140.8 (C-2‴), 144.4 (C-1″), 156.6 (CHN), 162.0 (COO), 162.8 (C-3). ^15^N NMR (71 MHz, DMSO-*d*_6_): *δ*_N_ ppm −117.9 (Pz N-2), −168.9 (Pz N-1), −194.3 (N^+^). ^11^B NMR (128 MHz, DMSO-*d*_6_): *δ*_B_ ppm 6.1 (B). HRMS (ESI^+^) for C_23_H_16_BN_3_NaO_3_ ([M + Na]^+^) calcd 416.1181, found 416.1183.

**[2-({[3-(hydroxy-κ*O*)-1-phenyl-1*H*-pyrazol-4-yl]methylidene}amino-κ*N*)benzoato(2-)-κ*O*](4-methylphenyl)boron (4b).** Yellow solid; yield 63% (257 mg); m.p. 333–334 °C. IR (v_max_, cm^−1^): 3126, 3069 (CH_arom._), 1693, 1631, 1588, 1494, 1405 (C=O, C=N, C=C), 1164, 1021 (C–O), 754, 730, 683 (C=C of benzene). ^1^H NMR (700 MHz, DMSO-*d*_6_): *δ*_H_ ppm 2.04 (s, 3H, 4″-CH_3_), 6.83 (d, *J* = 7.8 Hz, 2H, 3″,5″-H), 6.97 (d, *J* = 7.8 Hz, 2H, 2″,6″-H), 7.31–7.35 (m, 1H, 4′-H), 7.43–7.49 (m, 3H, 3′,5′,5‴-H), 7.69–7.74 (m, 1H, 4‴-H), 7.87–7.89 (m, 3H, 2′,6′,6‴-H), 8.15 (d, *J* = 8.2 Hz, 1H, 3‴-H), 9.35 (s, 1H, 5-H), 9.86 (s, 1H, CHN). ^13^C NMR (176 MHz, DMSO-*d*_6_): *δ*_C_ ppm 21.1 (4″-CH_3_), 104.6 (C-4), 119.2 (C-2′,6′), 119.8 (C-3‴), 123.0 (C-1‴), 128.1 (C-4′), 128.4 (C-3″,5″), 129.0 (C-5‴), 130.0 (C-3′,5′), 130.4 (C-2″,6″), 130.7 (C-6‴), 133.1 (C-5), 135.0 (C-4‴), 136.7 (C-4″), 138.6 (C-1′), 140.5 (C-2‴), 140.8 (C-1″), 156.6 (CHN), 161.9 (COO), 162.6 (C-3). ^15^N NMR (71 MHz, DMSO-*d*_6_): *δ*_N_ ppm −117.8 (Pz N-2), −168.7 (Pz N-1), −193.8 (N^+^). ^11^B NMR (128 MHz, DMSO-*d*_6_): *δ*_B_ ppm 6.2 (B). HRMS (ESI^+^) for C_24_H_18_BN_3_NaO_3_ ([M + Na]^+^) calcd 430.1338, found 430.1338.

**[2-({[3-(hydroxy-κ*O*)-1-phenyl-1*H*-pyrazol-4-yl]methylidene}amino-κ*N*)benzoato(2-)-κ*O*](4-methoxyphenyl)boron (4c).** Yellow solid; yield 54% (220 mg); m.p. 323–324 °C. IR (v_max_, cm^−1^): 3129 (CH_arom._), 1695, 1631, 1601, 1583, 1497 (C=O, C=N, C=C), 1163, 1020 (C–O), 753, 732, 682 (C=C of benzene). ^1^H NMR (700 MHz, DMSO-*d*_6_): *δ*_H_ ppm 3.52 (s, 3H, 4″-OCH_3_) 6.57–7.62 (m, 2H, 3″,5″-H), 6.95–7.01 (m, 2H, 2″,6″-H), 7.30–7.35 (m, 1H, 4′-H), 7.44–7.48 (m, 3H, 3′,5′,5‴-H), 7.71–7.73 (m, 1H, 4‴-H), 7.87–7.89 (m, 3H, 2′,6′,6‴-H), 8.16 (d, *J* = 8.2 Hz, 1H, 3‴-H), 9.35 (s, 1H, 5-H), 9.86 (s, 1H, CHN). ^13^C NMR (176 MHz, DMSO-*d*_6_): *δ*_C_ ppm 55.1 (4″-OCH_3_), 104.6 (C-4), 113.3 (C-3″, 5″), 119.2 (C-2′,6′), 119.8 (C-3‴), 123.0 (C-1‴), 128.1 (C-4′), 129.0 (C-5‴), 130.0 (C-3′, 5′), 130.7 (C-6‴), 131.7 (C-2″,6″), 133.0 (C-5), 135.0 (C-4‴), 135.6 (C-1″), 138.6 (C-1′), 140.5 (C-2‴), 156.5 (CHN), 159.01 (C-4″), 161.9 (COO), 162.6 (C-3). ^15^N NMR (71 MHz, DMSO-*d*_6_): *δ*_N_ ppm −168.9 (Pz N-1), −193.8 (N^+^), Pz N-2 was not found. ^11^B NMR (128 MHz, DMSO-*d*_6_): *δ*_B_ ppm 6.3 (B). HRMS (ESI^+^) for C_24_H_18_BN_3_NaO_4_ ([M + Na]^+^) calcd 446.1287, found 446.1292.

**(4-fluorophenyl)[2-({[3-(hydroxy-κ*O*)-1-phenyl-1*H*-pyrazol-4-yl]methylidene}amino-κ*N*)benzoato(2-)-κ*O*]boron (4d).** Yellow solid; yield 47% (194 mg); m.p. 318–319 °C. IR (v_max_, cm^−1^): 3129, 3073 (CH_arom._), 1696, 1629, 1582, 1405 (C=O, C=N, C=C), 1171, 1027 (C–O), 759, 731, 687 (C=C of benzene). ^1^H NMR (700 MHz, DMSO-*d*_6_): *δ*_H_ ppm 6.92–6.96 (m, 2H, 3″,5″-H), 7.20–7.24 (m, 2H, 2″,6″-H), 7.39–7.43 (m, 1H, 4′-H), 7.5–7.53 (m, 1H, 5‴-H), 7.54–7.56 (m, 2H, 3′,5′-H), 7.78–7.81 (m, 1H, 4‴-H), 7.91–7.92 (m, 2H, 2′,6′-H), 8.01 (dd, *J* = 7.8, 1.5 Hz, 1H, 6‴-H), 8.08 (d, *J* = 8.2 Hz, 1H, 3‴-H), 9.20 (s, 1H, 5-H), 9.56 (s, 1H, CHN). ^13^C NMR (176 MHz, DMSO-*d*_6_): *δ*_C_ ppm 104.2 (C-4), 114.3 (d, *J* = 19.6 Hz, C-3″,5″), 119.1 (C-2′,6′), 119.4 (C-3‴), 122.9 (C-1‴), 127.9 (C-4′), 128.7 (C-5‴), 129.7 (C-3′,5′), 130.5 (C-6‴), 132.19 (C-5), 132.19 (d, *J* = 14.4 Hz, C-2″,6″), 134.7 (C-4‴), 138.4 (C-1′), 140.0 (C-1″), 140.2 (C-2‴), 156.3 (CHN), 161.4 (COO), 162.0 (d, *J* = 243.2 Hz, C-4″), 162.2 (C-3). ^15^N NMR (71 MHz, DMSO-*d*_6_): *δ*_N_ ppm −117.3 (Pz N-2), −168.1 (Pz N-1), −194.3 (N^+^). ^19^F NMR (376 MHz, DMSO-*d*_6_): *δ*_N_ ppm −114.5 (F). ^11^B NMR (128 MHz, DMSO-*d*_6_): *δ*_B_ ppm 5.8 (B). HRMS (ESI^+^) for C_23_H_15_BFN_3_NaO_3_ ([M + Na]^+^) calcd 434.1087, found 434.1085.

**[2-({[3-(hydroxy-κ*O*)-1-phenyl-1*H*-pyrazol-4-yl]methylidene}amino-κ*N*)benzoato(2-)-κ*O*][4-(trifluoromethyl)phenyl]boron (4e).** Yellow solid; yield 58% (267 mg); m.p. 323–324 °C. IR (v_max_, cm^−1^): 3131, 3075 (CH_arom._), 1696, 1627, 1583, 1499, 1407 (C=O, C=N, C=C), 1326, 1295, 1174, 1108 (C–O, C–F), 802, 756, 730, 683 (C=C of benzene). ^1^H NMR (700 MHz, DMSO-*d*_6_): *δ*_H_ ppm 7.31–7.34 (m, 3H, 4′,2″,6″-H), 7.38–7.39 (m, 2H, 3″,5″-H), 7.44–7.49 (m, 3H, 3′,5′,5‴-H), 7.72–7.76 (m, 1H, 4‴-H), 7.86–7.90 (m, 2H, 2′,6′,6‴-H), 8.21 (d, *J* = 8.2 Hz, 1H, 3‴-H), 9.41 (s, 1H, 5-H), 9.91 (s, 1H, CHN). ^13^C NMR (176 MHz, DMSO-*d*_6_): *δ*_C_ ppm 104.5 (C-4), 119.3 (C-2′,6′), 120.0 (C-3‴), 122.3 (C-1‴), 124.4 (q, *J* = 3.9 Hz, C-3″,5″), 124.6 (q, *J* = 272.3 Hz, 4″-CF_3_), 128.1 (C-4′), 128.3 (q, *J* = 31.4 Hz, C-4″), 129.1 (C-5‴), 130.0 (C-3′,5′), 130.7 (C-6‴), 131.1 (C-2″,6″), 133.3 (C-5), 135.2 (C-4‴), 138.5 (C-1′), 140.3 (C-2‴), 149.3 (C-1″), 157.4 (CHN), 161.6 (COO), 162.3 (C-3). ^15^N NMR (71 MHz, DMSO-*d*_6_): *δ*_N_ ppm −117.6 (Pz N-2), −168.2 (Pz N-1), −196.9 (N^+^). ^19^F NMR (376 MHz, DMSO-*d*_6_): *δ*_N_ ppm −61.0 (CF_3_). ^11^B NMR (128 MHz, DMSO-*d*_6_): *δ*_B_ ppm 5.6 (B). HRMS (ESI^+^) for C_24_H_15_BF_3_N_3_NaO_3_ ([M + Na]^+^) calcd 484.1055, found 484.1059.

**[2-({[3-(hydroxy-κ*O*)-1-phenyl-1*H*-pyrazol-4-yl]methylidene}amino-κ*N*)-5-methylbenzoato(2-)-κ*O*](phenyl)boron (4f).** Yellow solid; yield 50% (204 mg); m.p. 342–343 °C. IR (v_max_, cm^−1^): 3126, 3055 (CH_arom._), 1691, 1626, 1587, 1488, 1403 (C=O, C=N, C=C), 1175. 1021 (C–O), 864, 754, 732 (C=C of benzene). ^1^H NMR (700 MHz, DMSO-*d*_6_): *δ*_H_ ppm 2.33 (s, 3H, 5‴-CH_3_), 7.05–7.10 (m, 3H, 3″,4″,5″-H), 7.14–7.16 (m, 2H, 2″,6″-H), 7.35–7.39 (m, 1H, 4′-H), 7.49–7.53 (m, 2H, 3′,5′-H), 7.57 (dd, *J* = 8.5, 2.1 Hz, 1H, 4‴-H), 7.76 (d, *J* = 2.0 Hz, 1H, 6‴-H), 7.89–7.92 (m, 2H, 2′,6′-H), 8.03 (d, *J* = 8.8 Hz, 1H, 3‴-H), 9.27 (s, 1H, 5-H), 9.71 (s, 1H, CHN). ^13^C NMR (176 MHz, DMSO-*d*_6_): *δ*_C_ ppm 20.6 (5‴-CH_3_), 104.4 (C-4), 119.0 (C-2′,6′), 119.3 (C-3‴), 122.6 (C-1‴), 127.5 (C-3″,4″,5″), 127.8 (C-4′), 129.7 (C-3′,5′), 130.2 (C-2″,6″), 130.6 (C-6‴), 132.3 (C-5), 135.4 (C-4‴), 138.0 (C-2‴), 138.5 (C-1′), 138.8 (C-5‴), 144.0 (C-1″), 155.5 (CHN), 161.7 (COO), 162.3 (C-3). ^15^N NMR (71 MHz, DMSO-*d*_6_): *δ*_N_ ppm −117.5 (Pz N-2), −169.0 (Pz N-1), −193.4 (N^+^). ^11^B NMR (128 MHz, DMSO-*d*_6_): *δ*_B_ ppm 5.8 (B). HRMS (ESI^+^) for C_24_H_18_BN_3_NaO_3_ ([M + Na]^+^) calcd 430.1338, found 430.1340.

**[2-({[3-(hydroxy-κ*O*)-1-phenyl-1*H*-pyrazol-4-yl]methylidene}amino-κ*N*)-5-methylbenzoato(2-)-κ*O*](4-methylphenyl)boron (4g).** Yellow solid; yield 59% (249 mg); m.p. 339–340 °C. IR (v_max_, cm^−1^): 3086, 3039 (CH_arom._), 1698, 1624, 1589, 1488, 1404 (C=O, C=N, C=C), 1218, 1167, 1038 (C–O), 785, 752, 732, 684 (C=C of benzene). ^1^H NMR (700 MHz, DMSO-*d*_6_): *δ*_H_ ppm 2.04 (s, 3H, 4″-CH_3_), 2.28 (s, 3H, 5‴-CH_3_), 6.83 (d, *J* = 7.8 Hz, 2H, 3″,5″-H), 6.93–6.98 (m, 2H, 2″,6″-H), 7.29–7.35 (m, 1H, 4′-H), 7.42–7.49 (m, 2H, 3′,5′-H), 7.52 (dd, *J* = 8.7, 2.1 Hz, 1H, 4‴-H), 7.67 (d, *J* = 2.0 Hz, 1H, 6‴-H), 7.84–7.89 (m, 2H, 2′,6′-H), 8.02 (d, *J* = 8.4 Hz, 1H, 3‴-H), 9.32 (s, 1H, 5-H), 9.81 (s, 1H, CHN). ^13^C NMR (176 MHz, DMSO-*d*_6_): *δ*_C_ ppm 20.8 (5‴-CH_3_), 21.1 (4″-CH_3_), 104.5 (C-4), 119.2 (C-2′,6′), 119.6 (C-3‴), 122.7 (C-1‴), 128.0 (C-4′), 128.4 (C-3″,5″), 130.0 (C-3′,5′), 130.4 (C-2″,6″), 130.7 (C-6‴), 132.8 (C-5), 135.6 (C-4‴), 136.7 (C-4″), 138.2 (C-2‴), 138.6 (C-1′), 138.9 (C-5‴), 140.9 (C-1″), 155.8 (CHN), 162.0 (COO), 162.5 (C-3). ^15^N NMR (71 MHz, DMSO-*d*_6_): *δ*_N_ ppm −169.3 (Pz N-1), −193.2 (N^+^), Pz N-2 was not found. ^11^B NMR (128 MHz, DMSO-*d*_6_): *δ*_B_ ppm 6.3 (B). HRMS (ESI^+^) for C_25_H_20_BN_3_NaO_3_ ([M + Na]^+^) calcd 444.1494, found 444.1495.

**[2-({[3-(hydroxy-κ*O*)-1-phenyl-1*H*-pyrazol-4-yl]methylidene}amino-κ*N*)-5-methylbenzoato(2-)-κ*O*](4-methoxyphenyl)boron (4h).** Yellow solid; yield 56% (245 mg); m.p. 330–331 °C. IR (v_max_, cm^−1^): 3126, 3037 (CH_arom._), 1694, 1631, 1588, 1494, 1406 (C=O, C=N, C=C), 1165, 1021 (C–O), 863, 795, 754, 730, 684 (C=C of benzene). ^1^H NMR (700 MHz, DMSO-*d*_6_): *δ*_H_ ppm 2.28 (s, 3H, 5‴-CH_3_), 3.52 (s, 3H, 4″-OCH_3_), 6.56–6.63 (m, 2H, 3″,5″-H), 6.95–7.01 (m, 2H, 2″,6″-H), 7.31–7.35 (m, 1H, 4′-H), 7.45–7.49 (m, 2H, 3′,5′-H), 7.53 (dd, *J* = 8.6, 2.0 Hz, 1H, 4‴-H), 7.68 (d, *J* = 2.0 Hz, 1H, 6‴-H), 7.84–7.88 (m, 2H, 2′,6′-H), 8.03 (d, *J* = 8.4 Hz, 1H, 3‴-H), 9.32 (s, 1H, 5-H), 9.81 (s, 1H, CHN). ^13^C NMR (176 MHz, DMSO-*d*_6_): *δ*_C_ ppm 20.8 (5‴-CH_3_), 55.1 (4″-OCH_3_), 104.5 (C-4), 113.3 (C-3″,5″), 119.2 (C-2′,6′), 119.6 (C-3‴), 122.7 (C-1‴), 128.0 (C-4′), 130.0 (C-3′,5′), 130.7 (C-6‴), 131.6 (C-2″,6″), 132.8 (C-5), 135.6 (C-4‴), 135.8 (C-1″), 138.2 (C-2‴), 138.6 (C-1′), 138.9 (C-5‴), 155.7 (CHN), 159.0 (C-4″), 162.0 (COO), 162.6 (C-3). ^15^N NMR (71 MHz, DMSO-*d*_6_): *δ*_N_ ppm −169.3 (Pz N-1), −192.9 (N^+^), Pz N-2 was not found. ^11^B NMR (128 MHz, DMSO-*d*_6_): *δ*_B_ ppm 5.9 (B). HRMS (ESI^+^) for C_25_H_20_BN_3_NaO_4_ ([M + Na]^+^) calcd 460.1444, found 460.1447.

**[5-chloro-2-({[3-(hydroxy-κ*O*)-1-phenyl-1*H*-pyrazol-4-yl]methylidene}amino-κ*N*)benzoato(2-)-κ*O*](phenyl)boron (4i).** Yellow solid; yield 71% (303 mg); m.p. 365–366 °C. IR (v_max_, cm^−1^): 3082 (CH_arom._), 1700, 1623, 1589, 1497, 1472, 1400 (C=O, C=N, C=C), 1179, 1021 (C–O), 776, 752, 688, 643 (C=C of benzene, C–Cl). ^1^H NMR (700 MHz, DMSO-*d*_6_): *δ*_H_ ppm 7.05–7.10 (m, 3H, 3″,4″,5″-H), 7.12–7.16 (m, 2H, 2″,6″-H), 7.35–7.39 (m, 1H, 4′-H), 7.48–7.53 (m, 2H, 3′,5′-H), 7.83–7.87 (m, 2H, 4‴,6‴-H), 7.89–7.93 (m, 2H, 2′,6′-H), 8.25–8.28 (m, 1H, 3‴-H), 9.37 (s, 1H, 5-H), 9.82 (s, 1H, CHN). ^13^C NMR (176 MHz, DMSO-*d*_6_): *δ*_C_ ppm 104.5 (C-4), 119.2 (C-2′,6′), 122.1 (C-3‴), 124.4 (C-1‴), 127.7 (C-3″,5″), 127.8 (C-4″), 128.0 (C-4′), 129.7 (C-6‴), 129.8 (C-3′,5′), 130.3 (C-2″,6″), 132.99 (C-5), 133.01 (C-5‴), 134.6 (C-4‴), 138.4 (C-1′), 139.4 (C-2‴), 143.6 (C-1″), 157.1 (CHN), 160.6 (COO), 162.4 (C-3). ^15^N NMR (71 MHz, DMSO-*d*_6_): *δ*_N_ ppm −117.4 (Pz N-2), −168.0 (Pz N-1), −197.5 (N^+^). ^11^B NMR (128 MHz, DMSO-*d*_6_): *δ*_B_ ppm 5.9 (B). HRMS (ESI^+^) for C_23_H_15_BClN_3_NaO_3_ ([M + Na]^+^) calcd 450.0791, found 450.0794.

**[5-chloro-2-({[3-(hydroxy-κ*O*)-1-phenyl-1*H*-pyrazol-4-yl]methylidene}amino-κ*N*)benzoato(2-)-κ*O*](4-methylphenyl)boron (4j).** Yellow solid; yield 55% (243 mg); m.p. 364–365 °C. IR (v_max_, cm^−1^): 3105, 3085 (CH_arom._), 1701, 1617, 1587, 1497, 1469, 1400 (C=O, C=N, C=C), 1206, 1172, 1035 (C–O), 856, 772, 748, 679 (C=C of benzene, C–Cl). ^1^H NMR (700 MHz, DMSO-*d*_6_): *δ*_H_ ppm 2.05 (s, 3H, 4″-CH_3_), 6.84 (d, *J* = 7.8 Hz, 2H, 3″,5″-H), 6.97 (d, *J* = 7.9 Hz, 2H, 2″,6″-H), 7.32–7.36 (m, 1H, 4′-H), 7.45–7.49 (m, 2H, 3′,5′-H), 7.79 (d, 1H, *J* = 2.5 Hz, 6‴-H), 7.81 (dd, *J* = 8.6, 2.6 Hz, 1H, 4‴-H), 7.86–7.90 (m, 2H, 2′,6′-H), 8.25 (d, *J* = 8.7 Hz, 1H, 3‴-H), 9.39 (s, 1H, 5-H), 9.85 (s, 1H, CHN). ^13^C NMR (176 MHz, DMSO-*d*_6_): *δ*_C_ ppm 21.1 (4″-CH_3_), 104.6 (C-4), 119.3 (C-2′,6′), 122.3 (C-3‴), 124.5 (C-1‴), 128.2 (C-4′), 128.4 (C-3″,5″), 129.7 (C-6‴), 130.0 (C-3′,5′), 130.4 (C-2″,6″), 133.0 (C-5), 133.3 (C-5‴), 134.7 (C-4‴), 136.9 (C-4″), 138.5 (C-1′), 139.5 (C-2‴), 140.7 (C-1″), 157.2 (CHN), 160.8 (COO), 162.5 (C-3). ^15^N NMR (71 MHz, DMSO-*d*_6_): *δ*_N_ ppm, −168.6 (Pz N-1), −197.9 (N^+^), Pz N-2 was not found. ^11^B NMR (128 MHz, DMSO-*d*_6_): *δ*_B_ ppm 6.5 (B). HRMS (ESI^+^) for C_24_H_17_BClN_3_NaO_4_ ([M + Na]^+^) calcd 464.0948, found 464.0952.

**[5-chloro-2-({[3-(hydroxy-κ*O*)-1-phenyl-1*H*-pyrazol-4-yl]methylidene}amino-κ*N*)benzoato(2-)-κ*O*](4-methoxyphenyl)boron (4k).** Yellow solid; yield 48% (220 mg); m.p. 333–334 °C. IR (v_max_, cm^−1^): 3128, 3040 (CH_arom._), 1694, 1633, 1591, 1477, 1405(C=O, C=N, C=C), 1182, 1010 (C–O), 861, 754, 728, 669 (C=C of benzene, C–Cl). ^1^H NMR (700 MHz, DMSO-*d*_6_): *δ*_H_ ppm 3.55 (s, 3H, 4″-OCH_3_), 6.58–6.66 (m, 2H, 3″,5″-H), 7.98–7.04 (m, 2H, 2″,6″-H), 7.34–7.36 (m, 1H, 4′-H), 7.46–7.53 (m, 2H, 3′,5′-H), 7.81–7.86 (m, 2H, 4‴,6‴-H), 7.88–7.92 (m, 2H, 2′,6′-H), 8.23–8.27 (m, 1H, 3‴-H), 9.37 (s, 1H, 5-H), 9.83 (s, 1H, CHN). ^13^C NMR (176 MHz, DMSO-*d*_6_): *δ*_C_ ppm 55.0 (4″-OCH_3_), 104.6 (C-4), 113.3 (C-3″,5″), 119.2 (C-2′,6′), 122.2 (C-3‴), 124.5 (C-1‴), 128.1 (C-4′), 129.7 (C-6‴), 129.9 (C-3′,5′), 131.7 (C-2″,6″), 133.0 (C-5), 133.1 (C-5‴), 134.7 (C-4‴), 135.2 (C-1″), 138.5 (C-1′), 139.5 (C-2‴), 156.9 (CHN), 159.1 (C-4″), 160.8 (COO), 162.5 (C-3). ^15^N NMR (71 MHz, DMSO-*d*_6_): *δ*_N_ ppm −117.8 (Pz N-2), −168.0 (Pz N-1), −196.8 (N^+^). ^11^B NMR (128 MHz, DMSO-*d*_6_): *δ*_B_ ppm 6.2 (B). HRMS (ESI^+^) for C_24_H_17_BClN_3_NaO_4_ ([M + Na]^+^) calcd 480.0897, found 480.0898.

## 4. Conclusions

In conclusion, a series of novel pyrazole-containing boron (III) complexes were synthesized from 3-hydroxy-1-phenyl-1*H*-pyrazole-4-carbaldehyde, 2-aminobenzene-carboxylic acids, and boronic acids via a one-pot multicomponent reaction, achieving good yields. The novel compounds were characterized via IR and advanced NMR spectroscopies as well as HRMS data. Additionally, X-ray single crystal analysis showed the presence of an asymmetric boron center and two enantiomers in each unit cell of crystals. The photophysical properties of the obtained compounds were investigated in different solvents. The compounds were faintly luminescent in THF, MeOH, ACN, and DMF solutions, and demonstrated aggregation-induced emission enhancement in mixed THF–water solutions. These compounds can be further investigated as luminescent materials or probes.

## Figures and Tables

**Table 1:** Absorption (λ_abs_ of absorption maxima and ε), fluorescence emission (*λ*_em_), and quantum yield (*Φ_f_*) parameters and Stokes shifts for **4a**–**k** in different solvents: ^a^ THF, ^b^ MeOH, ^c^ ACN, and ^d^ DMF (λ_ex_ = 400 nm) and mixed THF–water solutions (λ_ex_ = 440 nm).

Entry	Comp.	λ_abs_ (nm)	ε × 10^3^ (dm^3^mol^−1^ cm^−1^)	λ_em_ (nm)	Stokes Shifts (nm)	Φ_f_ (%)	λ_em_ (nm) in H_2_O–THF (*v*/*v* %)	Φ_f_ (%) in H_2_O–THF (*v*/*v* %)
**1**	**4a ^a^**	260	14.68		133	2.7	524 H_2_O/THF (80/20)	26.2 H_2_O/THF (80/20)
		310	10.61	533				
		400	17.80					
**2**	**4b ^a^**	260	26.07		132	2.8	526 H_2_O/THF (70/30)	18.8 H_2_O/THF (70/30)
		311	30.14	531				
		399	30.72					
**3**	**4c ^a^**	260	12.02		135	0.2	529 H_2_O/THF (90/10)	11.9 H_2_O/THF (90/10)
		309	7.69	533				
		398	13.17					
**4**	**4d ^a^**	259	18.56		137	1.9	544 H_2_O/THF (80/20)	25.4 H_2_O/THF (80/20)
		312	13.12	534				
		397	22.54					
**5**	**4e ^a^**	260	20.58				525 H_2_O/THF (80/20)	30.6 H_2_O/THF (80/20)
		312	13.68	532	135	1.9		
		397	24.65					
**6**	**4f ^a^**	261	11.67				527 H_2_O/THF (80/20)	15.1 H_2_O/THF (80/20)
		312	7.45	529	128	0.4		
		401	14.71					
**7**	**4g ^a^**	260	6.67				546 H_2_O/THF (80/20)	52.2 H_2_O/THF (80/20)
		313	4.52	543	142	2.0		
		401	8.11					
**8**	**4h ^a^**	262	13.01				538 H_2_O/THF (80/20)	29.2 H_2_O/THF (80/20)
		313	7.80	531	131	0.5		
		400	15.00					
**9**	**4i ^a^**	258	6.10				537 H_2_O/THF (80/20)	7.0 H_2_O/THF (80/20)
		315	4.60	538	134	3.7		
		404	7.88					
**10**	**4j ^a^**	257	11.53				536 H_2_O/THF (80/20)	24.4 H_2_O/THF (80/20)
		315	8.82	539	135	4.3		
		404	14.43					
**11**	**4k ^a^**	259	20.80		135	0.1	539 H_2_O/THF (80/20)	19.8 H_2_O/THF (80/20)
		315	15.48	538				
		403	26.77					
**12**	**4a ^b^**	255	13.46					
		305	7.86	531	141	1.6	-	-
		390	14.10					
**13**	**4a ^c^**	257	20.00					
		306	14.03	531	140	3.1	-	-
		391	25.17					
**14**	**4a ^d^**	258	26.45					
		305	17.53	531	138	1.5	-	-
		393	28.78					

## Data Availability

Data are contained within the article and Supplementary Materials.

## Supplementary Materials

The following supporting information can be downloaded at: https://www.mdpi.com/article/10.3390/molecules29143432/s1, Figures S1–S10: Fluorescence emission spectra of compounds **4b**–**4k** in mixed THF–water solutions. Figures S11–S56: ^1^H, ^13^C, ^19^F ^11^B NMR and HRMS (ESI) spectra of compounds **4a**–**k**.
