# Supplementary material for: Multicomponent Synthesis of New Fluorescent Boron Complexes Derived from 3-Hydroxy-1-phenyl-1H-pyrazole-4-carbaldehyde
Source: Molecules. 2024 Jul 22;29(14):3432. doi: 10.3390/molecules29143432 (PMC11279739; doi:10.3390/molecules29143432)
Supplement: Supplementary file 1 [file molecules-29-03432-s001.zip › molecules-3105219-supplementary.pdf]

Supplementary materials

Multicomponent Synthesis of New Fluorescent Boron Complexes Derived from 3-Hydroxy-1-phenyl-1H-pyrazole-4-carbaldehyde

Viktorija Savickienė [1], Aurimas Bieliauskas [2], Sergey Belyakov [3], Eglė Arbačiauskienė [1,*] and Algirdas Šačkus [2,*]

1 Department of Organic Chemistry, Kaunas University of Technology, Radvilėnų pl. 19, LT-50254 Kaunas, Lithuania; viktorija.dargyte@ktu.lt
2 Institute of Synthetic Chemistry, Kaunas University of Technology, K. Baršausko g. 59, LT-51423 Kaunas, Lithuania; aurimas.bieliauskas@ktu.lt
3 Latvian Institute of Organic Synthesis, Aizkraukles 21, LV-1006 Rīga, Latvia; serg@osi.lv
* Correspondence: egle.arbaciauskiene@ktu.lt (E.A.); algirdas.sackus@ktu.lt (A.Š.)

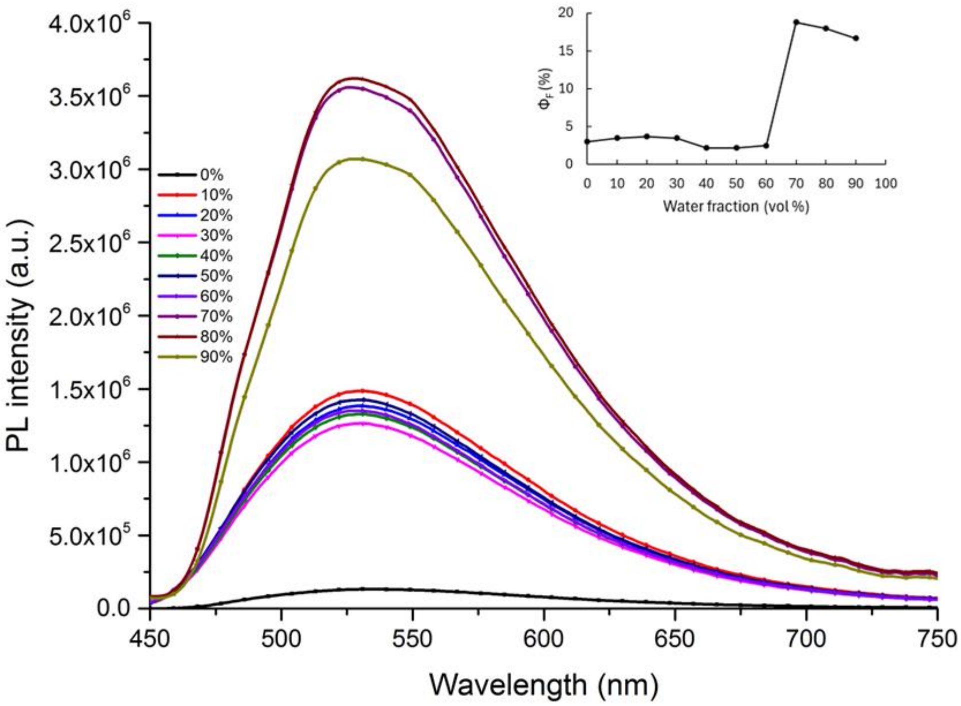

Figure S1. Fluorescence emission spectra of compound **4b** in mixed THF–water solutions. The insert shows the relationship between the fluorescence quantum yield and the water fraction.

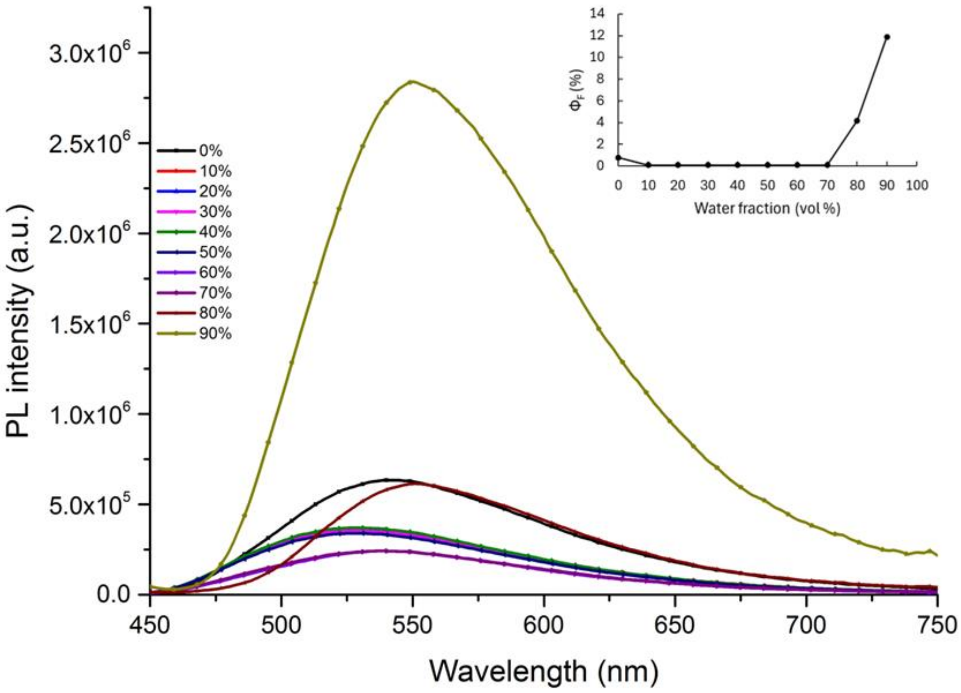

Figure S2. Fluorescence emission spectra of compound **4c** in mixed THF–water solutions. The insert shows the relationship between the fluorescence quantum yield and the water fraction.

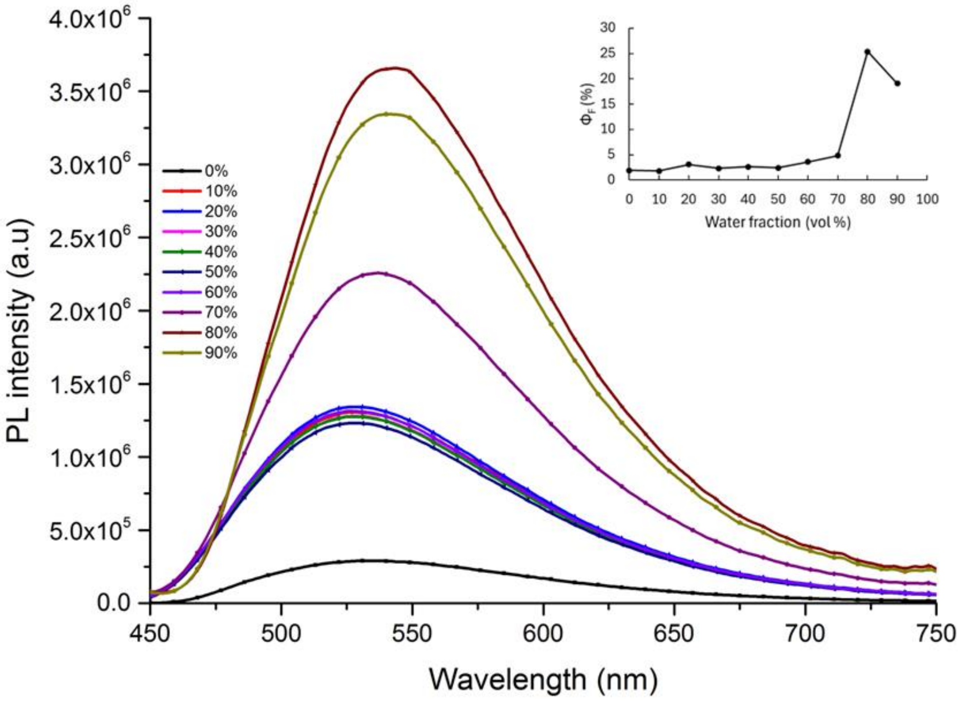

Figure S3. Fluorescence emission spectra of compound **4d** in mixed THF–water solutions. The insert shows the relationship between the fluorescence quantum yield and the water fraction.

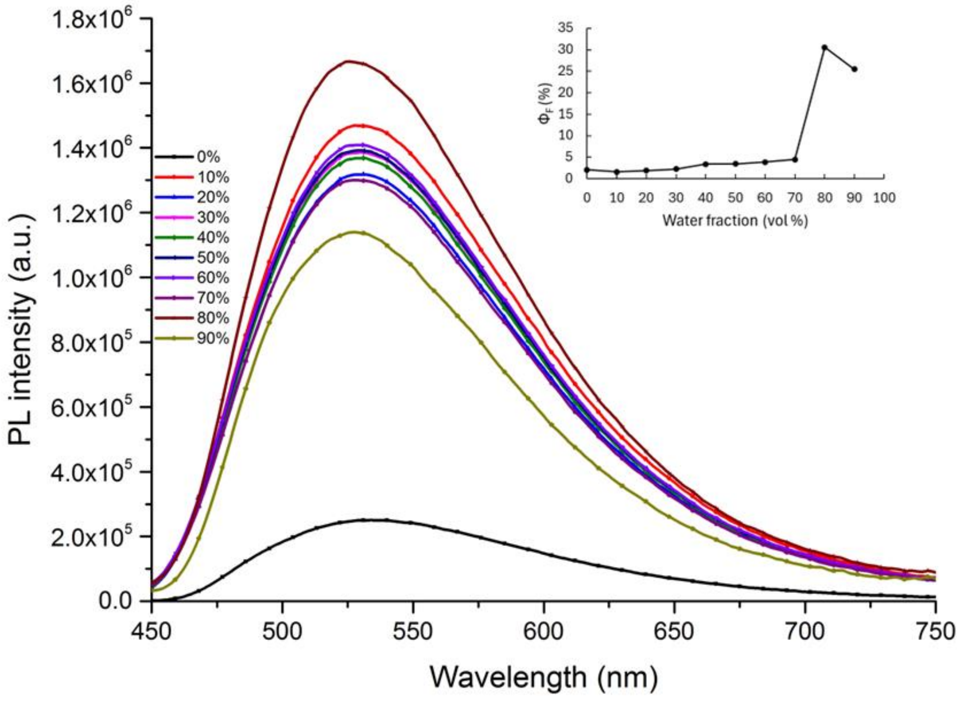

Figure S4. Fluorescence emission spectra of compound **4e** in mixed THF–water solutions. The insert shows the relationship between the fluorescence quantum yield and the water fraction.

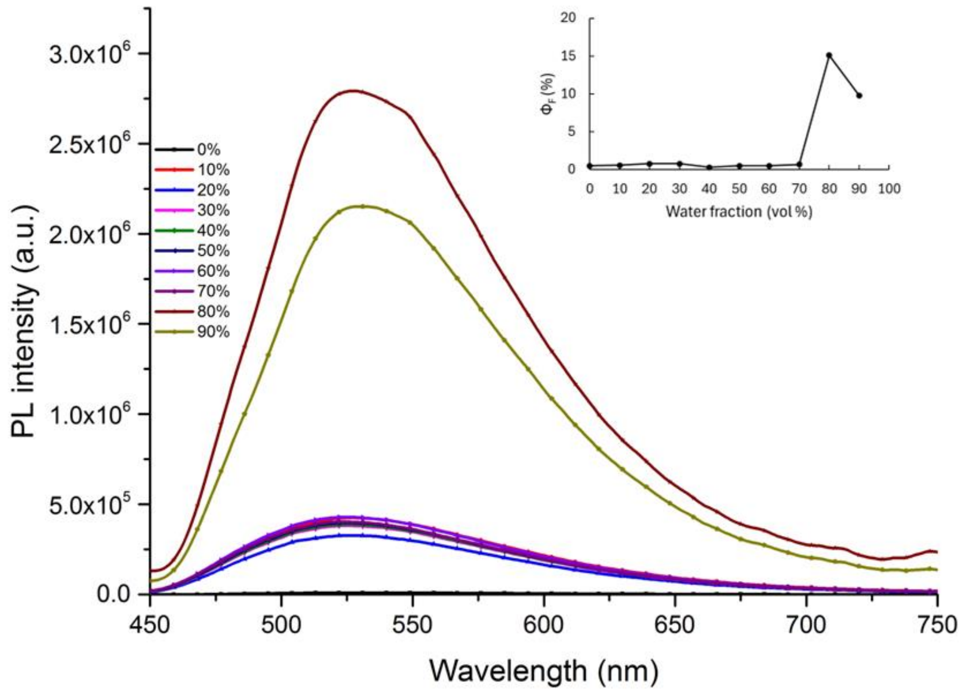

Figure S5. Fluorescence emission spectra of compound **4f** in mixed THF–water solutions. The insert shows the relationship between the fluorescence quantum yield and the water fraction.

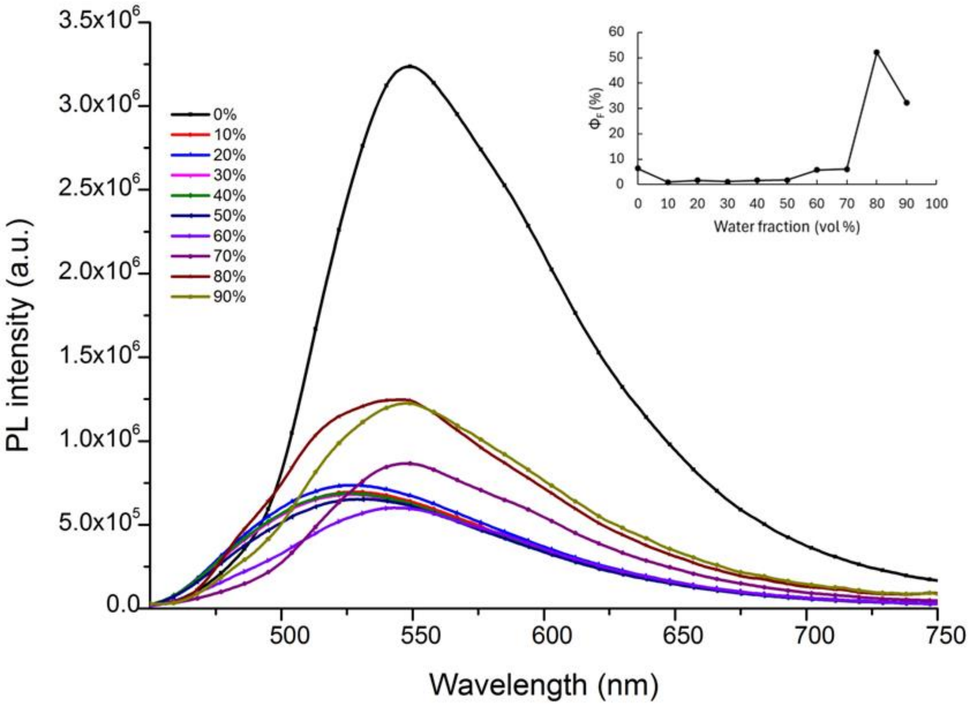

Figure S6. Fluorescence emission spectra of compound **4g** in mixed THF–water solutions. The insert shows the relationship between the fluorescence quantum yield and the water fraction.

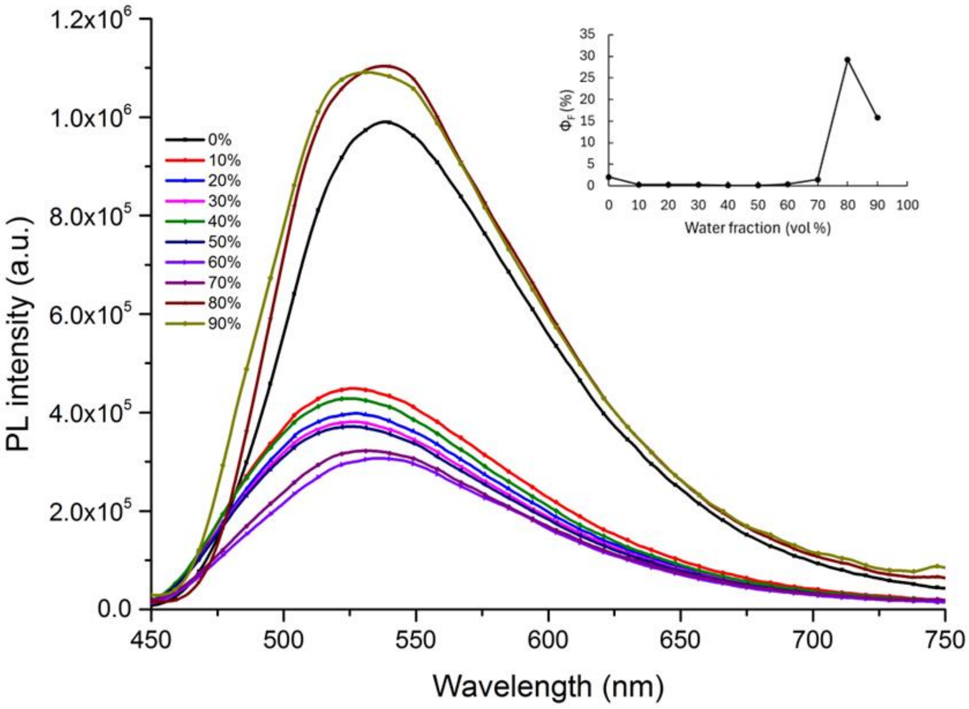

Figure S7. Fluorescence emission spectra of compound **4h** in mixed THF−water solutions. The insert shows the relationship between the fluorescence quantum yield and the water fraction.

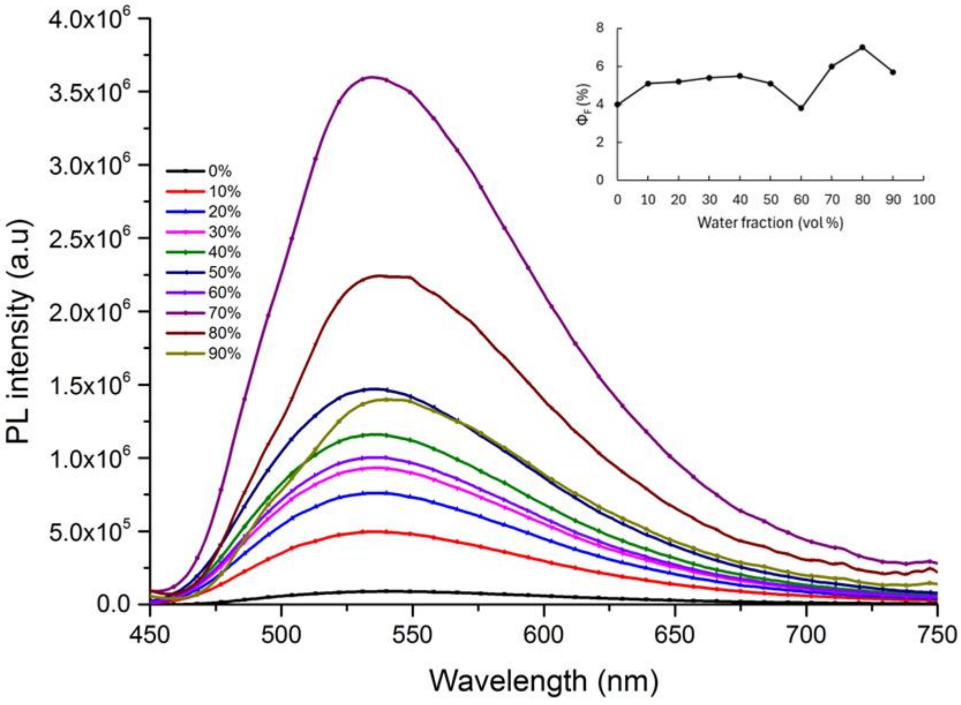

Figure S8. Fluorescence emission spectra of compound **4i** in mixed THF−water solutions. The insert shows the relationship between the fluorescence quantum yield and the water fraction

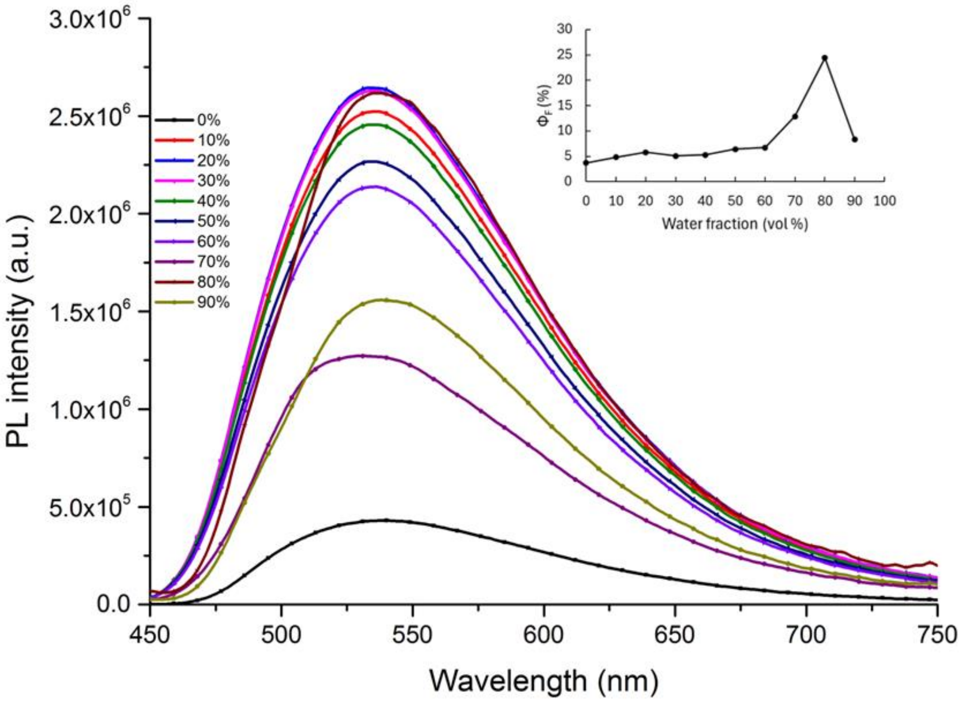

Figure S9. Fluorescence emission spectra of compound **4j** in mixed THF–water solutions. The insert shows the relationship between the fluorescence quantum yield and the water fraction.

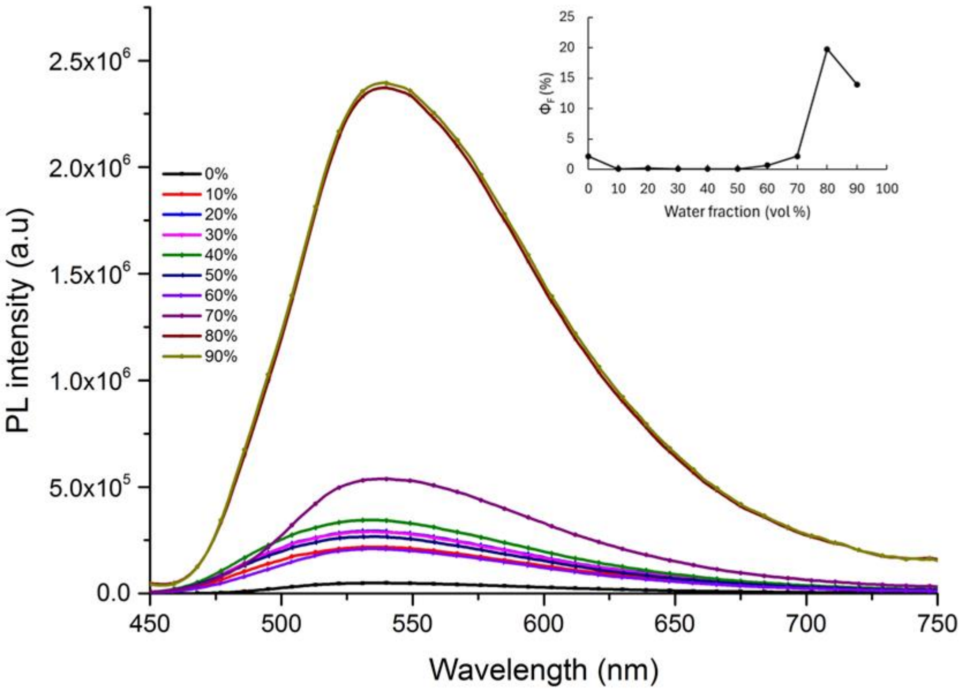

Figure S10. Fluorescence emission spectra of compound **4k** in mixed THF–water solutions. The insert shows the relationship between the fluorescence quantum yield and the water fraction.

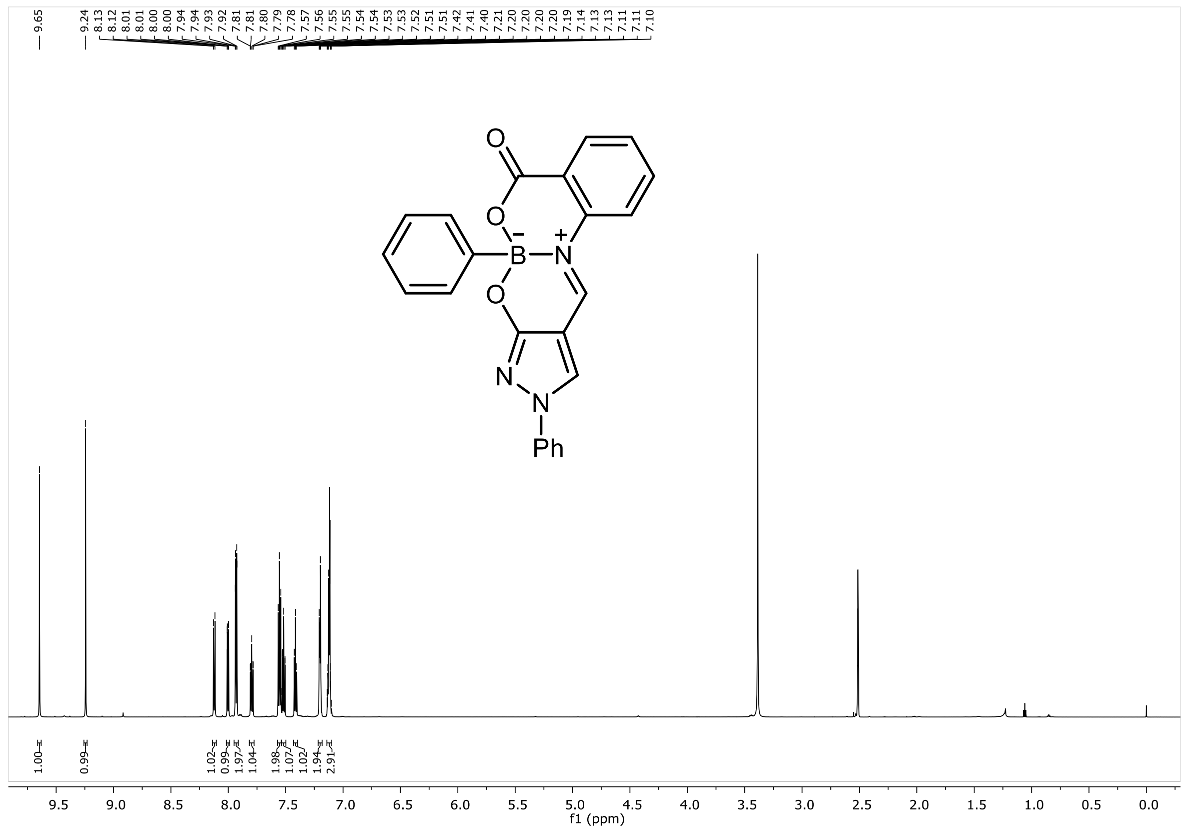

Figure S11. ^{1}H NMR (700 MHz, DMSO-d_6) spectrum of **4a**

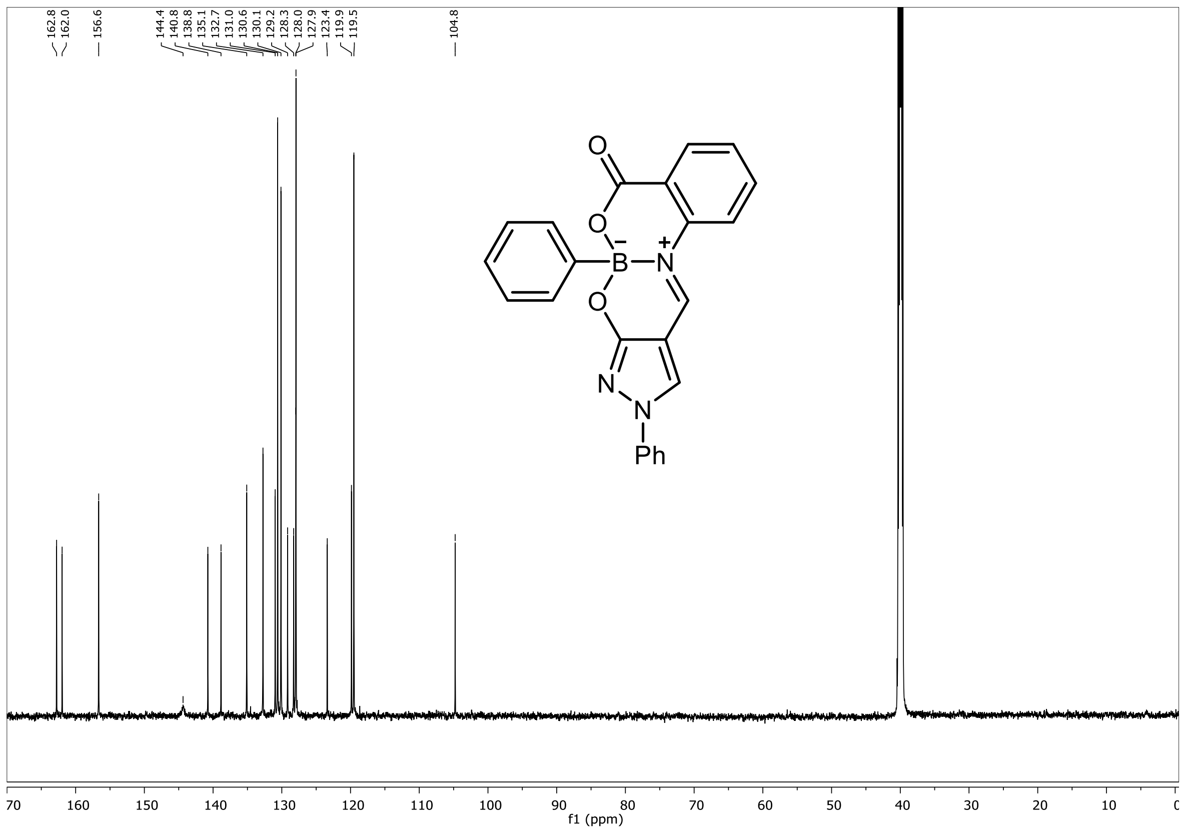

Figure S12. ^{13}C NMR (176 MHz, DMSO-d_6) spectrum of **4a**

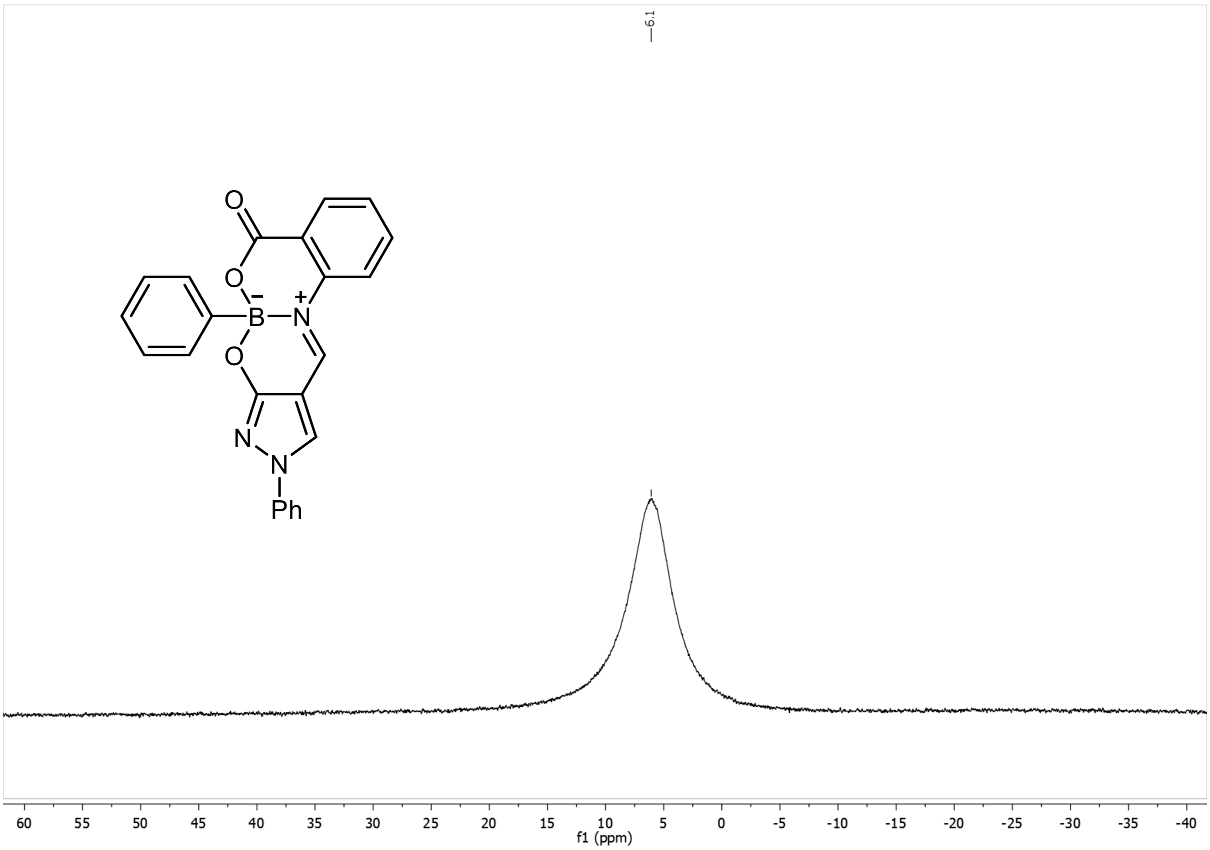

Figure S13. ^{11}B NMR (128 MHz, DMSO-d_6) spectrum of **4a**

Compound Spectrum SmartFormula Report

Analysis Info

Analysis Name	D:\Data\VDD-224_Boron.d	Acquisition Date	3/28/2023 8:46:51 PM
Method	DirectInfusion_TuneLow_pos.m	Operator	hplc
Sample Name	VDD-224_Boron	Instrument	micrOTOF-Q III 8228888.20448
Comment	AB		

Acquisition Parameter

Source Type	ESI	Ion Polarity	Positive	Set Nebulizer	0.4 Bar
Focus	Not active	Set Capillary	4500 V	Set Dry Heater	180 °C
Scan Begin	50 m/z	Set End Plate Offset	-500 V	Set Dry Gas	4.0 l/min
Scan End	1000 m/z	Set Collision Cell RF	140.0 Vpp	Set Divert Valve	Waste

#	RT [min]	Area	Int. Type	I	S/N	Chromatogram	Max. m/z	FWHM [min]
n.a.	15.6	n.a.	Single spectrum	n.a.	n.a.	n.a.	318.2396	n.a.

+MS, 15.6min #933

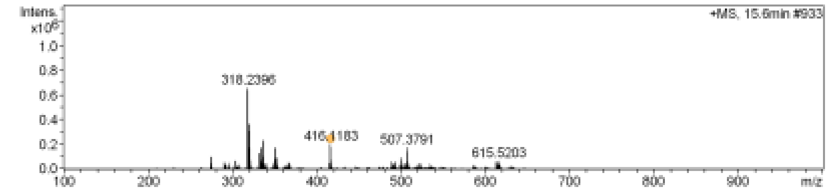

Meas. m/z	#	Ion Formula	m/z	err [ppm]	mSigma	# Sigma	Score	rdb	e⁻ Conf	N-Rule
416.1183	1	C23H16BN3NaO3	416.1181	0.5	4.6	1	100.00	17.5	even	ok

Figure S14. HRMS (ESI^+) report of **4a**

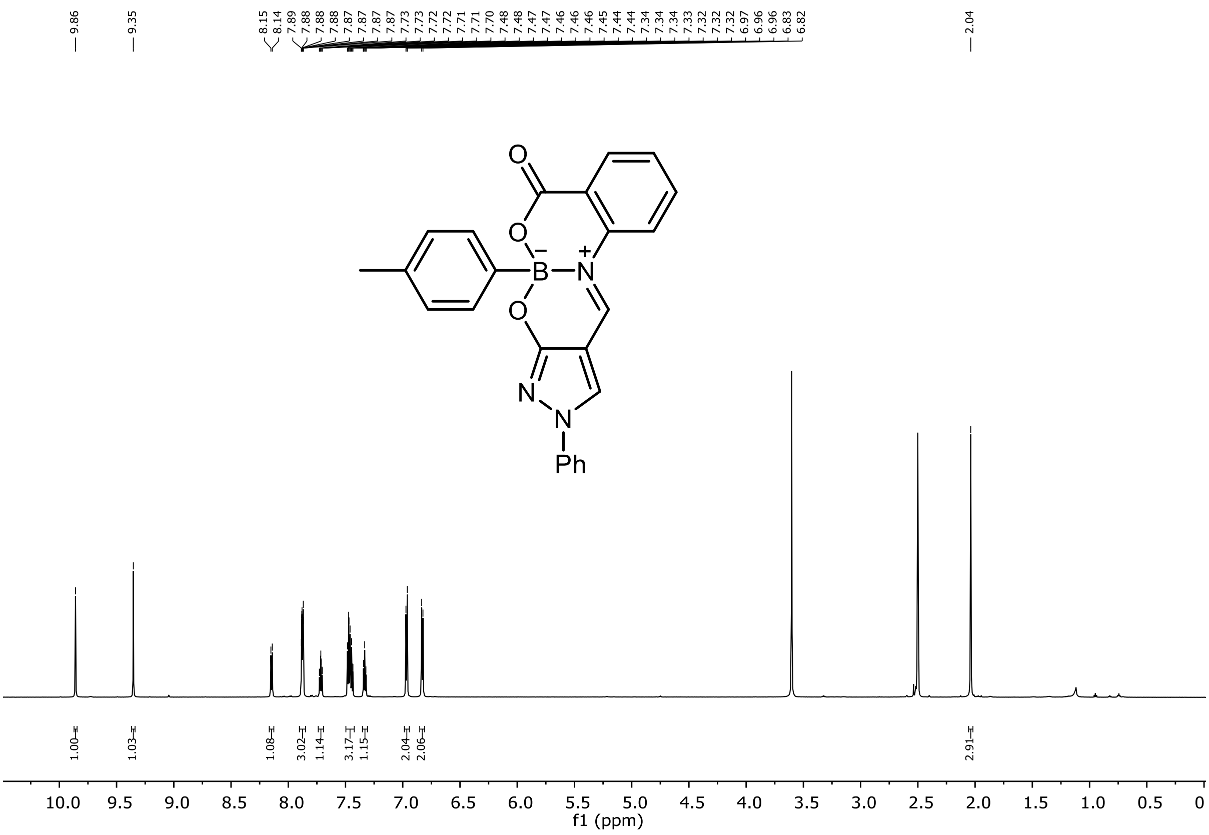

Figure S15. ^{1}H NMR (700 MHz, DMSO-d_6) spectrum of **4b**

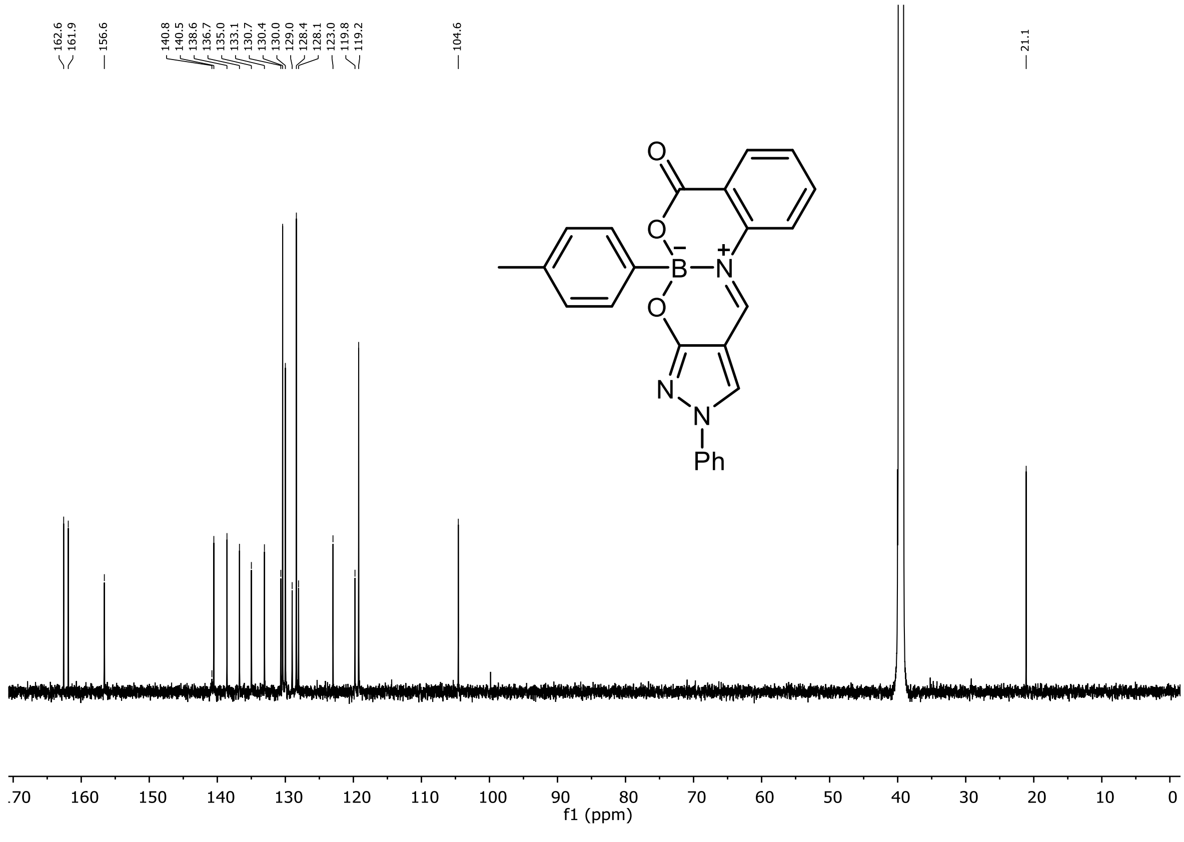

Figure S16. ^{13}C NMR (176 MHz, DMSO-d_6) spectrum of **4b**

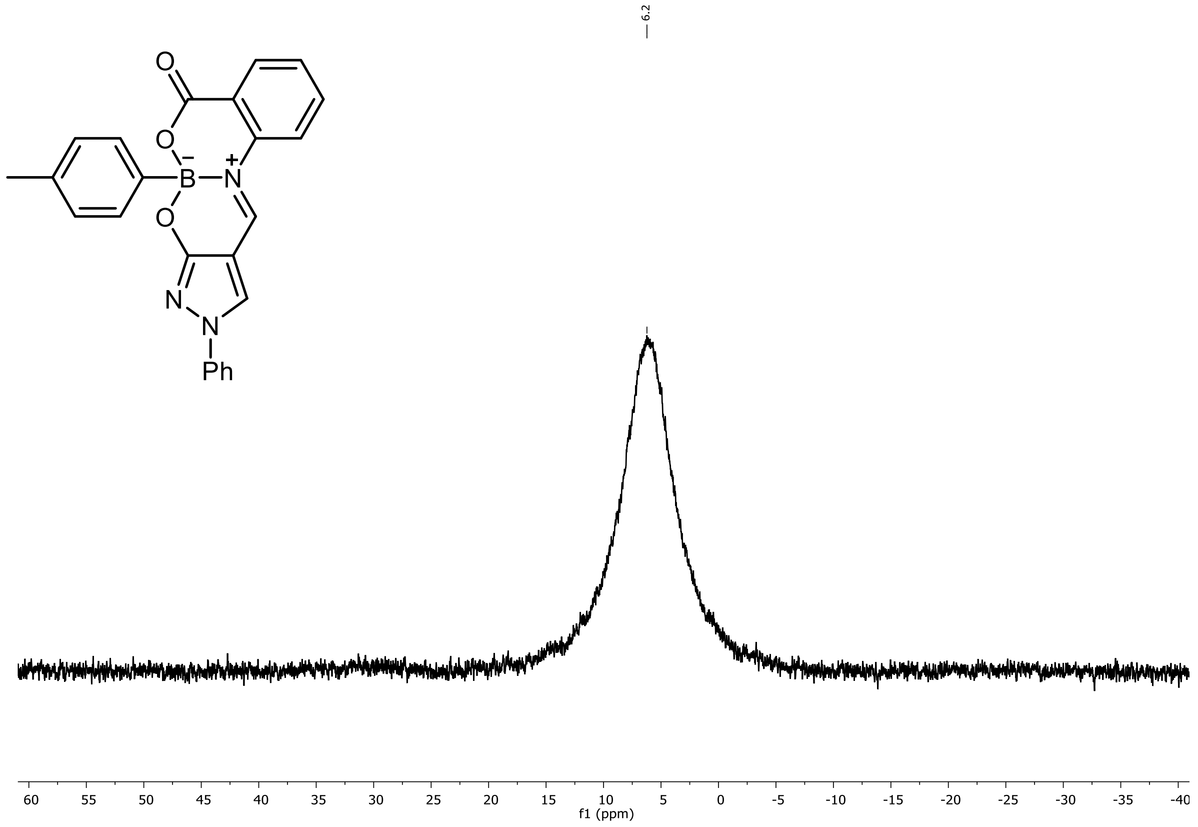

Figure S17. ^{11}B NMR (128 MHz, DMSO-d_6) spectrum of **4b**

Compound Spectrum SmartFormula Report

Analysis Info

Analysis Name	D:\Data\VDD-228.d	Acquisition Date	3/1/2024 7:24:27 PM
Method	DirectInfusion_TuneLow_pos.m	Operator	hplc
Sample Name	VDD-228	Instrument	micrOTOF-Q III 8228888.20448
Comment	AB		

Acquisition Parameter

Source Type	ESI	Ion Polarity	Positive	Set Nebulizer	0.4 Bar
Focus	Not active	Set Capillary	4500 V	Set Dry Heater	180 °C
Scan Begin	50 m/z	Set End Plate Offset	-500 V	Set Dry Gas	4.0 l/min
Scan End	1000 m/z	Set Collision Cell RF	140.0 Vpp	Set Divert Valve	Waste

#	RT [min]	Area	Int. Type	I	S/N	Chromatogram	Max. m/z	FWHM [min]
n.a.	3.1	n.a.	Single spectrum	n.a.	n.a.	n.a.	430.1338	n.a.

+MS, 3.1min #185

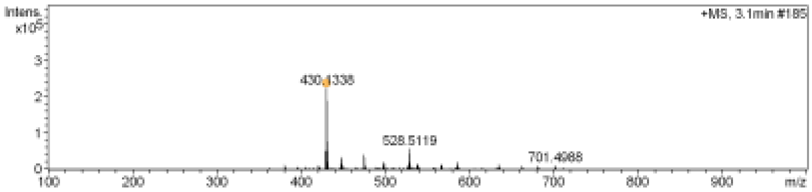

Meas. m/z	#	Ion Formula	m/z	err [ppm]	mSigma	# Sigma	Score	rdb	e⁻ Conf	N-Rule
430.1338	1	C24H18BN3NaO3	430.1338	-0.1	2.7	1	100.00	17.5	even	ok

Figure S18. HRMS (ESI$^+$) report of **4b**

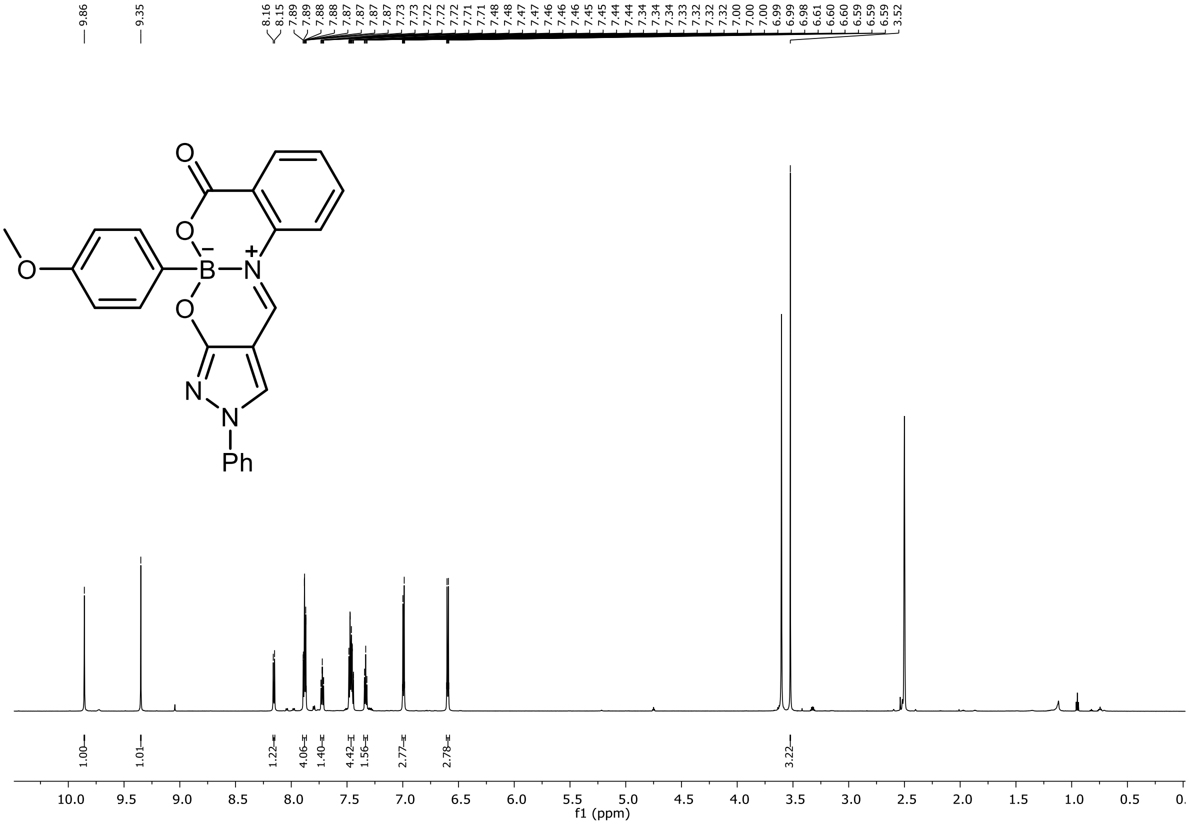

Figure S19. ^{1}H NMR (700 MHz, DMSO-d_6) spectrum of **4c**

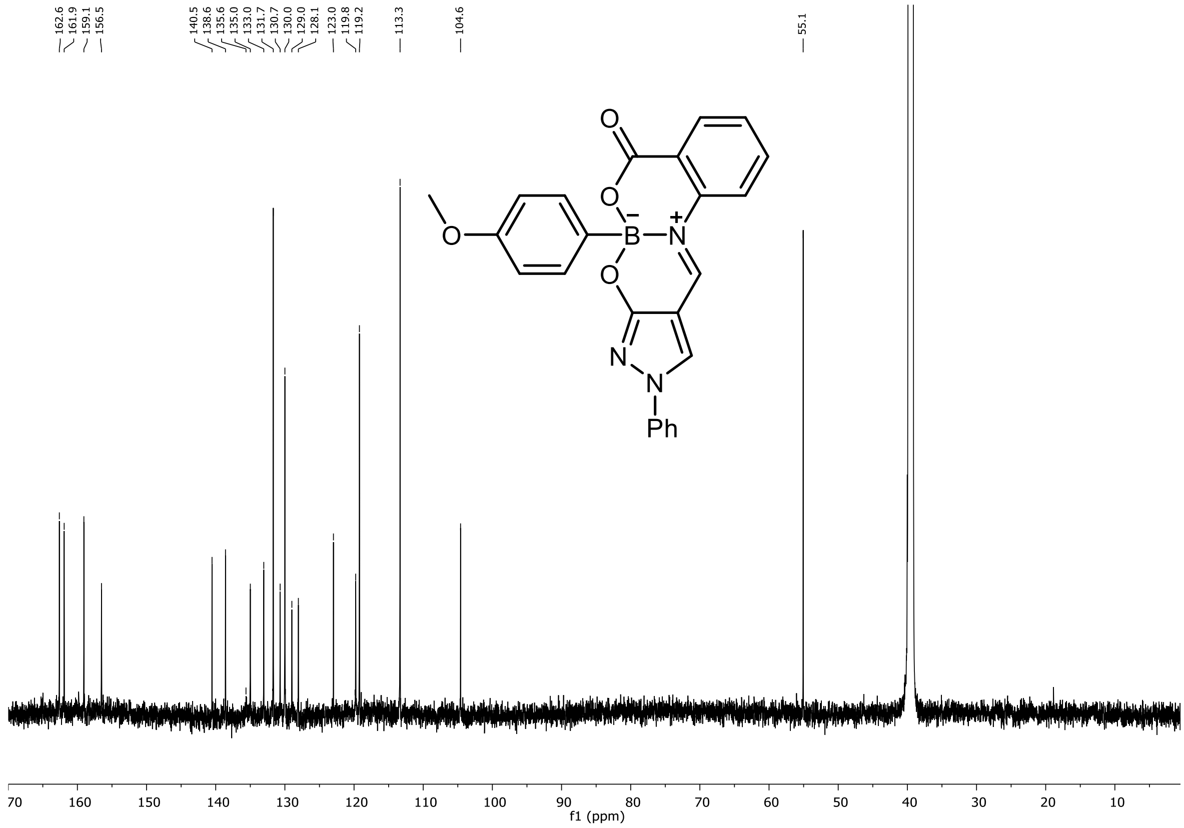

Figure S20. ^{13}C NMR (176 MHz, DMSO-d_6) spectrum of **4c**

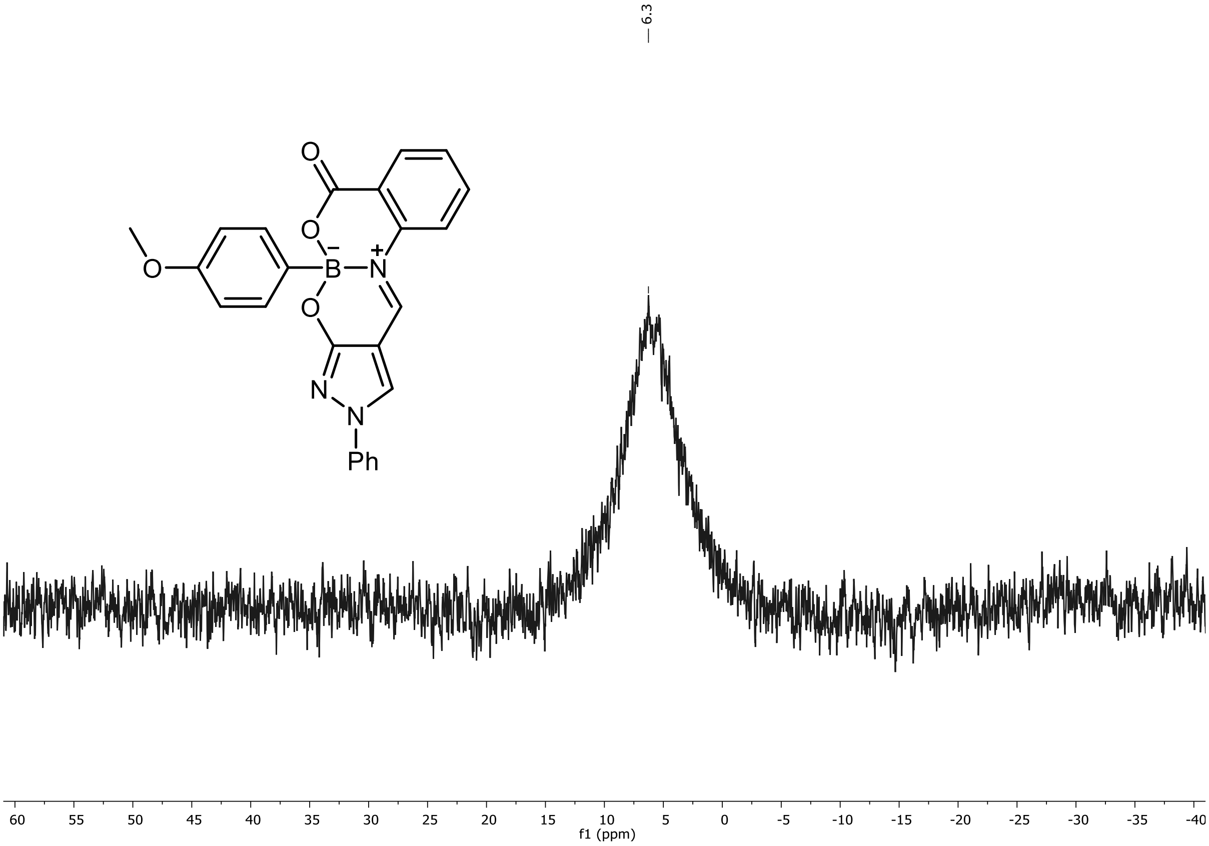

Figure S21. ^{11}B NMR (128 MHz, DMSO-d_6) spectrum of **4c**

Compound Spectrum SmartFormula Report

Analysis Info

Analysis Name	D:\Data\VDD-227.d	Acquisition Date	3/1/2024 6:55:13 PM
Method	DirectInfusion_TuneLow_pos.m	Operator	hplc
Sample Name	VDD-227	Instrument	micrOTOF-Q III 8228888.20448
Comment	AB		

Acquisition Parameter

Source Type	ESI	Ion Polarity	Positive	Set Nebulizer	0.4 Bar
Focus	Not active	Set Capillary	4500 V	Set Dry Heater	180 °C
Scan Begin	50 m/z	Set End Plate Offset	-500 V	Set Dry Gas	4.0 l/min
Scan End	1000 m/z	Set Collision Cell RF	140.0 Vpp	Set Divert Valve	Waste

#	RT [min]	Area	Int. Type	I	S/N	Chromatogram	Max. m/z	FWHM [min]
n.a.	6.8	n.a.	Single spectrum	n.a.	n.a.	n.a.	446.1292	n.a.

+MS, 6.8min #410

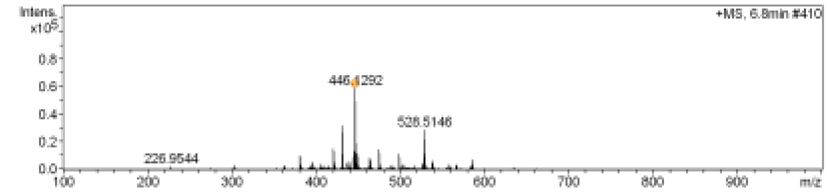

Meas. m/z	#	Ion Formula	m/z	err [ppm]	mSigma	# Sigma	Score	rdb	e⁻ Conf	N-Rule
446.1292	1	C24H18BN3NaO4	446.1287	1.3	11.7	4	100.00	17.5	even	ok

Figure S22. HRMS (ESI$^+$) report of **4c**

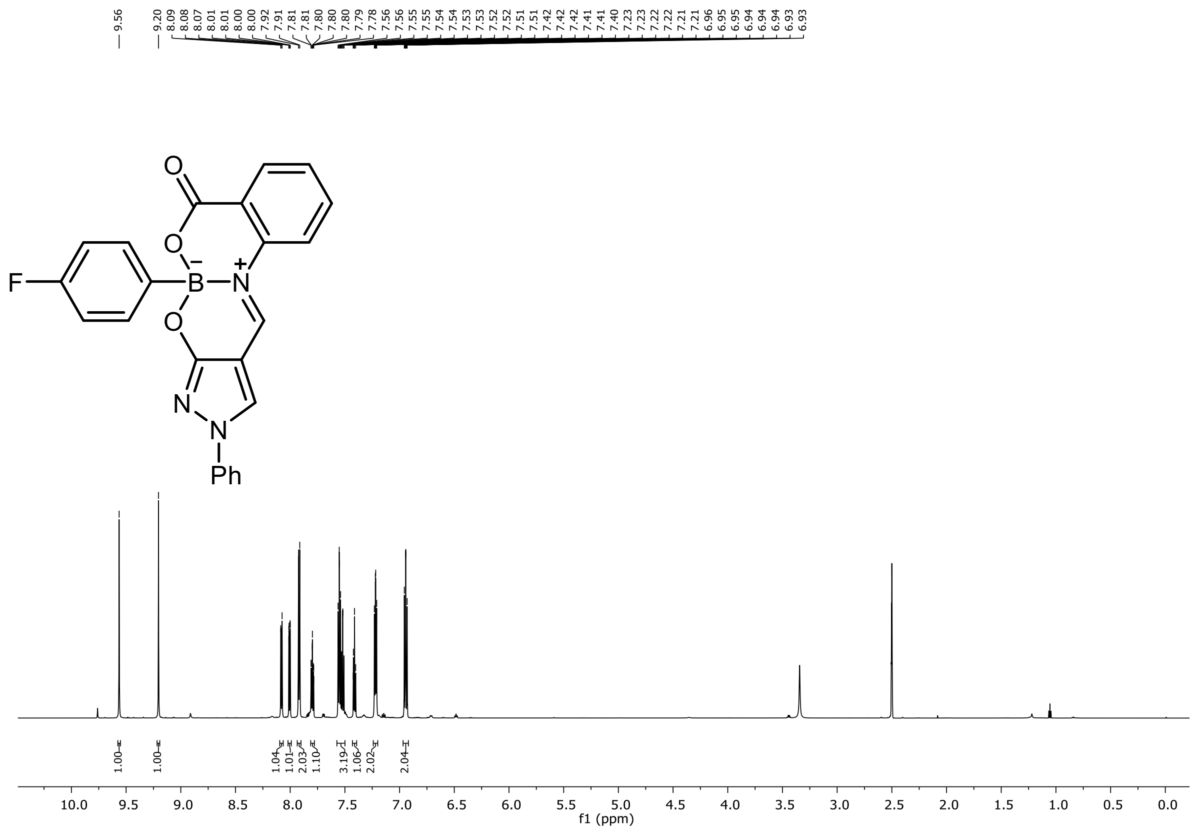

Figure S23. ^{1}H NMR (700 MHz, DMSO-d_6) spectrum of **4d**

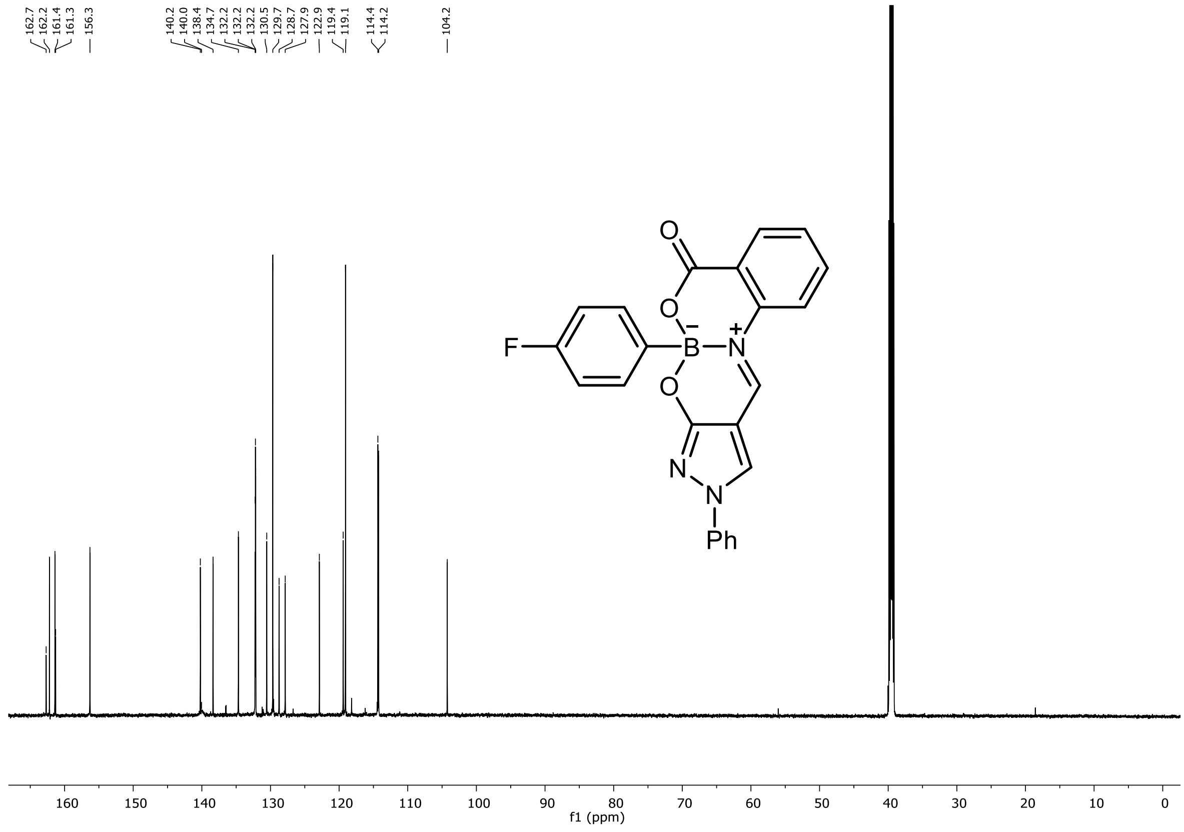

Figure S24. ^{13}C NMR (176 MHz, DMSO-d_6) spectrum of **4d**

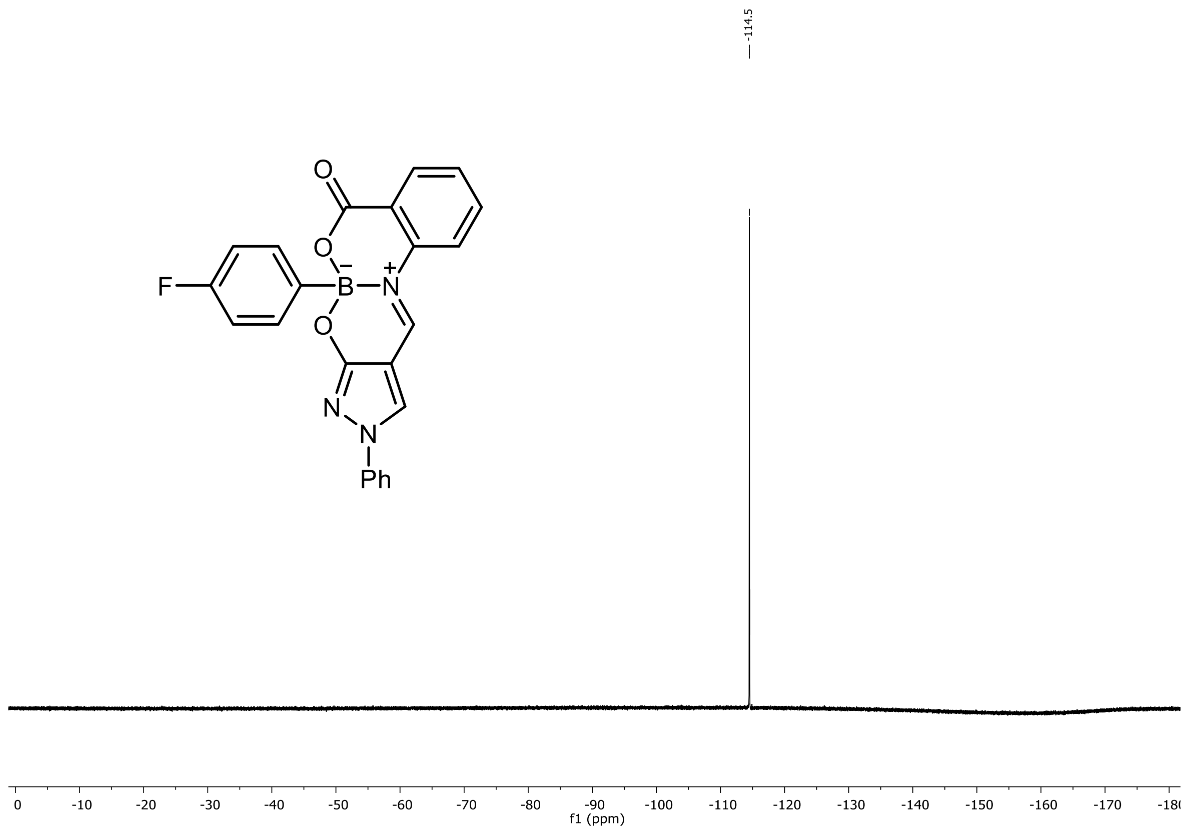

Figure S25. ^{19}F NMR (376 MHz, DMSO-d_6) spectrum of **4d**

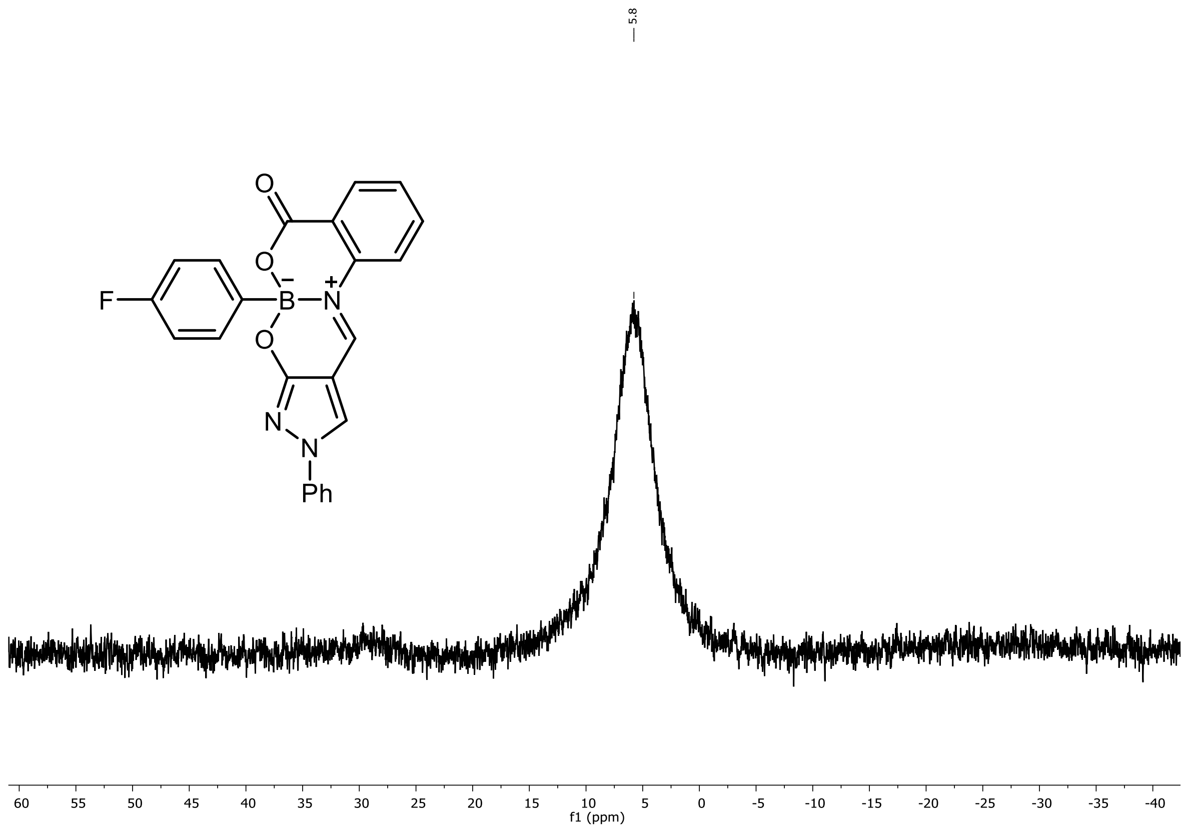

Figure S26. ^{11}B NMR (128 MHz, DMSO-d_6) spectrum of **4d**

Compound Spectrum SmartFormula Report

Analysis Info

Analysis Name	D:\Data\VDD-230.d
Method	DirectInfusion_TuneLow_pos.m
Sample Name	VDD-230
Comment	AB
Acquisition Date	3/3/2024 6:34:47 PM
Operator	hplc
Instrument	micrOTOF-Q III 8228888.20448

Acquisition Parameter

Source Type	ESI	Ion Polarity	Positive	Set Nebulizer	0.4 Bar
Focus	Not active	Set Capillary	4500 V	Set Dry Heater	180 °C
Scan Begin	50 m/z	Set End Plate Offset	-500 V	Set Dry Gas	4.0 l/min
Scan End	1000 m/z	Set Collision Cell RF	140.0 Vpp	Set Divert Valve	Waste

#	RT [min]	Area	Int. Type	I	S/N	Chromatogram	Max. m/z	FWHM [min]
n.a.	3.6	n.a.	Single spectrum	n.a.	n.a.	n.a.	434.1085	n.a.

+MS, 3.6min #216

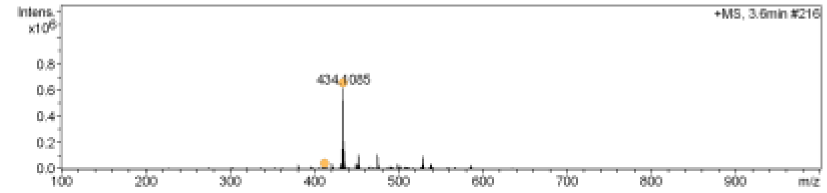

Meas. m/z	#	Ion Formula	m/z	err [ppm]	mSigma	# Sigma	Score	rdb	e⁻ Conf	N-Rule
412.1257	1	C23H16BFN3O3	412.1267	2.6	84.1	1	100.00	17.5	even	ok
434.1085	1	C23H15BFN3NaO3	434.1087	0.5	8.0	2	100.00	17.5	even	ok

Figure S27. HRMS (ESI$^+$) report of **4d**

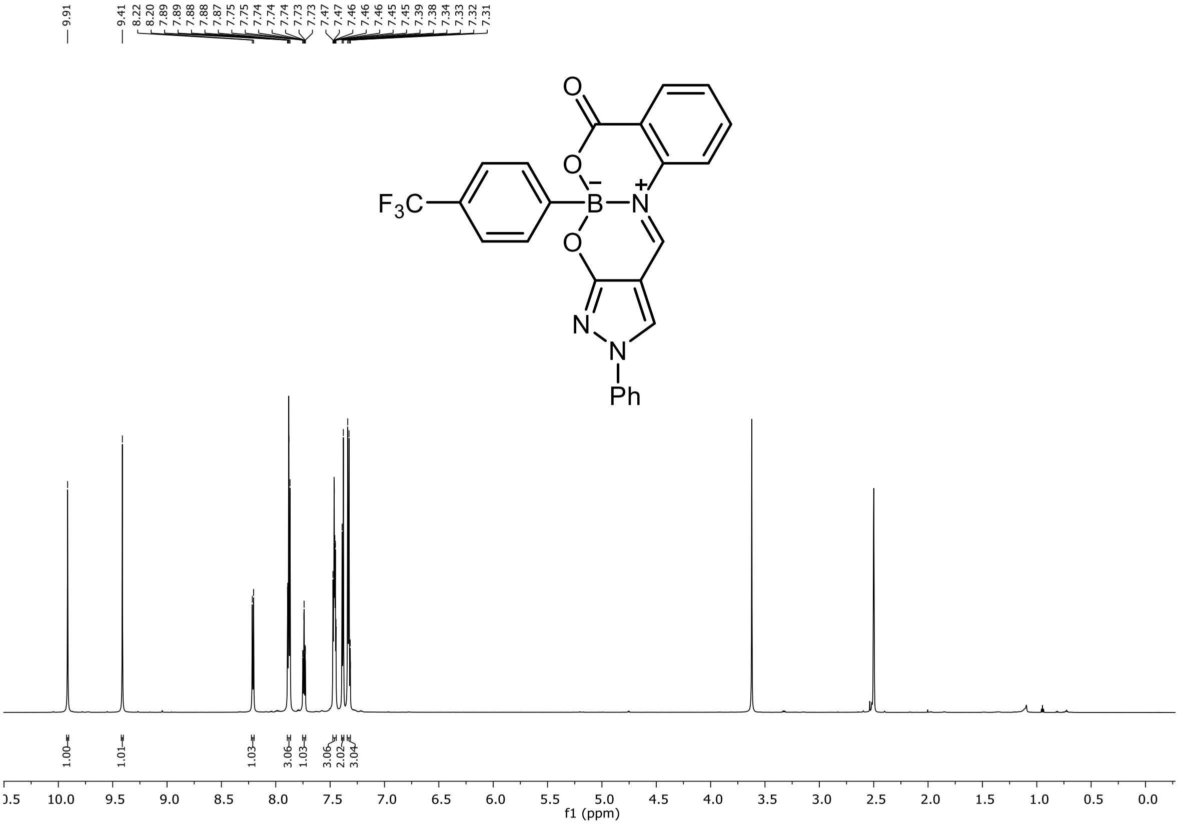

Figure S28. ^{1}H NMR (700 MHz, DMSO-d_6) spectrum of **4e**

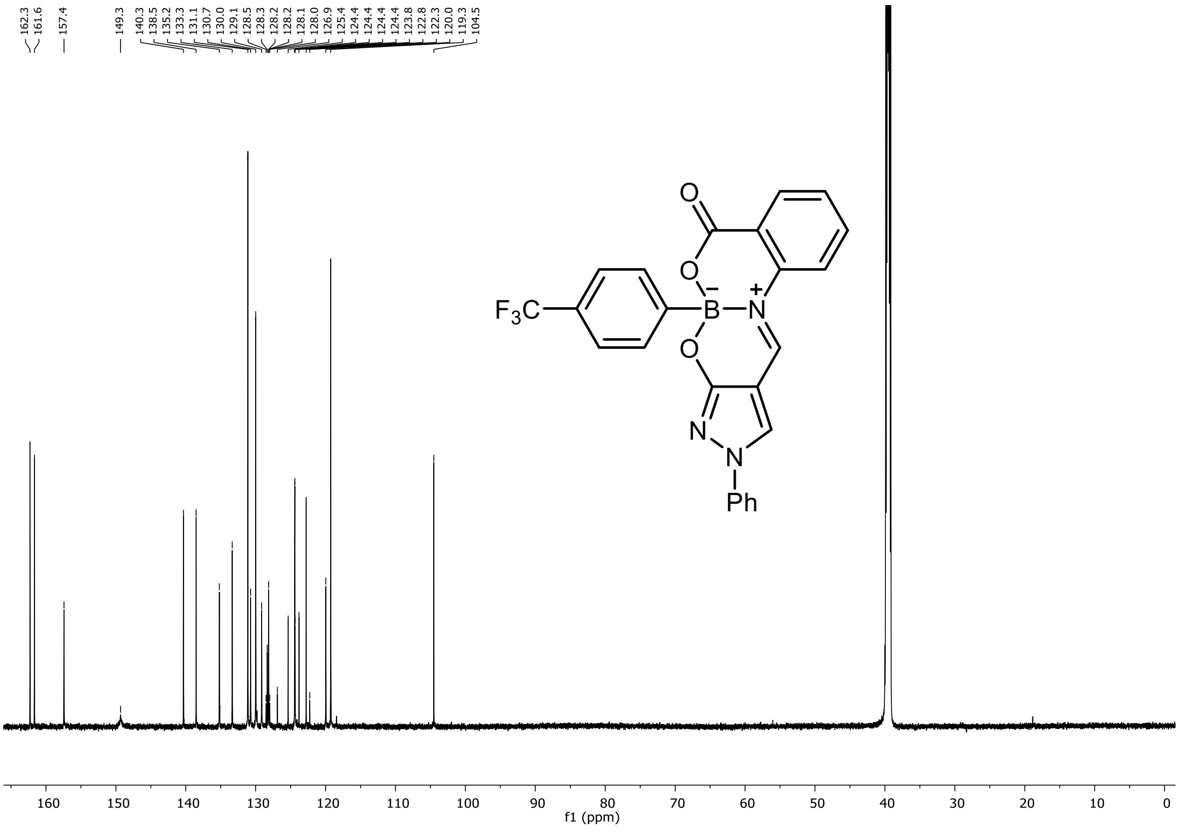

Figure S29. ^{13}C NMR (176 MHz, DMSO-d_6) spectrum of **4e**

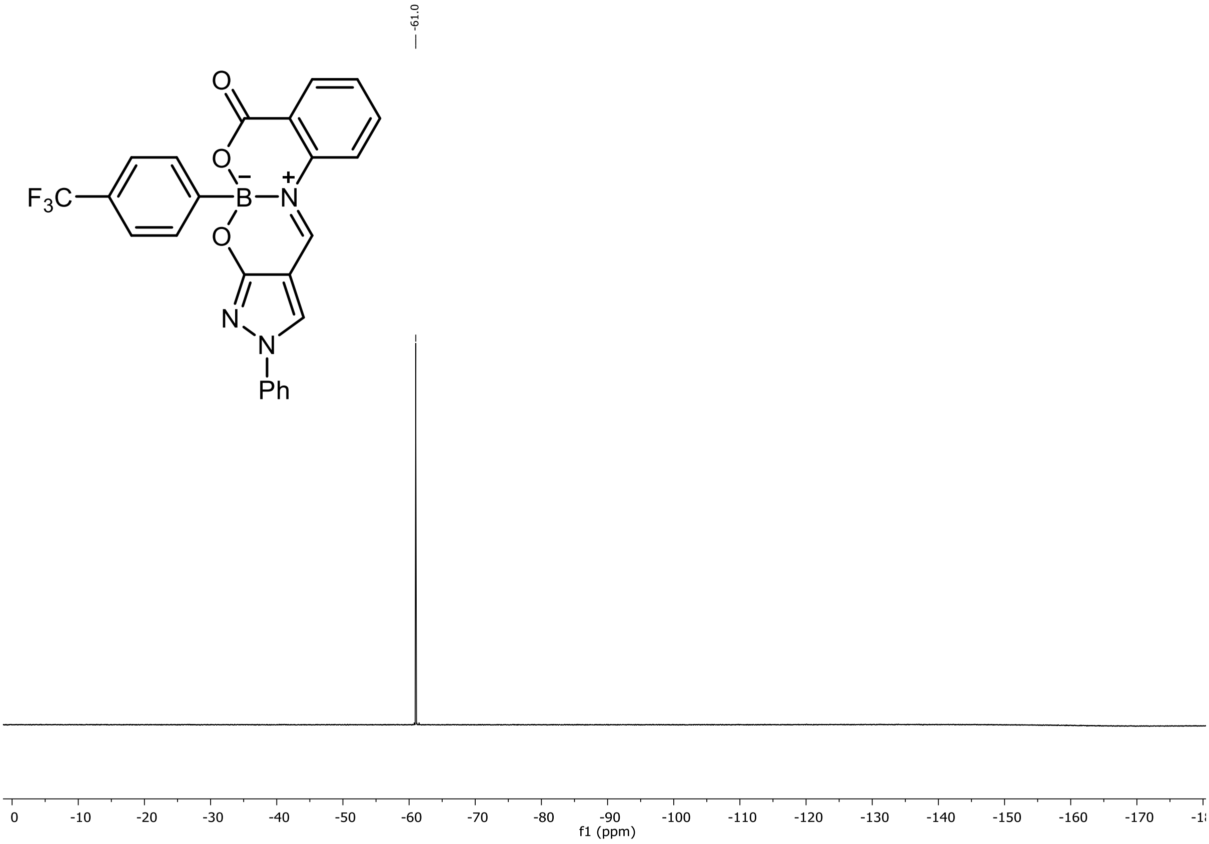

Figure S30. ^{19}F NMR (376 MHz, DMSO-d_6) spectrum of **4e**

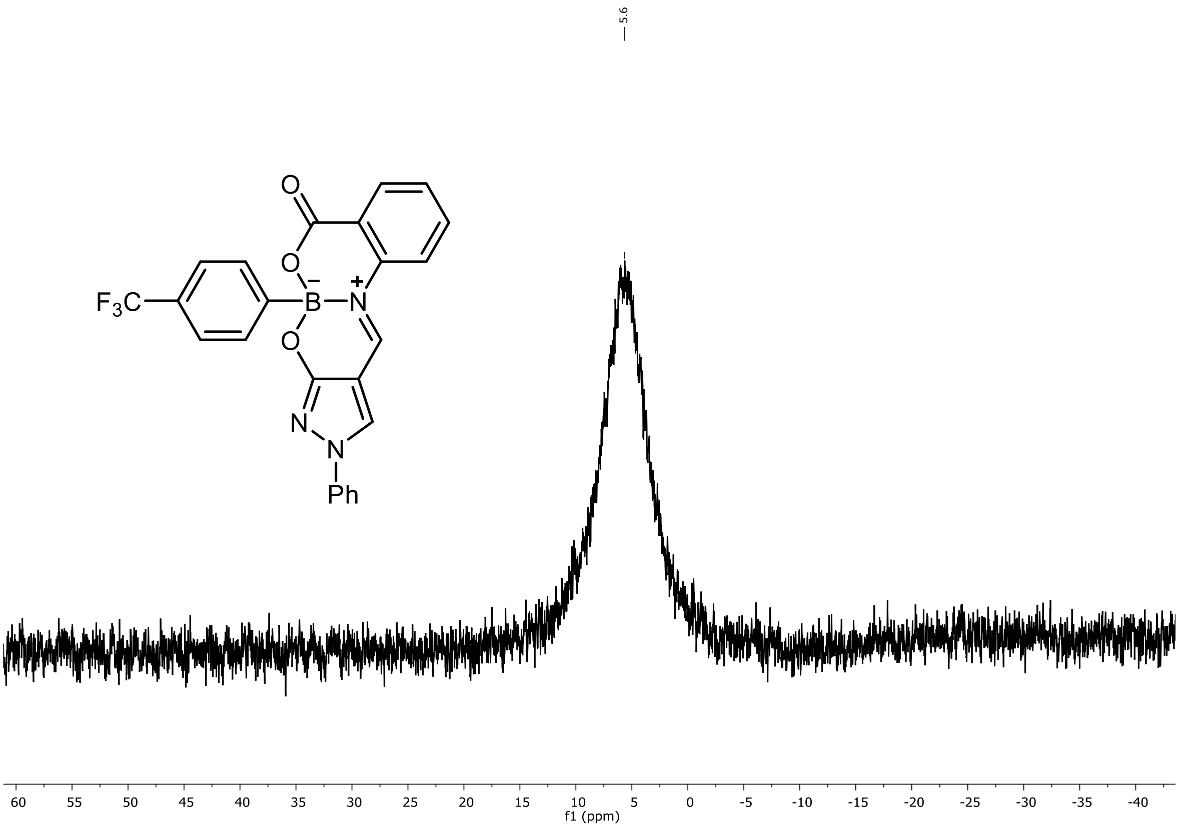

Figure S31. ^{11}B NMR (128 MHz, DMSO-d_6) spectrum of **4e**

Compound Spectrum SmartFormula Report

Analysis Info

Analysis Name	D:\Data\VDD-231.d	Acquisition Date	3/3/2024 6:47:20 PM
Method	DirectInfusion_TuneLow_pos.m	Operator	hplc
Sample Name	VDD-231	Instrument	micrOTOF-Q III 8228888.20448
Comment	AB		

Acquisition Parameter

Source Type	ESI	Ion Polarity	Positive	Set Nebulizer	0.4 Bar
Focus	Not active	Set Capillary	4500 V	Set Dry Heater	180 °C
Scan Begin	50 m/z	Set End Plate Offset	-500 V	Set Dry Gas	4.0 l/min
Scan End	1000 m/z	Set Collision Cell RF	140.0 Vpp	Set Divert Valve	Waste

#	RT [min]	Area	Int. Type	I	S/N	Chromatogram	Max. m/z	FWHM [min]
n.a.	4.0	n.a.	Single spectrum	n.a.	n.a.	n.a.	484.1059	n.a.

+MS, 4.0min #241

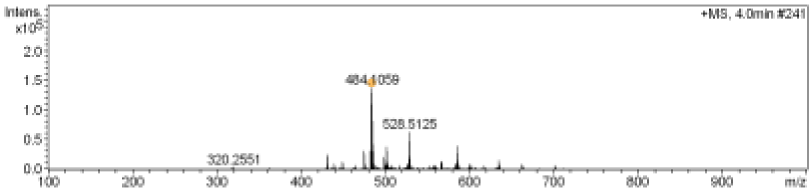

Meas. m/z	#	Ion Formula	m/z	err [ppm]	mSigma	# Sigma	Score	rdb	e⁻ Conf	N-Rule
484.1059	1	C24H15BF3N3NaO3	484.1055	-0.7	5.7	3	100.00	17.5	even	ok

Figure S32. HRMS (ESI$^+$) report of **4e**

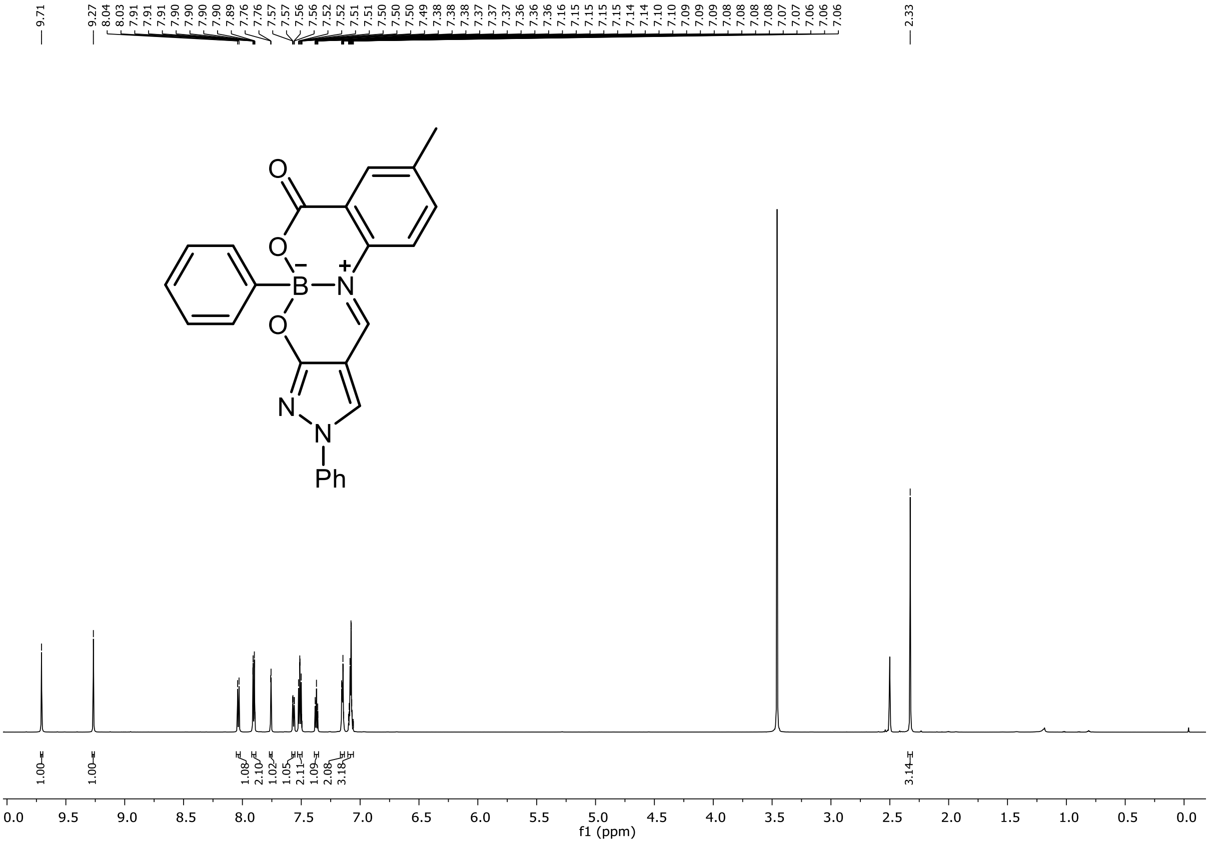

Figure S33. ^{1}H NMR (700 MHz, DMSO-d_6) spectrum of **4f**

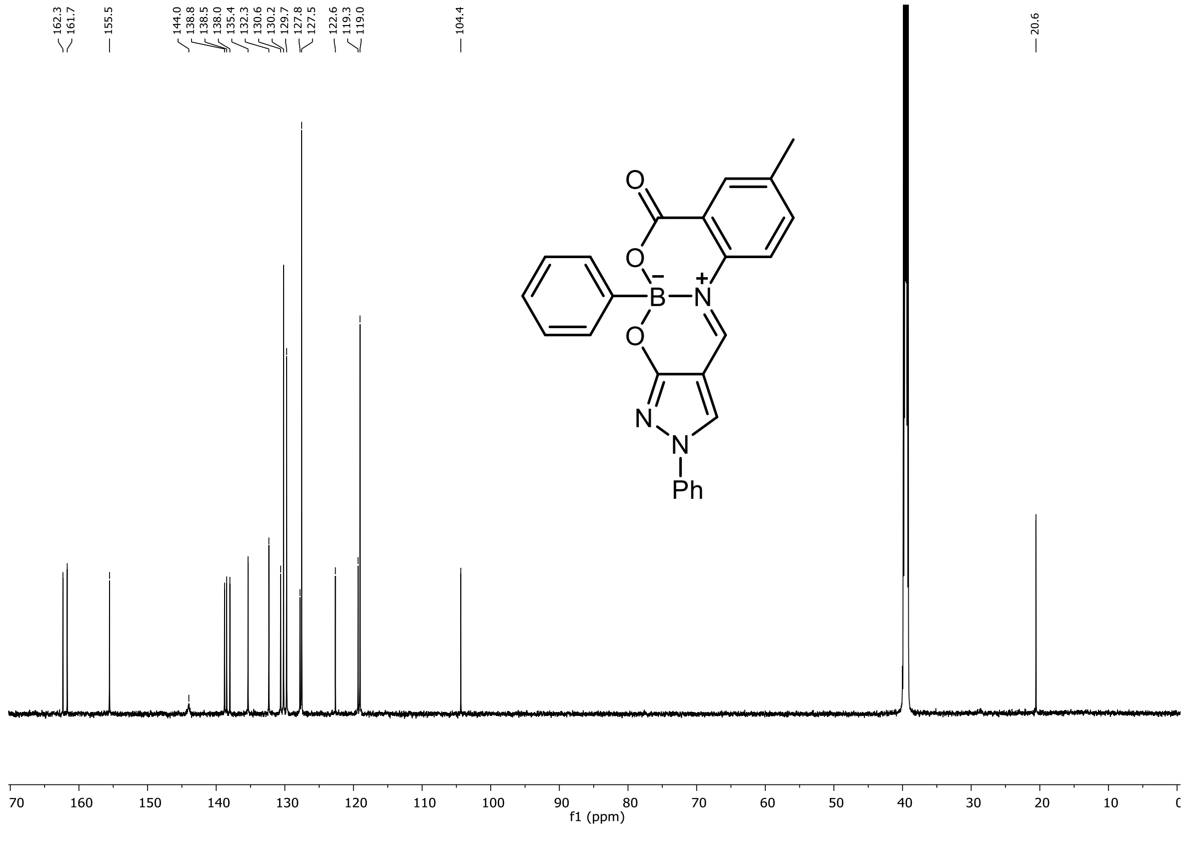

Figure S34. ^{13}C NMR (176 MHz, DMSO-d_6) spectrum of **4f**

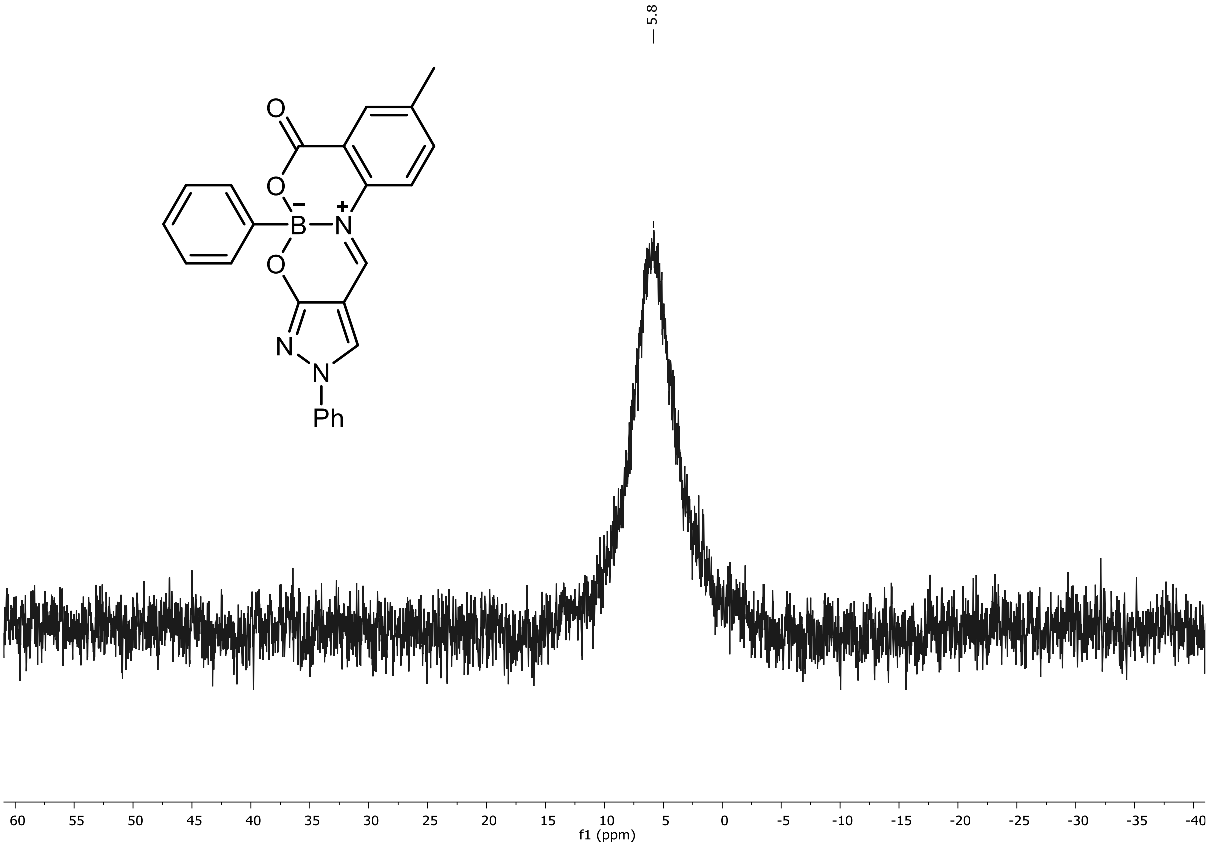

Figure S35. ^{11}B NMR (128 MHz, DMSO-d_6) spectrum of **4f**

Compound Spectrum SmartFormula Report

Analysis Info

Analysis Name	D:\Data\VDD-242.d	Acquisition Date	3/1/2024 8:11:21 PM
Method	DirectInfusion_TuneLow_pos.m	Operator	hplc
Sample Name	VDD-242	Instrument	micrOTOF-Q III 8228888.20448
Comment	AB		

Acquisition Parameter

Source Type	ESI	Ion Polarity	Positive	Set Nebulizer	0.4 Bar
Focus	Not active	Set Capillary	4500 V	Set Dry Heater	180 °C
Scan Begin	50 m/z	Set End Plate Offset	-500 V	Set Dry Gas	4.0 l/min
Scan End	1000 m/z	Set Collision Cell RF	140.0 Vpp	Set Divert Valve	Waste

#	RT [min]	Area	Int. Type	I	S/N	Chromatogram	Max. m/z	FWHM [min]
n.a.	8.1	n.a.	Single spectrum	n.a.	n.a.	n.a.	430.1340	n.a.

+MS, 8.1min #486

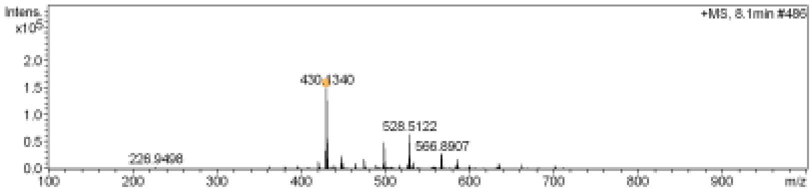

Meas. m/z	#	Ion Formula	m/z	err [ppm]	mSigma	# Sigma	Score	rdb	e⁻ Conf	N-Rule
430.1340	1	C24H18BN3NaO3	430.1338	0.4	3.6	1	100.00	17.5	even	ok

Figure S36. HRMS (ESI$^+$) report of **4f**

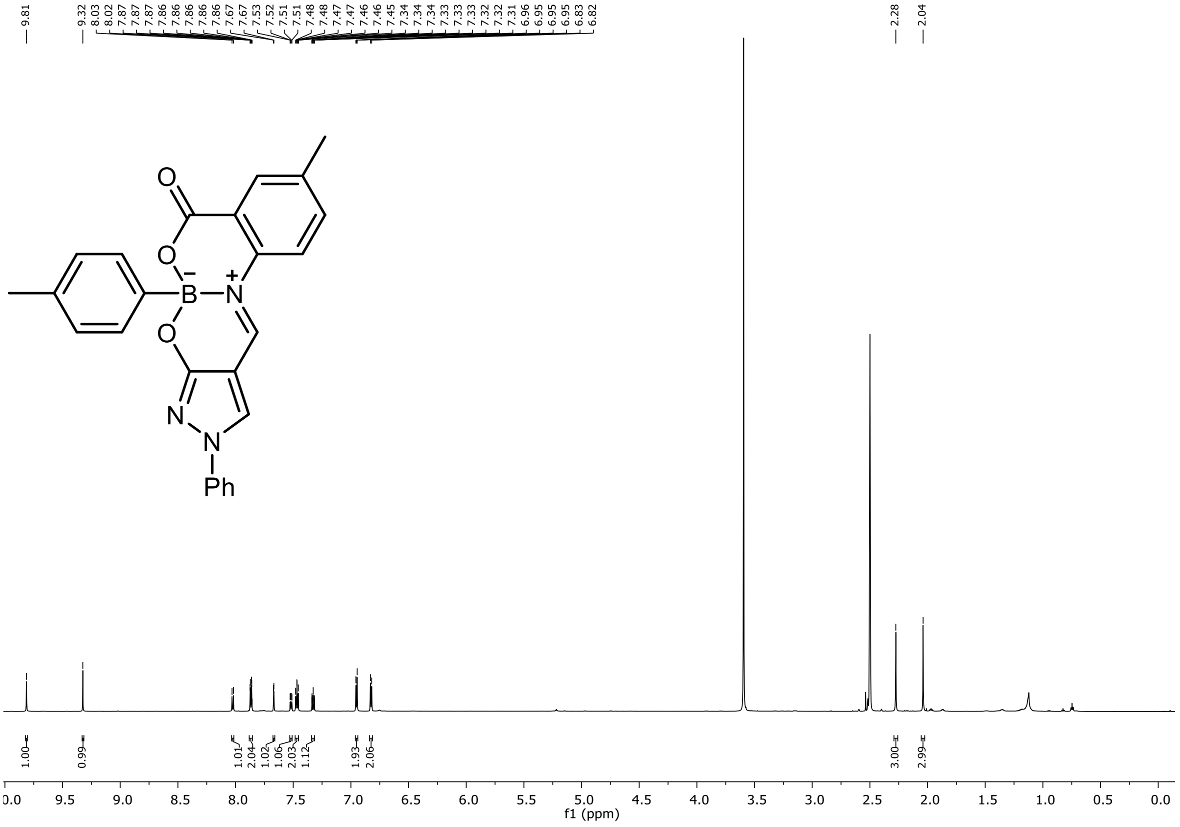

Figure S37. ^{1}H NMR (700 MHz, DMSO-d_6) spectrum of **4g**

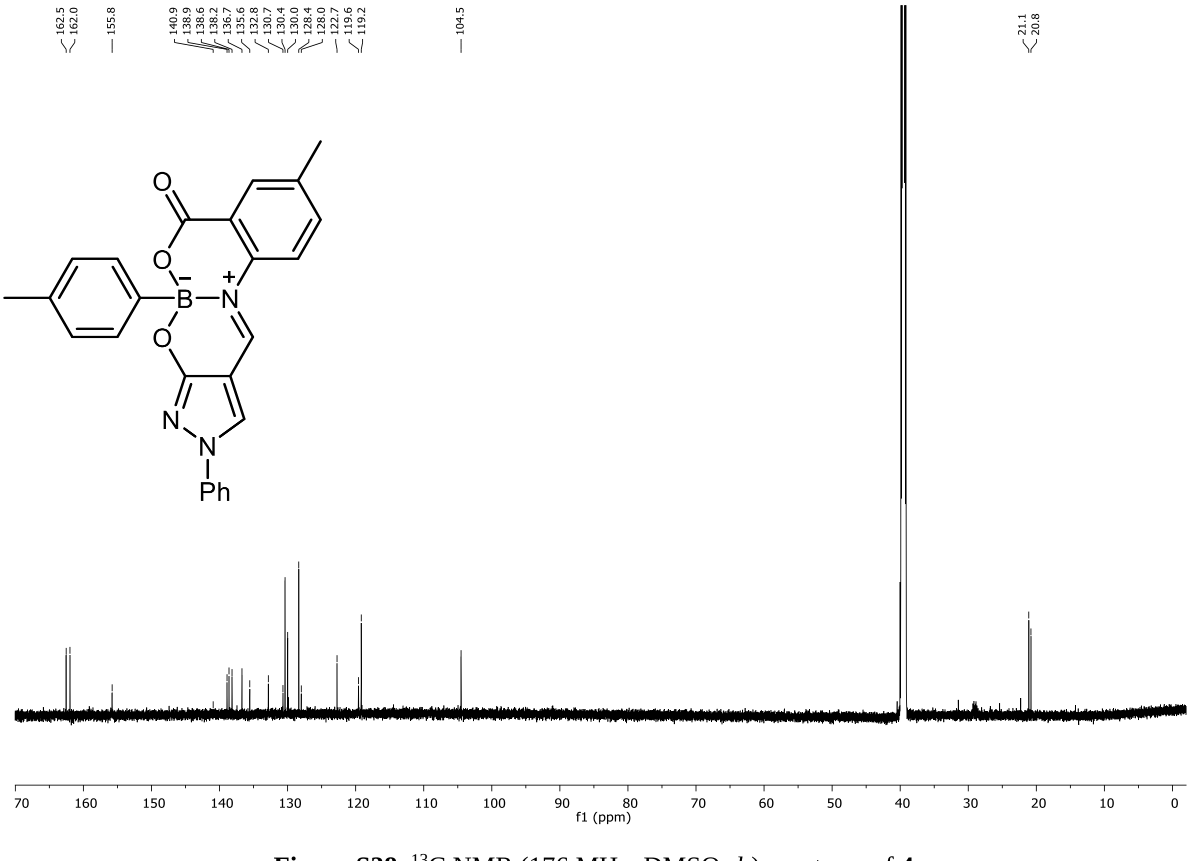

Figure S38. ^{13}C NMR (176 MHz, DMSO-d_6) spectrum of **4g**

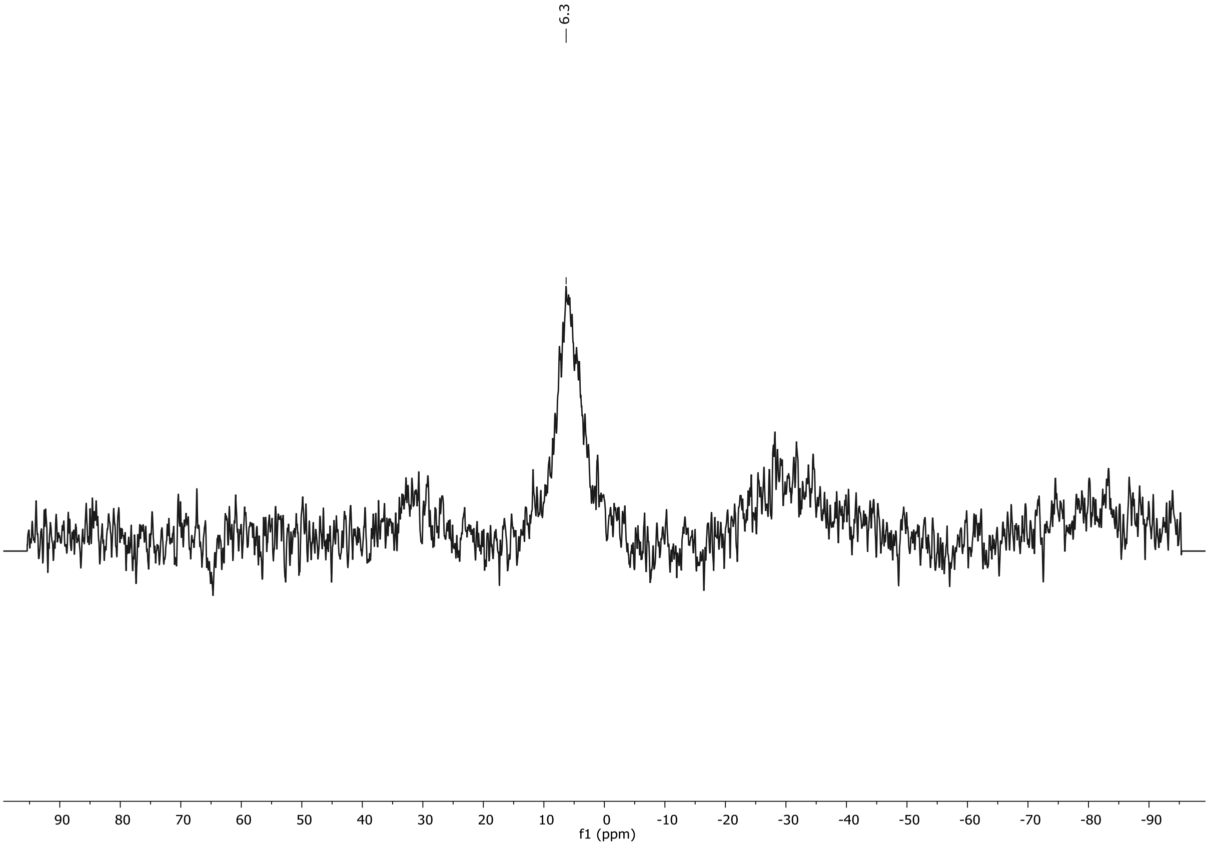

Figure S39. ^{11}B NMR (128 MHz, DMSO-d_6) spectrum of **4g**

Compound Spectrum SmartFormula Report

Analysis Info

Analysis Name	D:\Data\VDD-249.d	Acquisition Date	3/3/2024 7:52:58 PM
Method	DirectInfusion_TuneLow_pos.m	Operator	hplc
Sample Name	VDD-249	Instrument	micrOTOF-Q III 8228888.20448
Comment	AB		

Acquisition Parameter

Source Type	ESI	Ion Polarity	Positive	Set Nebulizer	0.4 Bar
Focus	Not active	Set Capillary	4500 V	Set Dry Heater	180 °C
Scan Begin	50 m/z	Set End Plate Offset	-500 V	Set Dry Gas	4.0 l/min
Scan End	1000 m/z	Set Collision Cell RF	140.0 Vpp	Set Divert Valve	Waste

#	RT [min]	Area	Int. Type	I	S/N	Chromatogram	Max. m/z	FWHM [min]
n.a.	3.4	n.a.	Single spectrum	n.a.	n.a.	n.a.	444.1495	n.a.

+MS, 3.4min #201

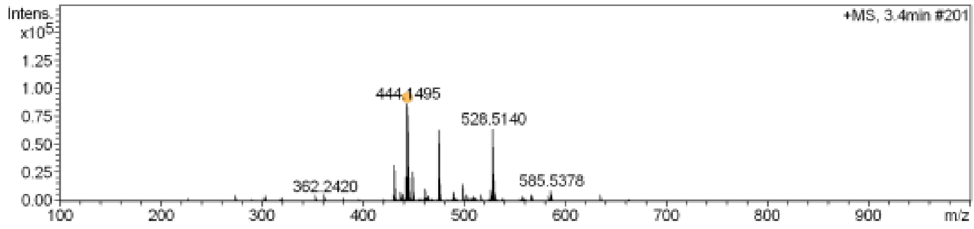

Meas. m/z	#	Ion Formula	m/z	err [ppm]	mSigma	# Sigma	Score	rdb	e⁻ Conf	N-Rule
444.1495	1	C25H20BN3NaO3	444.1494	-0.2	9.6	2	100.00	17.5	even	ok

Figure S40. HRMS (ESI$^+$) report of **4g**

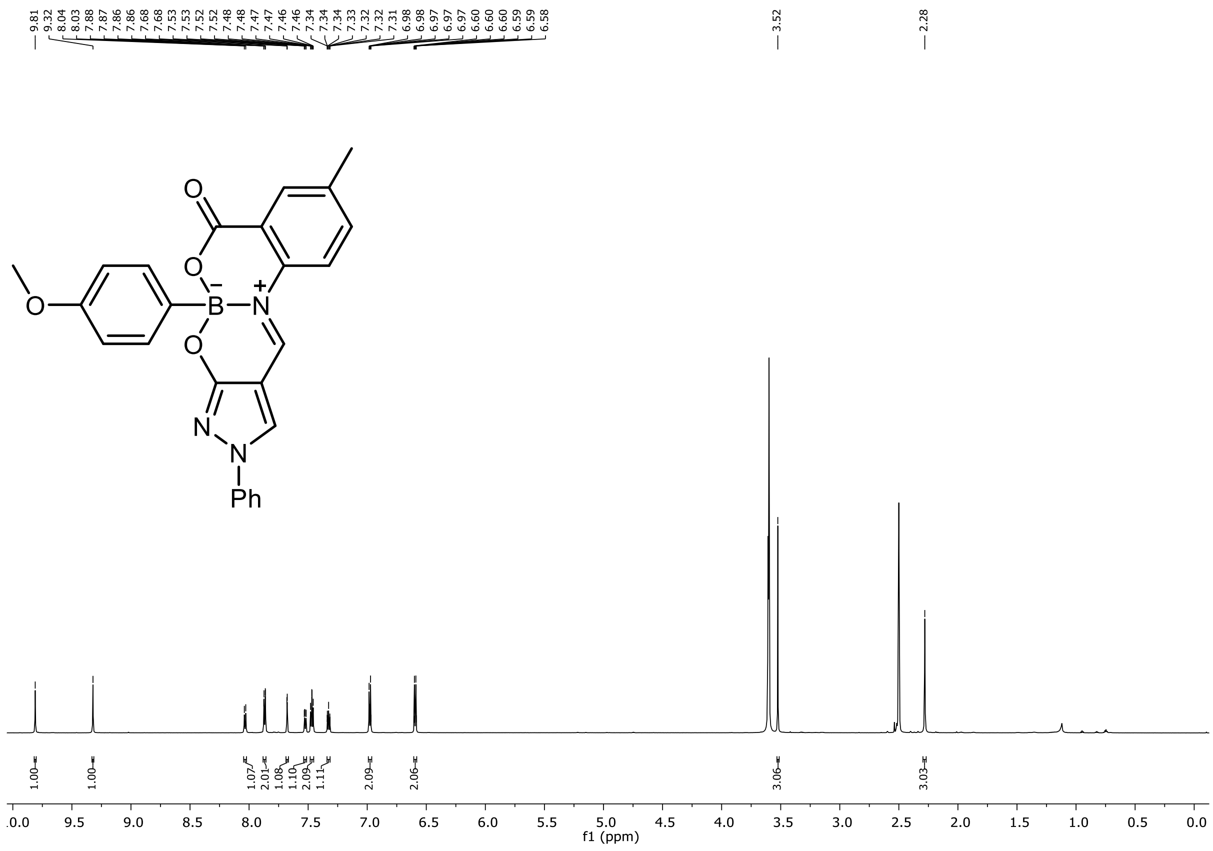

Figure S41. ^{1}H NMR (700 MHz, DMSO-d_6) spectrum of **4h**

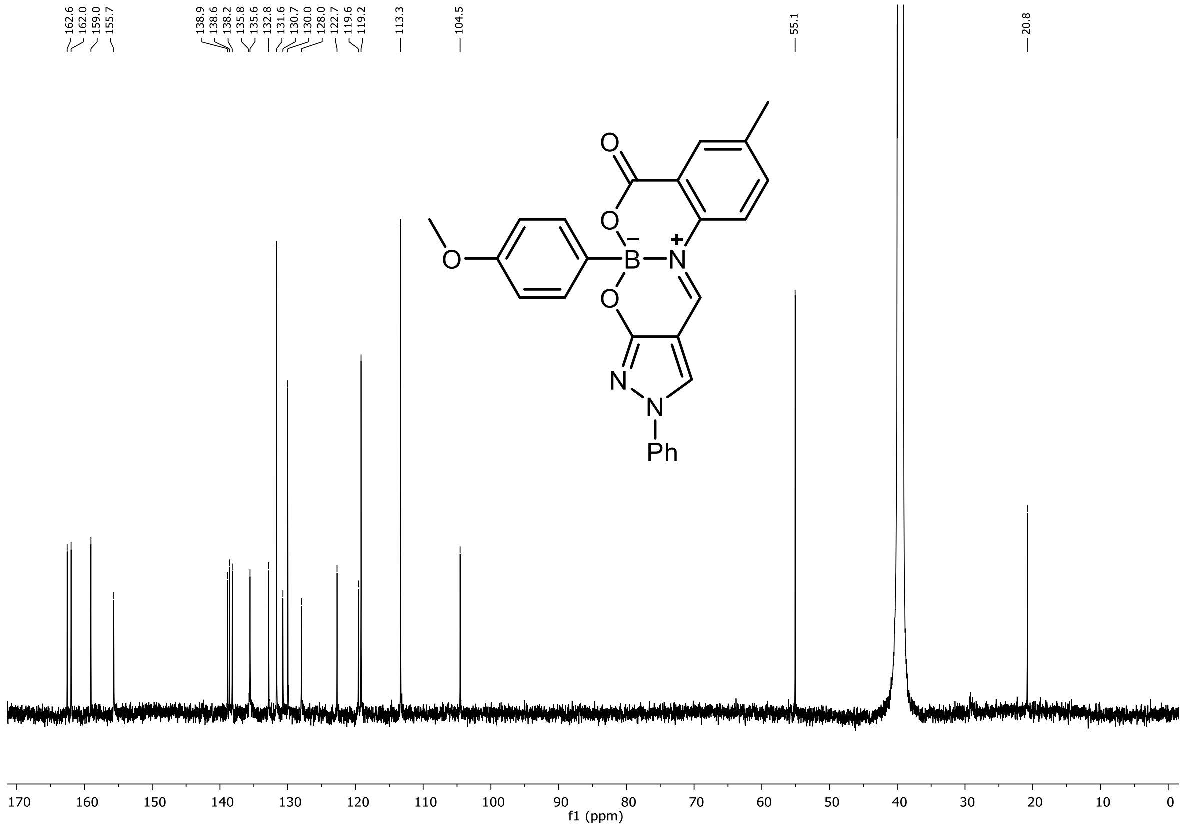

Figure S42. ^{13}C NMR (176 MHz, DMSO-d_6) spectrum of **4h**

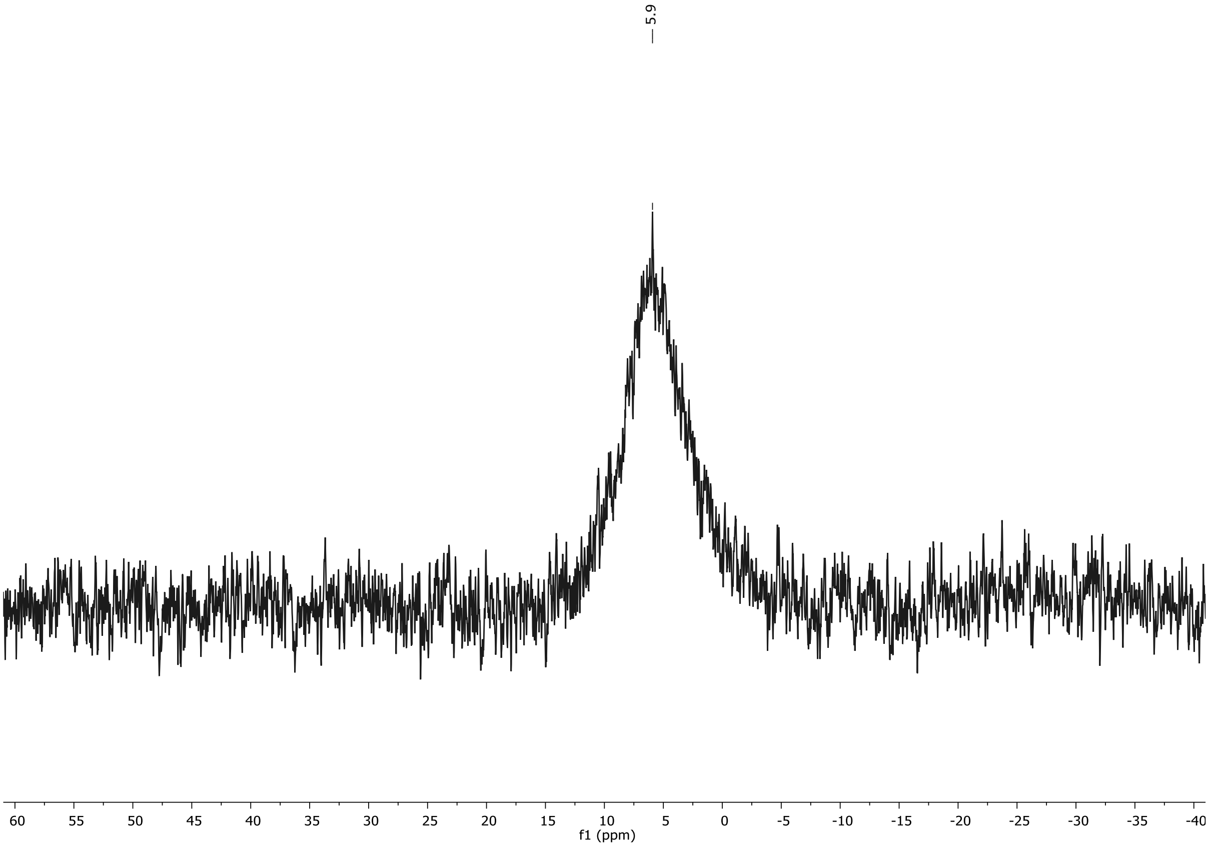

Figure S43. ^{11}B NMR (128 MHz, DMSO-d_6) spectrum of **4h**

Compound Spectrum SmartFormula Report

Analysis Info

Analysis Name	D:\Data\VDD-248.d	Acquisition Date	3/3/2024 7:36:01 PM
Method	DirectInfusion_TuneLow_pos.m	Operator	hplc
Sample Name	VDD-248	Instrument	micrOTOF-Q III 8228888.20448
Comment	AB		

Acquisition Parameter

Source Type	ESI	Ion Polarity	Positive	Set Nebulizer	0.4 Bar
Focus	Not active	Set Capillary	4500 V	Set Dry Heater	180 °C
Scan Begin	50 m/z	Set End Plate Offset	-500 V	Set Dry Gas	4.0 l/min
Scan End	1000 m/z	Set Collision Cell RF	140.0 Vpp	Set Divert Valve	Waste

#	RT [min]	Area	Int. Type	I	S/N	Chromatogram	Max. m/z	FWHM [min]
n.a.	3.1	n.a.	Single spectrum	n.a.	n.a.	n.a.	460.1447	n.a.

+MS, 3.1min #183

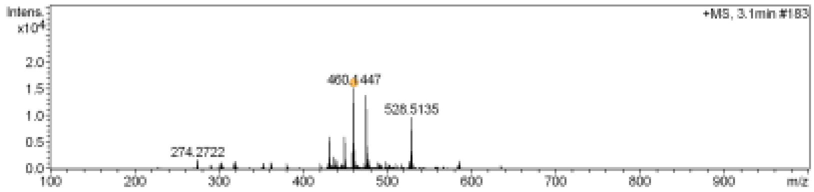

Meas. m/z	#	Ion Formula	m/z	err [ppm]	mSigma	# Sigma	Score	rdb	e⁻ Conf	N-Rule
460.1447	1	C25H20BN3NaO4	460.1444	-0.8	11.5	2	100.00	17.5	even	ok

Figure S44. HRMS (ESI$^+$) report of **4h**

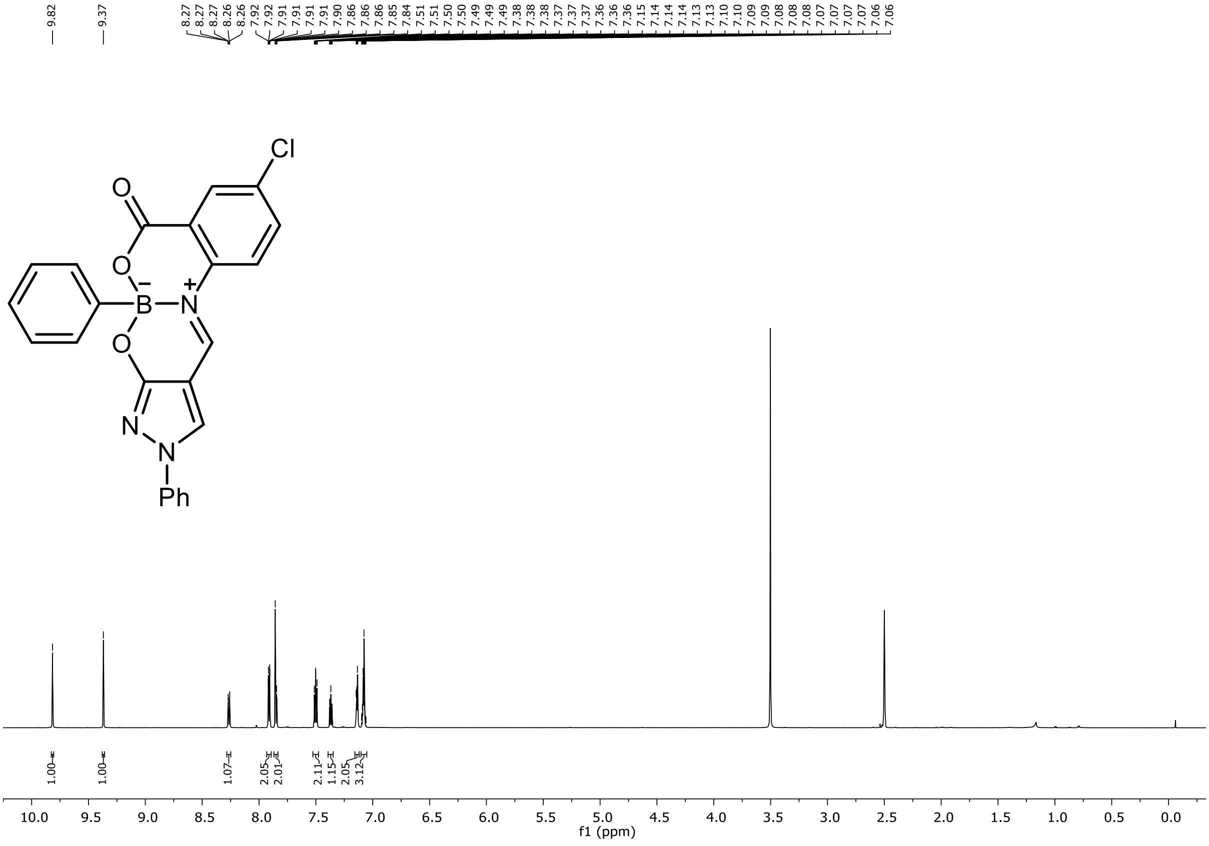

Figure S45. ^{1}H NMR (700 MHz, DMSO-d_6) spectrum of **4i**

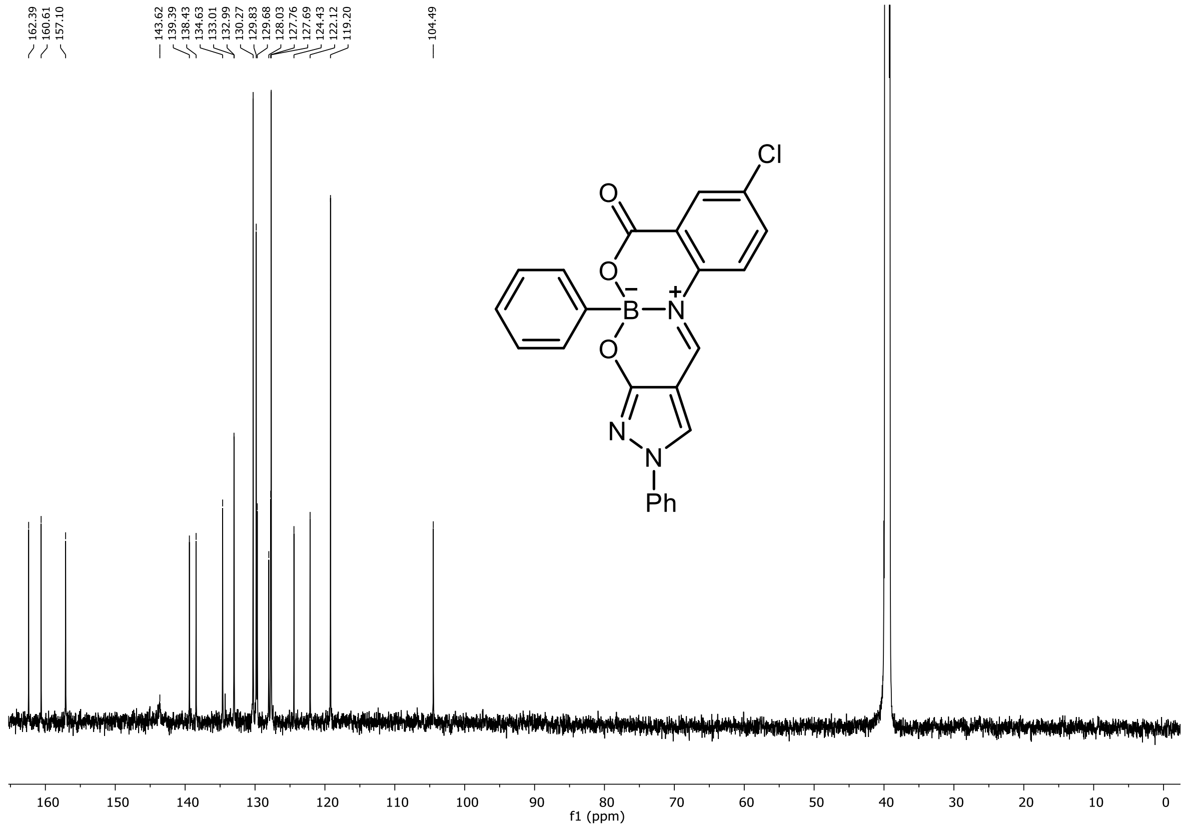

Figure S46. ^{13}C NMR (176 MHz, DMSO-d_6) spectrum of **4i**

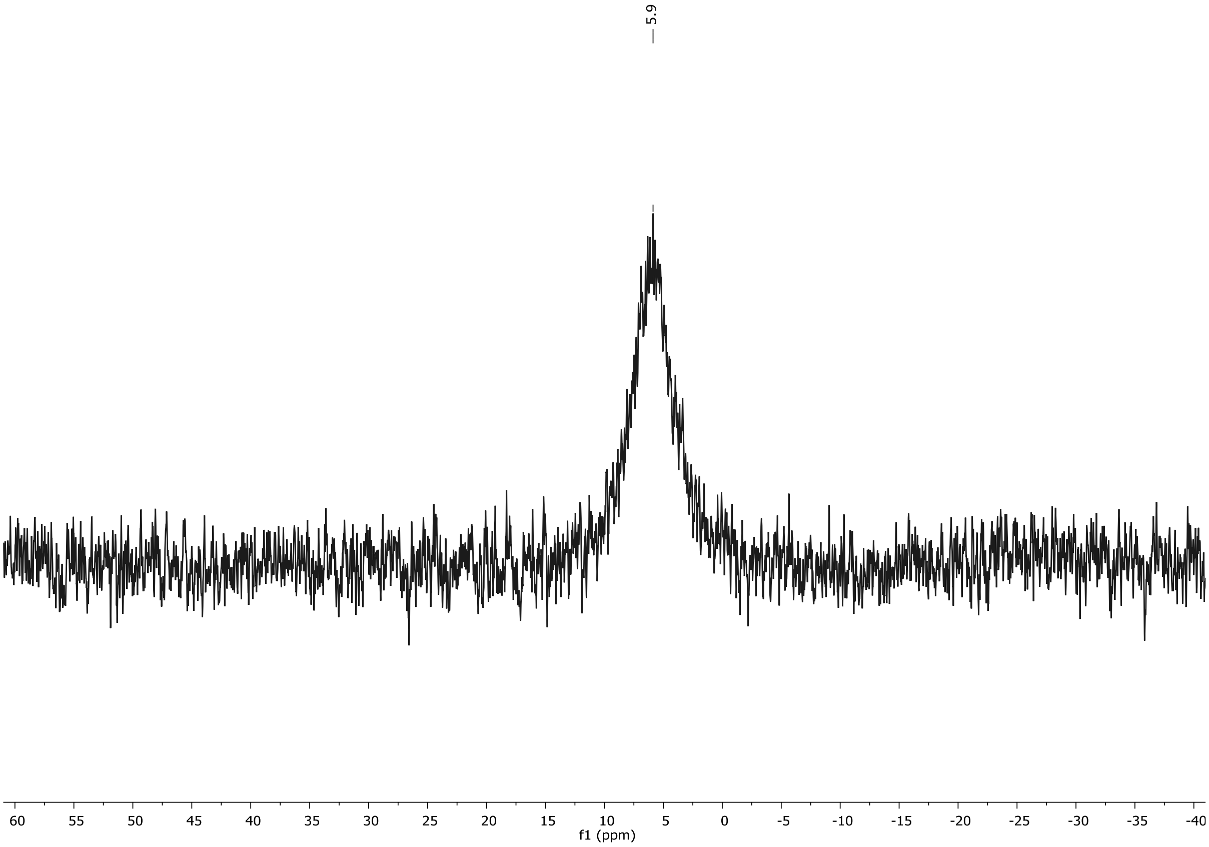

Figure S47. ^{11}B NMR (128 MHz, DMSO-d_6) spectrum of **4i**

Compound Spectrum SmartFormula Report

Analysis Info

Analysis Name	D:\Data\VDD-241.d	Acquisition Date	3/1/2024 7:56:46 PM
Method	DirectInfusion_TuneLow_pos.m	Operator	hplc
Sample Name	VDD-241	Instrument	micrOTOF-Q III 8228888.20448
Comment	AB		

Acquisition Parameter

Source Type	ESI	Ion Polarity	Positive	Set Nebulizer	0.4 Bar
Focus	Not active	Set Capillary	4500 V	Set Dry Heater	180 °C
Scan Begin	50 m/z	Set End Plate Offset	-500 V	Set Dry Gas	4.0 l/min
Scan End	1000 m/z	Set Collision Cell RF	140.0 Vpp	Set Divert Valve	Waste

#	RT [min]	Area	Int. Type	I	S/N	Chromatogram	Max. m/z	FWHM [min]
n.a.	4.4	n.a.	Single spectrum	n.a.	n.a.	n.a.	450.0794	n.a.

+MS, 4.4min #262

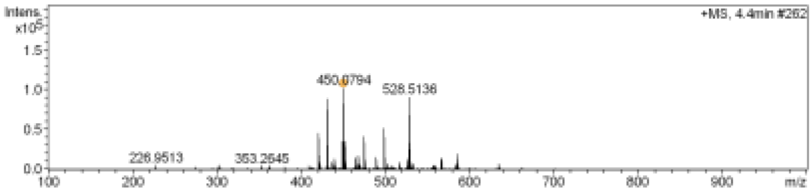

Meas. m/z	#	Ion Formula	m/z	err [ppm]	mSigma	# Sigma	Score	rdb	e⁻ Conf	N-Rule
450.0794	1	C23H15BClN3NaO3	450.0791	-0.6	6.5	1	100.00	17.5	even	ok

Figure S48. HRMS (ESI$^+$) report of **4i**

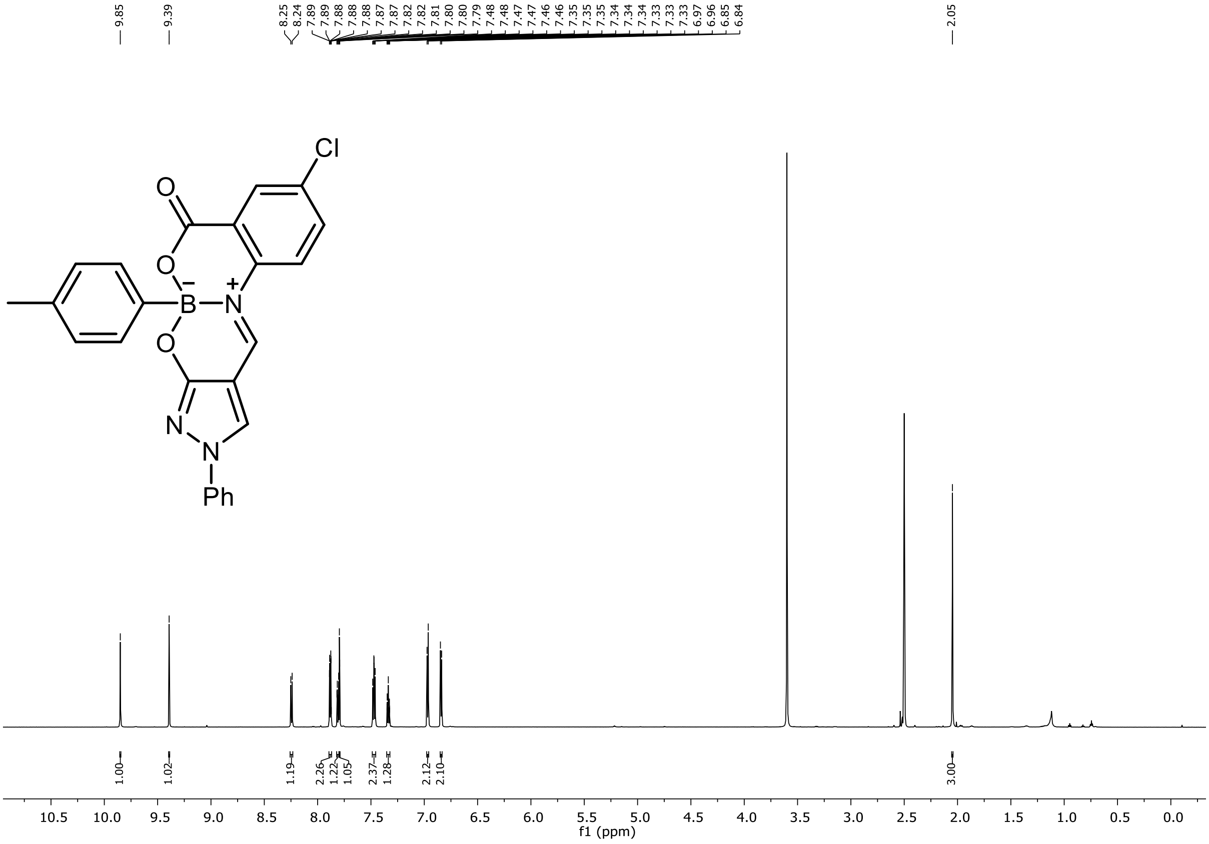

Figure S49. ^{1}H NMR (700 MHz, DMSO-d_6) spectrum of **4j**

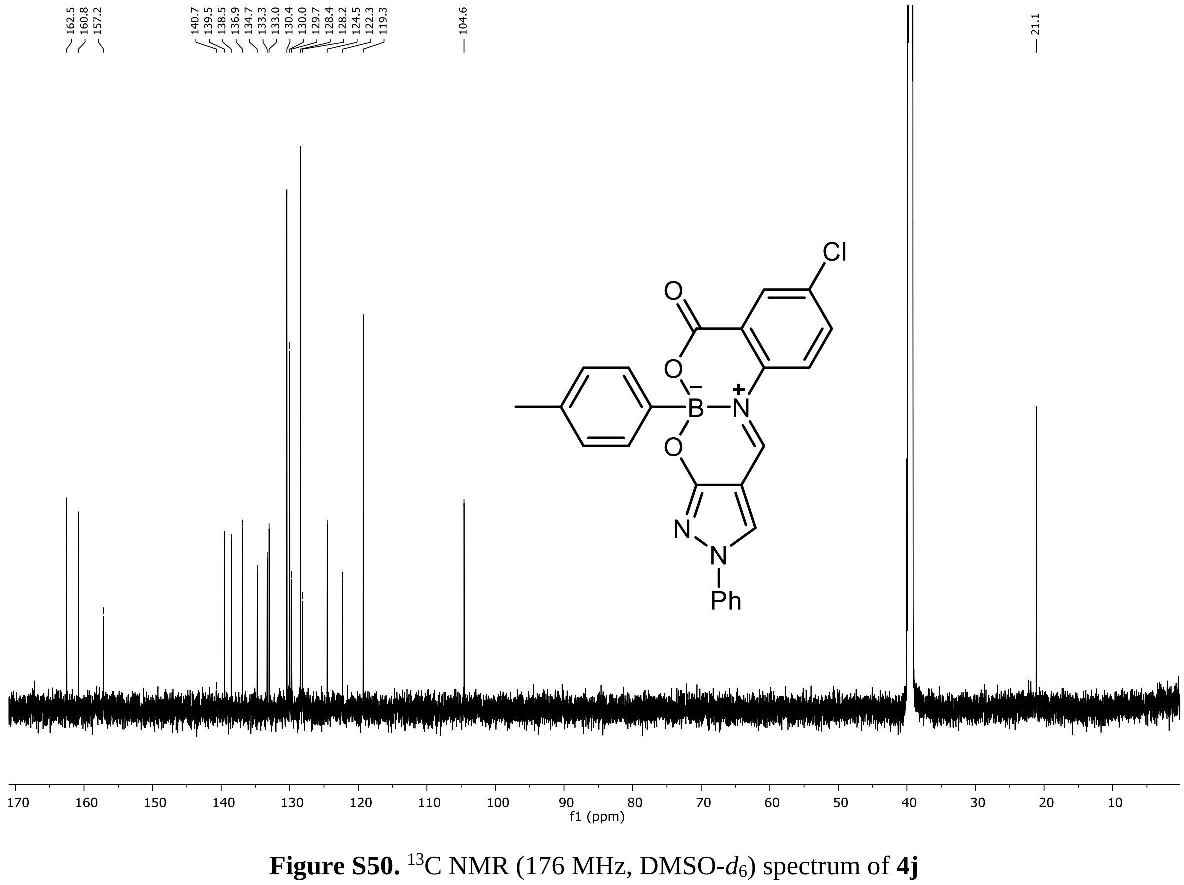

Figure S50. ^{13}C NMR (176 MHz, DMSO-d_6) spectrum of **4j**

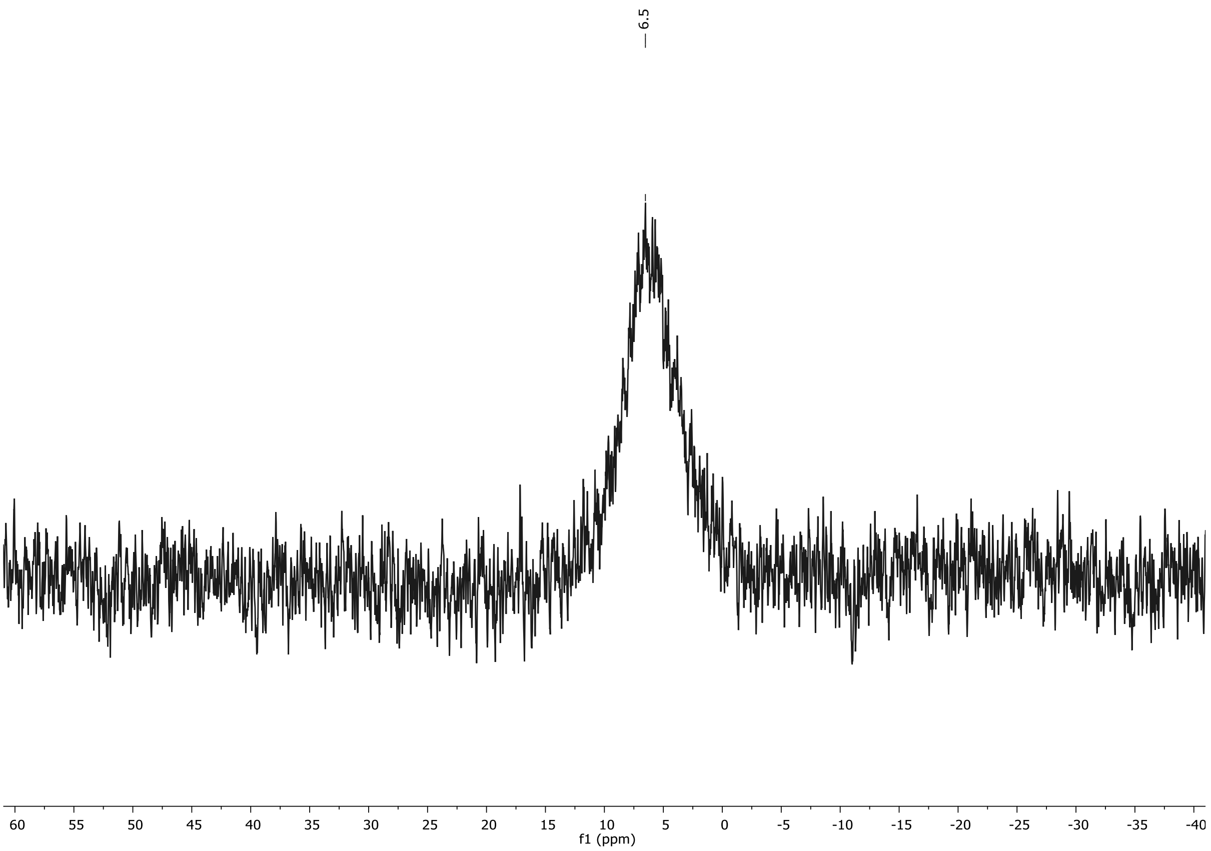

Figure S51. ^{11}B NMR (128 MHz, DMSO-d_6) spectrum of **4j**

Compound Spectrum SmartFormula Report

Analysis Info

Analysis Name	D:\Data\VDD-244.d	Acquisition Date	3/3/2024 7:00:35 PM
Method	DirectInfusion_TuneLow_pos.m	Operator	hplc
Sample Name	VDD-244	Instrument	micrOTOF-Q III 8228888.20448
Comment	AB		

Acquisition Parameter

Source Type	ESI	Ion Polarity	Positive	Set Nebulizer	0.4 Bar
Focus	Not active	Set Capillary	4500 V	Set Dry Heater	180 °C
Scan Begin	50 m/z	Set End Plate Offset	-500 V	Set Dry Gas	4.0 l/min
Scan End	1000 m/z	Set Collision Cell RF	140.0 Vpp	Set Divert Valve	Waste

#	RT [min]	Area	Int. Type	I	S/N	Chromatogram	Max. m/z	FWHM [min]
n.a.	5.8	n.a.	Single spectrum	n.a.	n.a.	n.a.	475.3285	n.a.

+MS, 5.8min #350

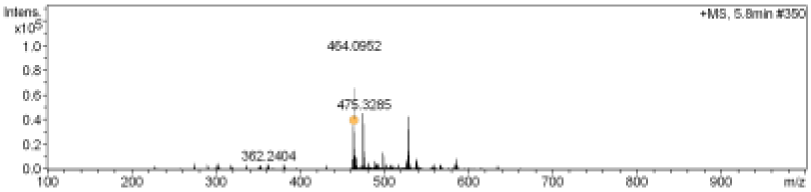

Meas. m/z	#	Ion Formula	m/z	err [ppm]	mSigma	# Sigma	Score	rdb	e⁻ Conf	N-Rule
464.0952	1	C24H17BClN3NaO3	464.0948	1.0	36.6	1	100.00	17.5	even	ok

Figure S52. HRMS (ESI$^+$) report of **4j**

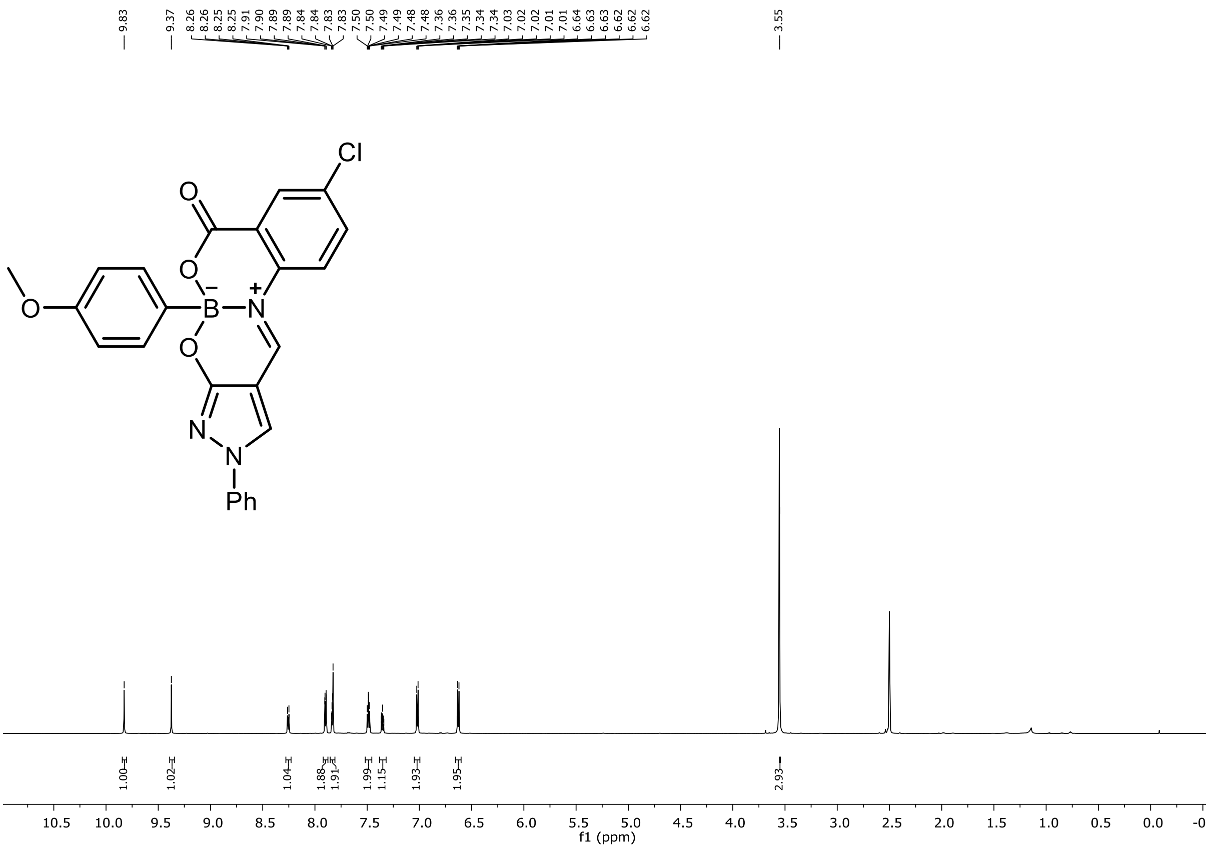

Figure S53. ^{1}H NMR (700 MHz, DMSO-d_6) spectrum of **4k**

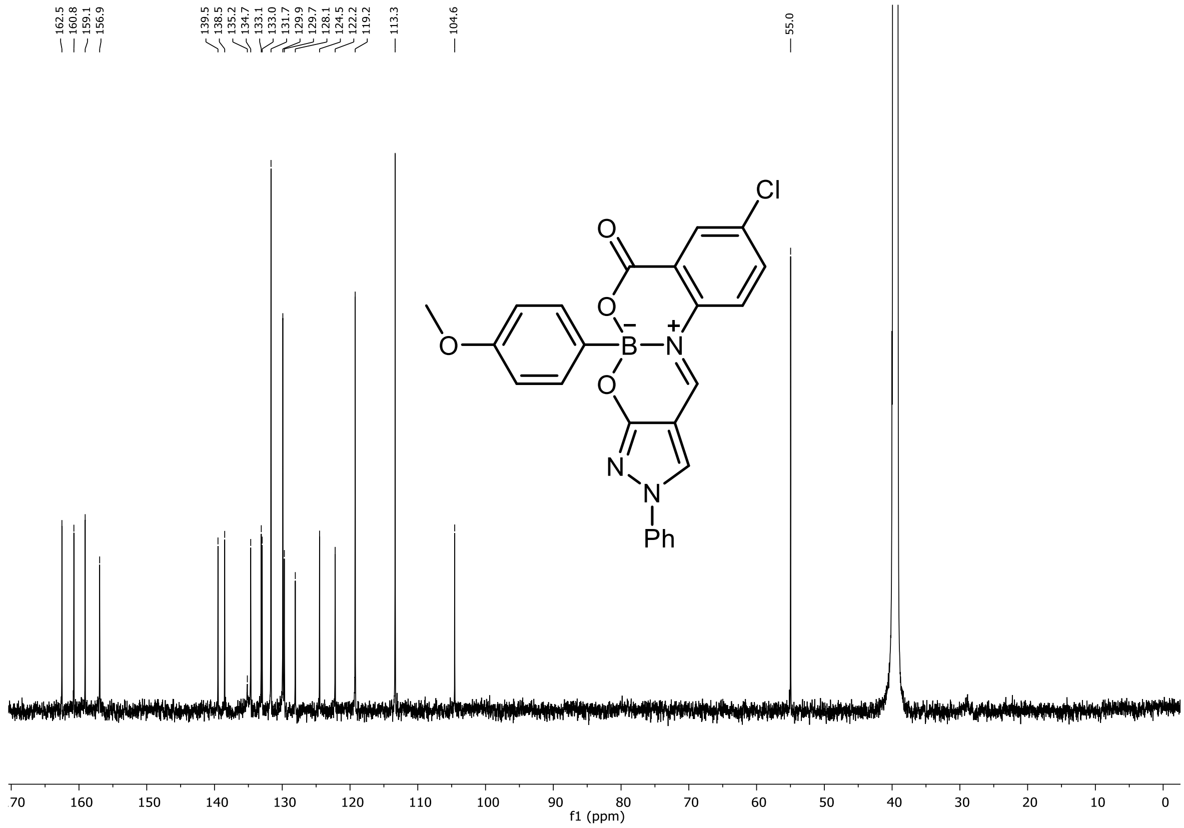

Figure S54. ^{13}C NMR (176 MHz, DMSO-d_6) spectrum of **4k**

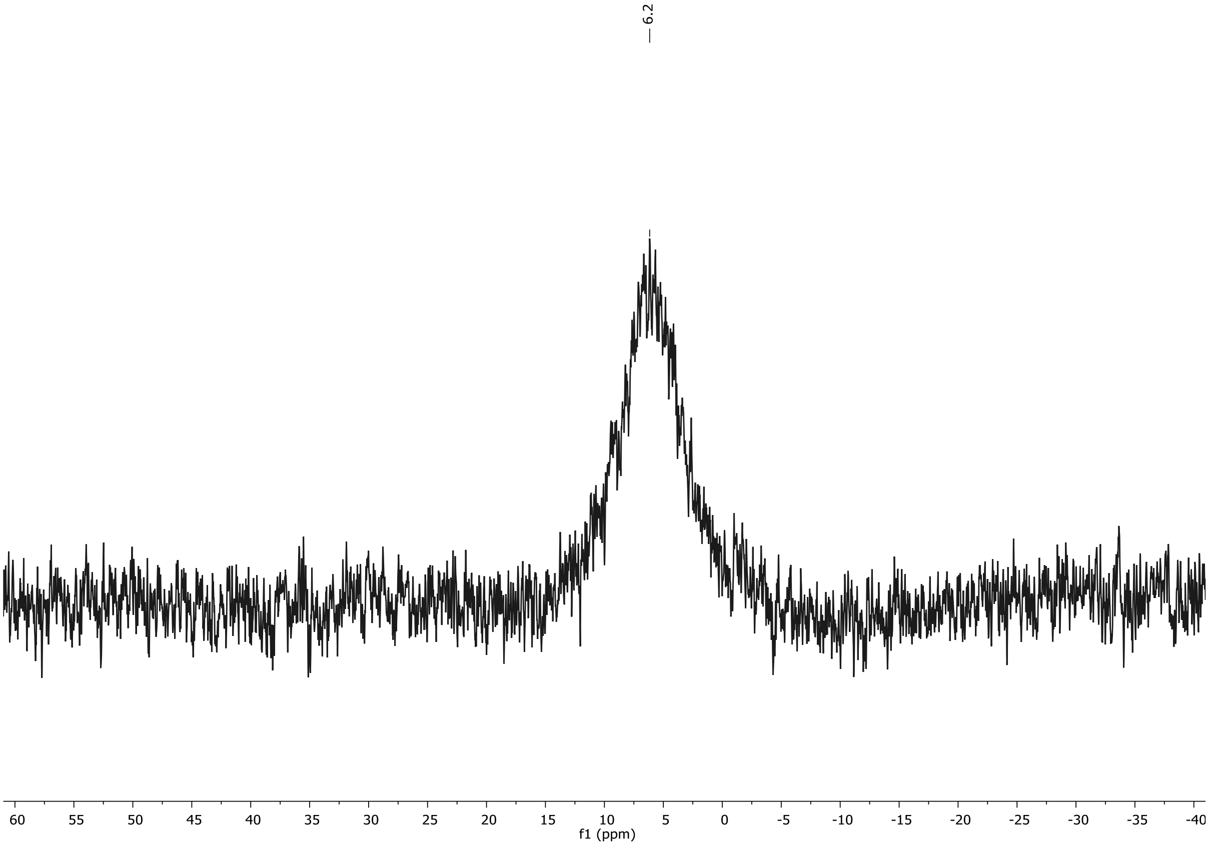

Figure S55. ^{11}B NMR (128 MHz, DMSO-d_6) spectrum of **4k**

Compound Spectrum SmartFormula Report

Analysis Info

Analysis Name	D:\Data\VDD-243.d	Acquisition Date	3/1/2024 8:32:46 PM
Method	DirectInfusion_TuneLow_pos.m	Operator	hplc
Sample Name	VDD-243	Instrument	micrOTOF-Q III 8228888.20448
Comment	AB		

Acquisition Parameter

Source Type	ESI	Ion Polarity	Positive	Set Nebulizer	0.4 Bar
Focus	Not active	Set Capillary	4500 V	Set Dry Heater	180 °C
Scan Begin	50 m/z	Set End Plate Offset	-500 V	Set Dry Gas	4.0 l/min
Scan End	1000 m/z	Set Collision Cell RF	140.0 Vpp	Set Divert Valve	Waste

#	RT [min]	Area	Int. Type	I	S/N	Chromatogram	Max. m/z	FWHM [min]
n.a.	7.7	n.a.	Single spectrum	n.a.	n.a.	n.a.	528.5131	n.a.

+MS, 7.7min #464

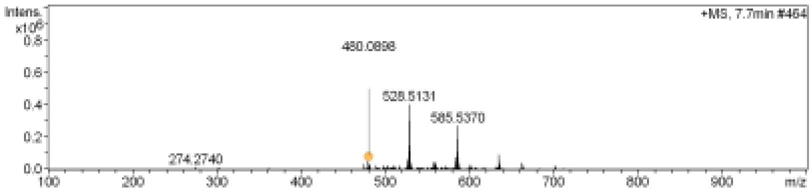

Meas. m/z	#	Ion Formula	m/z	err [ppm]	mSigma	# Sigma	Score	rdb	e⁻ Conf	N-Rule
480.0898	1	C24H17BClN3NaO4	480.0897	0.1	46.6	4	100.00	17.5	even	ok

Figure S56. HRMS (ESI$^+$) report of **4k**
